# Diagenetic and Biological Overprints in Geochemical Signatures of the *Gigantoproductus* Tertiary Layer (Brachiopoda): Assessing the Paleoclimatic Interpretation

**DOI:** 10.3390/life13030714

**Published:** 2023-03-06

**Authors:** José R. Mateos-Carralafuente, Ismael Coronado, Juncal A. Cruz, Pedro Cózar, Esperanza Fernández-Martínez, Sergio Rodríguez

**Affiliations:** 1 Department of Geodynamics, Stratigraphy and Paleontology, Faculty of Geological Sciences, Complutense University of Madrid, c/ José Antonio Novais, 12, 28040 Madrid, Spain; 2 Facultad de Ciencias Biológicas y Ambientales, Universidad de León, Campus Vegazana s/n, 24071 León, Spain; 3 Instituto de Geociencias (CSIC, UCM), c/ Severo Ochoa 7, 28040 Madrid, Spain

**Keywords:** LPIA, microstructure, trace-element partitioning, stable-isotope fractionation, Serpukhovian, Spain

## Abstract

Variations in the geochemical signatures of fossil brachiopod shells may be due to diagenesis and/or biological processes (i.e., ‘vital effects’). It is critical to characterise them in order to identify reliable shell areas suitable for paleoclimate studies. This investigation contributes to an in-depth understanding of geochemical variations in *Gigantoproductus* sp. shells (SW Spain, Serpukhovian age), throwing light onto the Late Paleozoic Ice Age interpretation. Microstructural, crystallographic, cathodoluminescence and geochemical (minor and trace elements, δ^18^O, δ^13^C, and strontium isotopes) characterisations have been performed on the tertiary layer of the ventral valve, to assess the preservation state. Poorly preserved areas exhibit microstructural and geochemical changes such as recrystallisation, fracturing and higher Mn and Fe enrichment. Moreover, these areas have a higher dispersion of ⁸⁶Sr, ⁸⁷Sr, δ^18^O and δ^13^C than well-preserved areas. Three structural regions have been identified in well-preserved areas of the ventral valve by differences in valve curvature and thickness, such as the umbonal and thick and thin regions. These regions have different proportions of Mg, S, Na, δ^18^O, and δ^13^C, which are interpreted as ‘vital effects’ and probably related to growth-rate differences during shell growth. The *Gigantoproductus* tertiary layer seems the most suitable for paleoclimate studies, because it retains the original microstructure and geochemical composition.

## 1. Introduction

The Late Paleozoic Ice Age (LPIA), elapsed from the Viséan to the Capitanian (~335 to 260 Ma) [1], was a dynamic climatic phenomenon that involved multiple glacial and interglacial events driven by the continental Pangea assembly, and low global atmospheric carbon dioxide concentrations (*p*CO_2_). These constraints, in turn, produced glacio eustatic variations, changes in ocean circulation, and finally, a turnover in marine flora and faunas [1,2,3,4,5,6,7,8]. The Mid-Carboniferous glacial peak represented an important change in seawater chemistry and ocean circulation [9] and led to one of the most important and selective crises in the Phanerozoic Era [10]. Low speciation and high extinction rates of marine faunas are the outstanding features during this period, particularly in the late Serpukhovian [11].

Most of the palaeoclimatological Carboniferous data are based on carbonate brachiopod δ^18^O and δ^13^C signatures, as paleotemperature and carbon-cycle proxies, respectively [9,12,13,14,15,16,17,18,19,20,21,22,23,24]. Rhynchonellid brachiopod shells are commonly selected for Carboniferous paleoclimate reconstructions, due to their worldwide distribution and abundance [13,19,20,21,25,26,27,28]. Carbonate brachiopod shells are composed of low-magnesium calcite (LMC), a polymorph which is relatively resistant to diagenesis [29].

Gigantoproductids (tribe Gigantoproductini [30]) are key, due to their great potential for palaeoclimatological studies because: (i) they were frequent during the Carboniferous [28,31,32]; (ii) they had global distribution [30]; (iii) they had large shell thickness and size [33], which allow the extraction of material for geochemical analyses more easily than with other fossil brachiopods [28,34,35]. Large shell size has been attributed to a possible chemosymbiotic lifestyle [35]. In spite of these advantages, few studies have focused on this fossil group for palaeoclimatological purposes [9,17,22,28,34]. These studies reported geochemical variations in gigantoproductid shells regarding other brachiopod genera, for example higher contents of Mg, S and Na than *Martinia* and *Chorisitites* [17,28]. Moreover, large fluctuations in stable isotopes (δ^18^O and δ^13^C) have been described in gigantoproductid shells concerning other brachiopod genera, such as *Chorisitites,* of the same age and from the same localities [28]. These variable compositions detected in gigantoproductuid shells raise several hypotheses about their origin: (i) they could be the result of diagenesis; (ii) they could be caused by kinetic and biological effects during shell growth; (iii) they could be result of a combination of both processes.

Despite the relative resistance of the brachiopod shells to diagenesis, it may obliterate the original microstructure and modify the shell geochemistry, making the palaeoclimatological interpretation difficult. In order to recognize diagenetic alteration, the identification of non-altered and altered areas in the brachiopod shell is needed. Altered regions are usually enriched or depleted in some trace elements such as Mn, Fe, Sr, and Na [36]; they exhibit isotope outliers; they show luminescence under cathodoluminescence [17], although non-luminescence is not always indicative of good preservation [37]; there is a lack of microstructures [36]; and the crystallographic orientation of biomineral structures is altered [38].

Despite the outstanding role of brachiopods shells in palaeoclimatological studies, some researchers have identified ‘vital effects’, considered herein as significant geochemical deviations from the thermodynamic equilibrium of seawater [39]. These variations have been recognized in extant brachiopods [40,41,42,43] and in well-preserved areas of fossil brachiopods (e.g., [22,24,34]). These geochemical variations in biominerals can be ascribed to: (i) biological processes [44] related to the metabolism and physiology of the organism (named ‘true vital effects’ by Pérez-Huerta and Andrus [45]); and (ii) bio-crystallization processes related to mechanisms of non-classical precipitation of biominerals [46,47]. In addition, both categories might also be affected by environmental factors such as temperature, salinity, and ocean acidification [46,48]. The selection among these hypotheses is complicated, and even an impossible process, due to the ‘vital effects’, which are currently poorly known mechanisms [45]. Geochemical variations derived from brachiopod shell growth are included within the category of biological processes such as kinetic effects or metabolic effects, including metabolic prioritization [24,40,42,49].

Growth rate and stable-isotopic fractionation covariation have been identified in δ^18^O and δ^13^C in extant and fossil brachiopods [24,35,40,49,50,51,52]. δ^18^O and δ^13^C variations between shell layers have been documented in extant and fossil brachiopods, and are considered as kinetic effects of the shell growth [24,43,49,50,51]. The tertiary layer is often selected for palaeoclimatological studies, due to its slower growth rate compared to the primary and secondary layers [9,28,34,36,43].

On the other hand, tailored δ^26^Mg fractionation has been detected between the primary and secondary layer in extant brachiopods [42]. Shell regions with higher growth rates are usually characterized as being ^26^Mg-enriched and ^13^C- and ^18^O-depleted [42]. Therefore, a covariation between growth rates and ion partitioning (e.g., minor and trace elements, MTE, such as Mg, Na, and S) has been identified in extant brachiopods [42], in which a heterogeneous Mg intra-shell distribution is described. Grossman et al. [36] described a covariation between Na and S from the inner to outer shells of fossil brachiopods, related to growth rates, with higher Na and S contents during higher growth rates. Moreover, a wide-ranging Mg variation in extant brachiopod shells has been documented [53,54,55]. The amount of MgCO_3_ in brachiopod shells varies ontogenetically during growth from Mg-enriched younger zones to progressively depleted older zones [55,56,57,58]. The Mg variation has a direct influence on the Mg/Ca ratio for the paleotemperature calculation, and needs further studies to establish robust knowledge of brachiopod growth rate and element covariation. This background highlights the need for a detailed characterization of the original microstructure and geochemistry of fossil brachiopod shells, prior to assessing the geochemical variation produced by diagenetic alteration and ‘vital effects’ [29,36].

The main aims of this study are: (i) to characterize the biogenic and diagenetic features of the tertiary layer of the *Gigantoproductus* sp. ventral valves (SW Spain, Serpukhovian Age), using a combination of structural, geochemical, and crystallographic techniques; (ii) to establish the most favorable zones for geochemical sampling, avoiding palaeoclimatological misinterpretations.

## 2. Geological Setting

**Figure 1:**
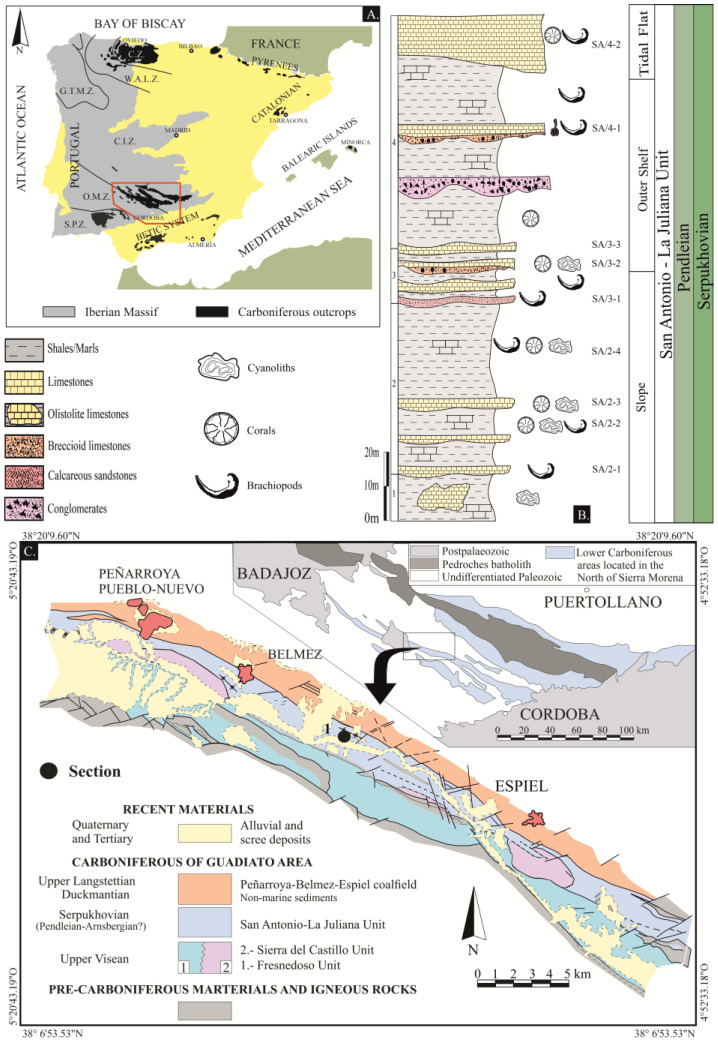
(**A**) Synthetic map showing the distribution of the main Carboniferous outcrops of the Iberian Peninsula, modified from Colmenero et al. [65]. (**B**) Stratigraphic log of the San Antonio section with geographic coordinates 5°8′20″ W–38°14′ N, modified from Cózar [63] and Cózar et al. [62]. (**C**) Early Carboniferous outcrops (upper Tournaisian to Arnsbergian) in Sierra Morena (upper part) and synthetic map with the main geological units of the Guadiato Area (lower part). Modified from Cózar [63]). Cantabrian Zone, C.Z.; West-Asturian-Leonese Zone, W.A.L.Z.; Galicia-Tras os Montes, G.T.M.Z.; Central-Iberian Zone, C.I.Z.; Ossa-Morena Zone, O. M. Z. and South Portuguese Zone, S.P.Z.).

The studied specimens were collected in the Guadiato Valley, coordinates 5°8′20″ W–38°14′ N, [59], a NW-SE elongated Carboniferous outcrop included in the Sierra Morena, at the southern Iberian Massif (Figure 1). Gigantoproductid samples were collected from the San Antonio section (San Antonio-La Juliana Unit), assigned to the Pendleian, Serpukhovian [60,61].

The Guadiato Valley (Figure 1) is an elongated zone where Carboniferous outcrops (late Viséan to early Westphalian) are separated by WNW–ESE faults [61]. It is divided into three tectono-sedimentary marine and paralic units: the Fresnedoso Unit, formed by siliciclastic rocks of the Viséan Age; the Sierra del Castillo Unit, formed mainly by carbonates of the Viséan Age, and the San Antonio-La Juliana Unit, formed by siliciclastics and carbonates of the Serpukhovian Age [59].

The San Antonio-La Juliana Unit has been interpreted in origin as a strike-slip basin with syntectonic sedimentation. It is composed of terrigenous and carbonate rocks and fossils of marine and paralic origin, which belong to several geographically close sedimentary environments. It comprises slope facies (hemipelagic sediments, olistoliths, debris flows and turbidites), platform facies (internal platform, tempestites with shallowing episodes), tidal-plain facies (intertidal plain and small lakes) and deltaic facies [60,62].

The San Antonio section, 146 m thick, is formed mainly by shales and siltstones with intercalated limestone and calcareous marlstones (Figure 1). The succession is open marine, with slope facies at the base that evolve into platform facies and deltaic facies in the upper part. The paleontological content is mostly represented by large-sized crinoids, gigantoproductids, bryozoans, rugose and tabulate corals, cyanoliths [62,63] and conodonts [61]. Based on foraminifera and conodonts, the Age is Pendleian (Early Serpukhovian; [61,64]).

## 3. Materials and Methods

### 3.1. Studied Material

Eleven specimens of *Gigantoproductus* sp. (Figure S1 in Supplementary Material) have been selected for this study, from the 2–4 horizon of the San Antonio section (Figure 1). The material (DMP-A301-1014-1 to DMP-A301-1014-11) is housed in the Paleontological Collections of the Paleontology Area (Complutense University of Madrid, UCM). The specimens were sectioned in two halves longitudinally, along the sagittal plane from the umbo to the commissure, when possible, and each slab was polished using sandpaper and 1 μm and 0.3 μm alumina. Polished samples were scanned in order to cross reference digitally the macroscopic features of the shells. One slab of each of the 11 specimens has been used for this study, and the other 11 slabs have been reserved for future studies. A total of twenty thin sections and five ultra-thin sections were prepared (*sensu* Coronado et al. [66]).

### 3.2. Microscopy Methods

The specimens were studied under a petrographic microscope, using scanning electron microscopy (SEM), computer-integrated polarization microscopy (CIP) and cathodoluminescence microscopy (CL), in order to select well-preserved areas for subsequent drilling.

A petrographic microscope, LM Leica DMLP, with a coupled camera Leica DC 300, was used for the purpose of characterizing the microstructure of the brachiopod shell.

Samples were extracted by natural breakage from the ventral valves of *Gigantoproductus* sp. between the muscle scars and the commissure, perpendicular and parallel to the valve growth. Moreover, a complete shell section was polished and etched in a 5% HCl solution for 20–25 s. These samples were coated with gold and analyzed by scanning electron microscopy (SEM), using a model JEOL JSM-820 working at 20 kV, located in the research facility of Geological Techniques of Complutense University of Madrid (UCM, Spain), in order to complete the microstructural characterization.

Thin sections were photographed under CL working at 16 kV and a current of 0.5 mA, in order to assess the intensity and distribution of luminescence. A cold cathodoluminescence probe model 8200 MK4 of Cambridge Image Technology Ltd. attached to a petrographic microscope model Eclipse E400 POL, with a camera, was used for this purpose. This microscope is located in the Geological Storage Division, Hydro-geochemical Group Ciemat (Madrid, Spain).

In order to evaluate shell preservation, the crystallographic organization of brachiopod shells was studied using computer-integrated polarization microscopy (CIP, [67]). This method for texture analysis and optical-orientation imaging has been applied in biomineralization studies of fossil bio-calcite with relevant crystallographic and structural results, highlighting it as a robust tool in diagenetic characterization [68,69,70]. This method determines the *c*-axis orientations of uniaxial minerals (such as calcite and quartz) from optical micrographs, displaying the results in pole figures and orientation images, using an RGB color-code that represents each orientation.

Seven CIP analyses in different samples and regions of the shell were performed, using an experimental petrographic Zeiss microscope with an automated rotation, and tilting system, made with the Arduino UNO microcontroller (open-source hardware) and controlled with software implemented in the LabVIEW environment (details in Coronado and Rodríguez [71]). The lowest ratio of pixel–µm reached with this setting in the micrographs is 1:0.05. Finally, the micrographs were processed using Image SXM software [72].

Crystal sizes were measured via micrographs using the plug-in ObjectJ 1.03w [73] of the free and open-source ImageJ 1.47v image-processing software [74].

### 3.3. Trace Elements and Isotope Analyses

Seven elements (Ca, Mg, Sr, S, Na, Mn and Fe) were analyzed using an electron microprobe analyzer (EMPA), with the analysis conducted on eleven thin sections (364 punctual analyses, 20 or 25 points per thin section), using a JEOL Superprobe, JXA-8900M with five wavelength-dispersive spectrometers, located in the Spanish National Centre for Electron Microscopy of the Complutense University of Madrid. Each point was analyzed with an accelerating voltage of 15 kV, a beam current of 10 nA and a spot size of 5 μm. Each analysis took an acquisition time of 45 s; because five elements can be analyzed simultaneously, the total time for analysis of the seven elements was 90 s. The following standards and detection limits were used in the EMPA: Ca: 201.5 ppm, Mg: 181.25 ppm (dolomite); Fe: 418.5 ppm, Mn: 439.5 ppm (siderite); Sr: 262.25 ppm (strontianite); Na: 167.75 ppm (albite) and S: 235.25 ppm (galena). In addition, the same seven elements were mapped. The EMPA mapping enables simultaneous analysis of different elements and the generation of distribution images for each element with 1 µm resolution. An accelerating voltage of 20 kV with a beam current of 100 nA and a spot size and step interval of 1 µm diameter (dwell time = 25 ms) were used.

Samples were powdered using a microdrill with an x-y micrometric plate attached to a binocular and a 500 μm tungsten carbide drill in selected areas, sampling for stable isotope analysis (δ^18^O, δ^13^C) and ^87^Sr/^86^Sr measurements. Stable isotope sampling requires only one drill hole to obtain enough material, whereas ^87^Sr/^86^Sr requires 30 to 40 drill holes.

Thermal ionization mass spectrometry (TIMS) was used in order to date the samples of study from the ^87^Sr/^86^Sr ratio. A total of 7 mg was obtained by the drilling of 4 samples of well-preserved zones and 2 samples of poorly preserved zones. The material was processed to obtain a Sr concentrate residue [75], which was loaded onto a Re filament by adding 1 μL of H_3_PO_4_ 1M 4 and 2 μL of Ta_2_O_5_. The Sr isotopic ratios were analyzed on a TIMS-Sector 54^®^ Mass Spectrometer, located in the Laboratory of Geochronology and Isotope Geochemistry (UCM), following a dynamic multicollection data-acquisition method for 10 blocks of 16 cycles each, with beam intensity mass of ^88^Sr at 3 V. The Sr analyses were corrected, to avoid possible interferences of ^87^Rb. The ^87^Sr/^86^Sr ratios were normalized with respect to the measured value of the ratio ^86^Sr/^88^Sr = 0.1194, in order to correct the possible fractionation of masses that the sample might have undergone during the filament loading and/or instrumental analysis. During the analysis of the samples, the isotopic standard of Sr (NBS 987) was analyzed repeatedly, and the following values were obtained: 0.710239 ± 1.7 × 10^−5^ (*n* = 8). These values were used to correct the analyses, taking into consideration the plausible drift referred to as the standard, and the certified value of the standard.

Stable isotope analyses (δ^18^O and δ^13^C) were undertaken using a triple-collector isotope-ratio mass spectrometer, Finnigan MAT 253 of the Stable Isotope Laboratory of the Department of Geological Sciences of the University of Michigan (USA). Forty-four samples were drilled, with 0.1 mg of each obtained, 30 from well-preserved zones and 14 from zones with evidence of poor preservation, previously characterized using optical microscopy, cathodoluminescence and CIP, and weighted and digested using H_3_PO_4_ at 77° ± 1 °C for 8 min in a Finnigan MAT Kiel IV. The analyses were calibrated using a better-fit regression line defined by two international standards, the NBS 18 (National Bureau of Standards; δ^13^C = −5.014‰ and δ^18^O = −23.2‰) and the 19 (National Bureau of Standards; δ^13^C = 1.95‰ and δ^18^O = −2.20‰). The data are given in ‰ notation, relative to the VPDB (Vienna Pee Dee Belemnite). The accuracy of the data was monitored through daily analyses, using a variety of carbonate-powder standards. At least 4 carbonate standards were reacted, analyzed and measured with an accuracy below ±0.1‰ for δ^13^C and δ^18^O.

## 4. Results

Collected specimens from the San Antonio section belong to horizons 2–4, which make them unsuitable for correlating geochemical variations with environmental factors (e.g., paleoseasonality). In order to differentiate diagenetic and biological overprints in *Gigantoproductus* shells, results have been divided into three main sections: shell microstructure, fossil preservation and geochemical characterization.

### 4.1. Shell Microstructure

**Figure 2:**
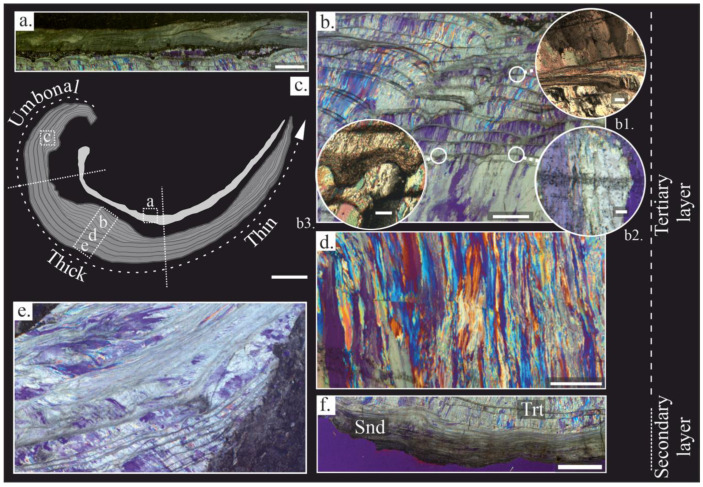
(**a**–**e**) Microstructural features of *Gigantoproductus* sp. Shells under petrographic microscopy, cross-polarized micrographs; (**a**,**b**,**d**–**f**), and cross-polarized with lambda plate micrographs. (**c**) Scheme of the shell with underscored shell regions. On the right, a line which divides the secondary and tertiary layer into the thick region (micrographs (**b**,**d**,**f**)). (**a**) The laminar microstructure of the dorsal valve with micritized patches (upper part), micrite infilling between the dorsal and the ventral valves, and inner region of the ventral valve (lower part). Scale bar 2 mm. (**b**) Innermost part of the thick region, showing ventral valve with columnar microstructure and several growth lines. Scale bar 1 mm. (**b1**) Inset showing a laminar growth line. Scale bar 100 µm. (**b2**) Inset showing a diffuse growth line. Scale bar 100 µm. (**b3**) Inset showing pseudopunctae in a growth line. Scale bar 100 µm. (**d**) Umbonal region of the ventral valve with several growth lines and a chaotic crystal arrangement. Scale bar 1 mm. (**e**) The thick region of the ventral valve with the largest columnar crystals and several growth lines. Scale bar 1 mm. (**f**) Thin region of the ventral valve with columnar microstructure and several growth lines. Scale bar 1 mm.

Gigantoproductids have a concave-convex shell composed of a dorsal and ventral valve. The dorsal valve is thinner than the ventral valve. It has a mostly laminar microstructure, with lath crystals (*sensu* [76]), ~1–2 μm in width, grouped in laminae (*sensu* [34,76,77]) ~10 to 20 μm thick (Figure 2a); a few columnar layers, with smaller crystals than the ventral valve, appear within the laminar microstructure. The crystal size decreases towards the inner part of shell, as was recognized in the ventral valve.

The dorsal valve (Figure 2a) shows a similar thickness (~2–3 mm), which decreases towards the commissure, whereas the ventral valve has a noticeable thickness difference. The shell shape can be divided into three regions of the ventral valve based on the thickness differences: the umbonal region (U-region), the thick region (Tk-region) and the thin region (T-region) (Figure 2). The first region is more incurved than the rest of the shell, and is of ~8–10 mm in thickness, the second one is a thickened area, ~13–22 mm, following the U-region, and the third one is more planar than the U and the Tk region, with a ~5–7 mm thickness, which decreases from the thick region to the commissure.

**Figure 3:**
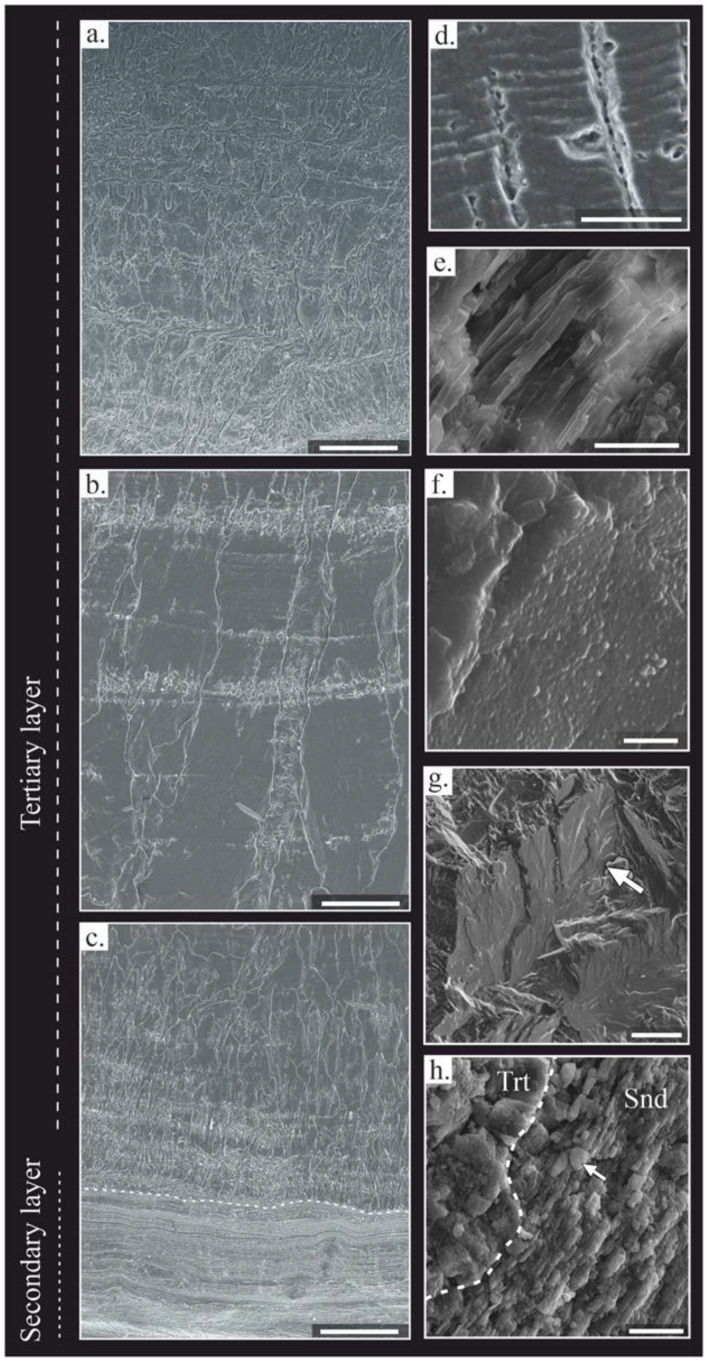
SEM images of polished shell surfaces (**a**–**d**). SEM images of natural breakage of the shell (**e**–**h**). (**a**) Innermost part of the tertiary layer. Scale bar 200 μm. (**b**) Middle part of the shell. Scale bar 200 μm. (**c**) Outermost part of the tertiary layer and secondary layer. Scale bar 200 μm. (**d**) Submicrometric laminae preserved in a columnar crystal. Scale bar 10 µm. (**e**) Laminar microstructure composed of lath crystals. Scale bar 10 µm. (**f**) Nanogranular texture in some columnar crystals. Scale bar 2 µm. (**g**) Columnar crystals of the tertiary layer; notice the massive appearance. Scale bar 100 μm. (**h**) Contact between columnar and laminar microstructure (dashed line) in a growth line with a granular appearance of laminae. Scale bar 10 µm. Trt: tertiary layer, Snd: secondary layer.

In the ventral valve, two layers were distinguished: (i) the secondary or laminar layer and (ii) the tertiary or columnar layer. The laminar is formed by lath crystals, ~1 μm in width, arranged in packages of thin sheets ~10 to 40 μm in width (Figure 2f). The crystals are mostly tabular in appearance, with sharp contacts between the crystals (Figure 3e); the tertiary or columnar layer is formed of large columns (*sensu* [78]), with sizes ranging from 100 to 1700 μm in length and 40 to 150 μm in width (Figure 2e). Occasionally, the tertiary layer shows intercalation of laminar growth lines with a similar microstructure to the secondary layer embedded between the columns. The columnar crystals are composed of submicrometric steps stacked parallel to their growth direction (Figure 3d), forming terraces in the natural breakage (Figure 3g). Massive crystals growths in the columns occur commonly in the thick region (Figure 2e and Figure 3b).

Likewise, two types of microstructural changes parallel to the shell growth were identified in the tertiary layer, called growth lines: (i) a dashed and diffuse aspect (Figure 2(b2)) with several dark dots (i.e., opaque under transmitted light and in BSE images), passing through the columnar crystals, ~50 to 100 μm of thickness; (ii) a laminar microstructure (Figure 2(b1)), densely packed and with ~30 to 80 μm of thickness. These growth lines cut off the columnar crystals. A variation of the laminar microstructure with a granular appearance is observed in these growth lines.

### 4.2. Fossil-Shell Preservation

Fossil preservation has been addressed herein by the combination of cathodoluminescence, textural changes under both optical microscopy and SEM, the crystallographic arrangement of microstructures and the geochemical signatures (i.e., minor and trace elements and Sr isotopes). This set of techniques was applied to distinguish between well-preserved and poorly preserved shell regions, with the aim of comparing the luminescence and geochemical differences between the different shell regions and their relationship with the microstructure.

#### 4.2.1. Textural Changes

Microtextural changes were recognized in some areas of the intra-shell surface, due to diagenetic processes. Fractures of the shell are usually oriented perpendicularly, or at a high angle to the shell surface, starting from the inner shell and moving to the outer shell, and are usually filled with micrite, sparite or iron oxides. On the other hand, thinner fractures, with micrite filling, parallel to the shell surface were observed in the center of some ventral valves. Some samples expose delamination processes near the shell surface.

Changes in the crystal size and the orientation of the tertiary layer are produced in adjacent zones of fractures or near to the edges of the shell. These areas contain a decreasing trend in crystal size towards the outer shell, with a mosaic appearance. Some areas exhibit micritization of the secondary layer and degrading neomorphism in the tertiary layer. These recrystallized zones are recognized by small and equigranular crystals, which totally obliterate the primary microstructure. Moreover, other zones contain partially dissolved crystals with a cloudy appearance, where the primary microstructure is roughly recognized. Rounded dark dots are opaque inclusions and are mostly concentrated in growth lines (Figure 2(b2)), whereas other scattered inclusions have sharp edges and different interference colors.

#### 4.2.2. Crystallographic Arrangement

The observation of crystallographic patterns is a precise approach for the evaluation of diagenesis of polycrystalline skeletons formed by microcrystals, with preferred crystallographic orientations and a notable arrangement of supra-specialized structures [29]. This criterion has been previously applied to brachiopod shells, achieving excellent results in experimental diagenesis and in the evaluation of fossil shells [79,80,81]. In addition, crystallographic assessments of other calcium carbonate skeletons have been successful [65,67,68]. This approach is based on the principle that controlled mineralized shells have constrained crystallographic arrangements (i.e., preferred orientations), which, after diagenesis, can be compromised and substituted by random orientations and/or loss of textural features.

**Figure 4:**
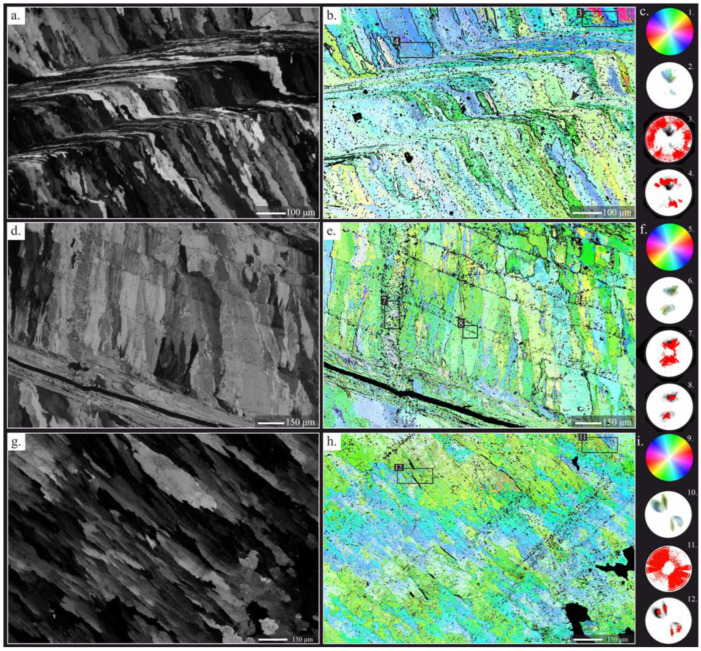
CIP analysis of thin regions of *Gigantoproductus* ventral valves (**a**,**d**) and thick region (**g**). Orientation images (**b**,**e**,**h**). Petrographic micrographs of the studied areas (**a**,**d**,**g**). Standard colour lookup table (CLUT) and pole figures (**c**,**f**,**i**). CLUT of the orientation images (1, 5, 9). Pole figures (2–4, 6–8, 10–12). (2) Corresponds to the complete area (**b**), showing a pole maximum with *c*-axis which is strictly oriented perpendicular to the growth direction of *Gigantoproductus* shell. (3–4) Corresponds to selected areas. (3) Columnar area with random *c*-axis orientations due to recrystallisation. (4) Equivalent area to (3), showing a well-constrained *c*-axis orientation. (6) Corresponds to the complete area (**d**). (7–8) Corresponds to selected areas. (7) Fracture with degrading neomorphism, which preserves the original crystallographic orientation. (8) Equivalent area to (7), showing a better constrained *c*-axis orientation. (10) Corresponds to the complete area (**g**), showing a pole maximum, with *c*-axis which is strictly oriented perpendicular to the growth direction of the *Gigantoproductus* shell. (11–12) Corresponds to selected areas. (11) Prismatic area with random *c*-axis orientations, due to recrystallisation. (12) Equivalent area to (11), showing a well-constrained *c*-axis orientation. Pole figures were calculated as an orientation-distribution function and are provided in multiples of uniform distribution intervals of 0.5 for *c*-axis orientations of the complete area. Red points correspond to the punctual *c*-axis orientation of each pixel of selected areas.

Seven areas of five ultra-thin sections of *Gigantoproductus* sp. ventral valves were studied after sampling for geochemical analysis (Figure 4). The CIP method is limited to assessing the crystallographic organization based on azimuth and inclination of the *c*-axis. Criteria to identify diagenetic alterations are based on abrupt changes in the *c*-axis orientation (highly disoriented), and textural changes with or without subtle *c*-axis variations.

The *c*-axis is strictly oriented perpendicular to the growth direction of *Gigantoproductus* shells (i.e., parallel to the radial axis of the valve), regardless of microstructure, shell region and texture, although some variations have been recognized. Columnar and laminar microstructures exhibit different *c*-axis orientations: parallel to the elongation axis of the crystals in the case of the columnar microcrystals, and perpendicular in the case of the laminar crystals (Figure 4 and Figure S2). In those areas where the laminar microstructure changes gradually to columnar, and vice versa (e.g., in a growth line), the *c*-axis is kept constant between the interconnected crystals (Figure 4b), similar to kinked crystals (*sensu* [82]).

All the studied areas, which include all the shell regions identified, demonstrate preferred orientations (based on pole figures), with quite narrow azimuthal dispersion. However, some distinctive arrangements have been observed in some shell regions. Usually, *c*-axis orientations in the tertiary layer show two narrow pole maxima, with an azimuthal dispersion between them of ca. 55° in the umbonal region (Figure S2e) and ca. 35° in the thick region (Figure S2h,k). On the other hand, thin regions with columnar and laminar microstructures exhibit a single pole maximum with a variable azimuthal dispersion: between 35° and 42° in the thin region (Figure 4b,e and Figure S2b).

Evidence of diagenetic alteration based on crystallographic orientation and textural changes occurs in some areas. In those areas, where the original microstructure is apparently well-preserved and lamellar twinning is notable, they are characterized by random *c*-axis orientations in twinned crystals and the surrounding areas (with hundreds of microns, Figure 4b,h). These areas correspond mostly to thin fractures without displacement, favored by microstructural changes (e.g., columnar to laminar or contact between columnar crystals, Figure 4h). In addition, in some of these areas the original microstructure has been completely obliterated by recrystallisation processes (Figure S2b), and these areas exhibit random azimuthal orientations, forming large patches with similar characteristics. On the other hand, in some areas the surrounding fractures are characterized by a degrading neomorphism with co-oriented microcrystals, compared to the main crystallographic orientation of the shell (Figure 4e), although the azimuthal dispersion of orientations in this region is larger than in individual crystals (i.e., columnar, and laminar crystals). In Figure 4g,h the diagenetic alteration is confined by a growth line, and involves only a part of the underlying crystals. This area is recrystallized because the original texture is lost, and random crystallographic orientation occurs.

#### 4.2.3. Cathodoluminescence (CL)

**Figure 5:**
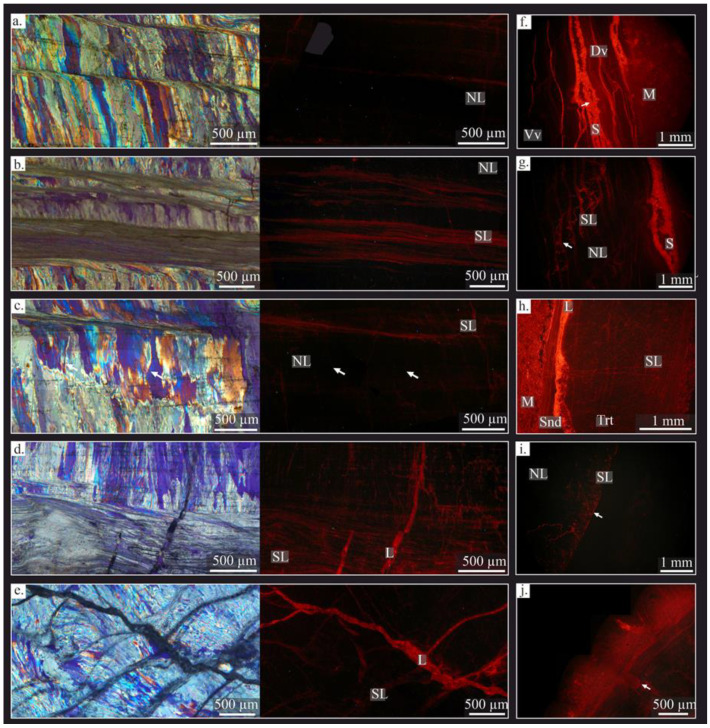
Fossil preservation. (**a**–**e**) Cross-polarized with lambda plate micrographs (left) and the same area under cathodoluminescence (right); (**f**–**j**) cathodoluminescence micrographs in other shell regions. (**a**) Non-luminescent well-preserved area under CL. (**b**) Well-preserved area with luminescence concentrated in growth lines. (**c**) Non-luminescent poorly preserved area with a recrystallisation patch in the center, evidence of recrystallisation. NL fracture (left arrow) (**d**) SL-luminescent poorly preserved area, with recrystallisation by fractures. (**e**) Highly luminescent poorly preserved area with fractures. (**f**) CL image showing a laminar growth line with the equivalent luminescent areas as in the fluorescent image. (**g**) Alteration patch in the ventral valve with altered growth lines, more luminescent than well-preserved areas. Luminescent sparite in the right of the image. (**h**) The altered external edge of the shell, more luminescent than the central and inner parts of the ventral valve. (**i**) Degrading neomorphism in the shell edge, with decreasing luminescence towards the interior. (**j**) Encrusting sponge over *Gigantoproductus* shell: notice the luminescent burrow (arrow). NL: non-luminescent, SL: slightly-luminescent, L: luminescent S: sparite.

Samples under CL exhibit almost exclusively non-luminescent areas (NL) (Figure 5), which correspond mostly to areas with well-ordered crystals of the tertiary layer (Figure 5a,c), some recrystallized zones (e.g., degrading neomorphism, Figure 5c), and fractures with iron-oxide cement (opaque under petrographic microscope, Figure 5b). Slightly luminescent areas (SL) are concentrated close to shell edges, in the laminar microstructure of the ventral and dorsal valves (Figure 5f,i), in the fracture zones with associated recrystallized areas (Figure 5d,e), in areas with degrading neomorphism (Figure 5i), in micritization zones close to the shell margin (Figure 5h), and in the growth lines (Figure 5g). High luminescent areas (L) correspond to the surrounding and infilling matrix (Figure 5f,h), the sparite cements of the inner space of the shell (Figure 5f,g), cemented borings (Figure 3j), large cemented fractures (Figure 5e,h), highly interconnected fractures or fracture zones (Figure 5g,h), and micritization areas close to the shell edge and some areas with neomorphism (Figure 5i).

The luminescence in the columnar crystals decreases from the edges to the inside (Figure 5), as well as in degrading neomorphism areas (Figure 5i). The luminescence of the growth lines increases in those areas affected by fractures, in comparison with the surrounding area (Figure 5d,g).

### 4.3. Geochemical Characterisation

Geochemical analyses, minor and trace-element (MTE) contents and isotope compositions, have been conducted in well- and poorly preserved areas, taking into consideration the luminescence under CL (Table 1, Figure 5), and the three shell regions identified (i.e., umbonal, thick and thin, Table 2, Table S2, and Table 3).

#### 4.3.1. Major/Minor and Trace Elements (MTE)

MTE helps to evaluate the degree of geochemical alteration of brachiopods by comparing cathodoluminescence, crystallographic arrangement and petrographic microimages. The *Gigantoproductus* ventral valve shows different trace-element concentrations related to diagenetic alteration, layers, and shell regions (Figure S3, Table 1 and Table 2).

The proportion of trace elements varies with the function of the preservation state (Figure S3, Table 1). In general, well-preserved areas of the ventral valve show lower mean standard deviation of the Ca, Mg and S values than the poorly preserved areas. The NL well-preserved areas contain more Ca, less Mg and less dispersion of all elements, except Sr, than the NL poorly preserved areas. The SL well-preserved areas contain more Mn and Sr, less S, and exhibit lower mean standard deviation of Ca and Mg than the SL poorly preserved areas. The highest mean of Mn occurs in the luminescent areas (Figure S3, Table 1).

The mol% of MgCO_3_/CaCO_3_ of the NL and SL well-preserved areas, for each shell region, represents a linear correlation (r = −0.98 p(a) > 0.05, *n* = 249). Moreover, the average values of well-preserved areas is 98.02 mol% CaCO_3_ and 1.677 mol%MgCO_3_, distinctive of low-Mg calcite. In addition, differences between regions are recognized in the U-region (98.33 mol% CaCO_3;_ 1.38 mol%MgCO_3_), Tk-region (98.48 mol% CaCO_3;_ 1.26 mol%MgCO_3_), and the T-region (98.03 mol% CaCO_3_; 1.68 mol%MgCO_3_).

Statistics per element (Table 2) of the different brachiopod-shell regions demonstrate a higher average concentration of Ca and Mn in the umbonal region than in the rest of the regions. The thick region (Tk) exhibits less proportion of Mg and Mn than the U- and T-regions, but more Ca. The T-region contains slightly more Mg, Sr, and S than the U- and Tk-regions, but lower Na. Mg and Sr average values are higher in the secondary layer than in the tertiary layer, whereas Ca is lower in the secondary layer.

**Figure 6:**
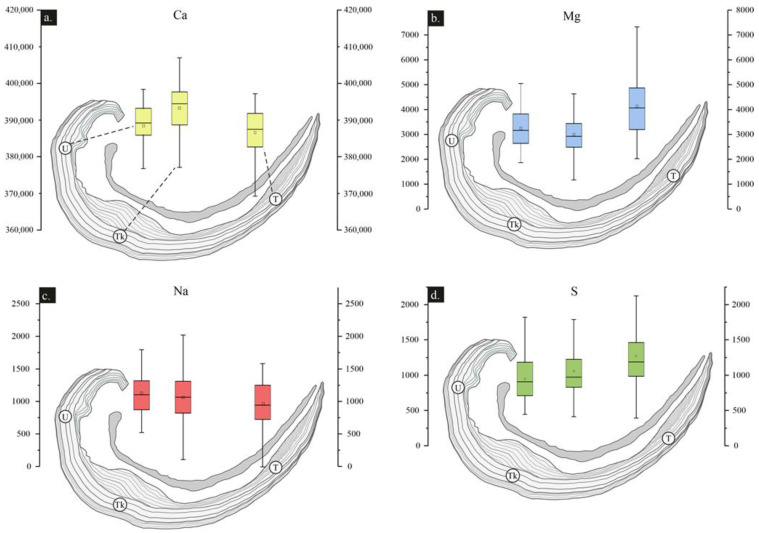
Box plot (**a**–**d**) showing the variation in Ca, Mg, Na and S (in ppm) for the proposed shell regions in *Gigantoproductus* ventral valve. U: umbonal region, Tk: thick region. T: thin region. Ca: calcium, Mg: magnesium, Na: sodium, S: sulphur. Outliers have been removed.

Ca, Mg, S and Na of all well-preserved areas are shown in boxplots in Figure 6. Mg is higher in the U- and T-regions, whereas Ca is higher in the Tk-region. Na is lower in the T-region; however, S is higher.

**Figure 7:**
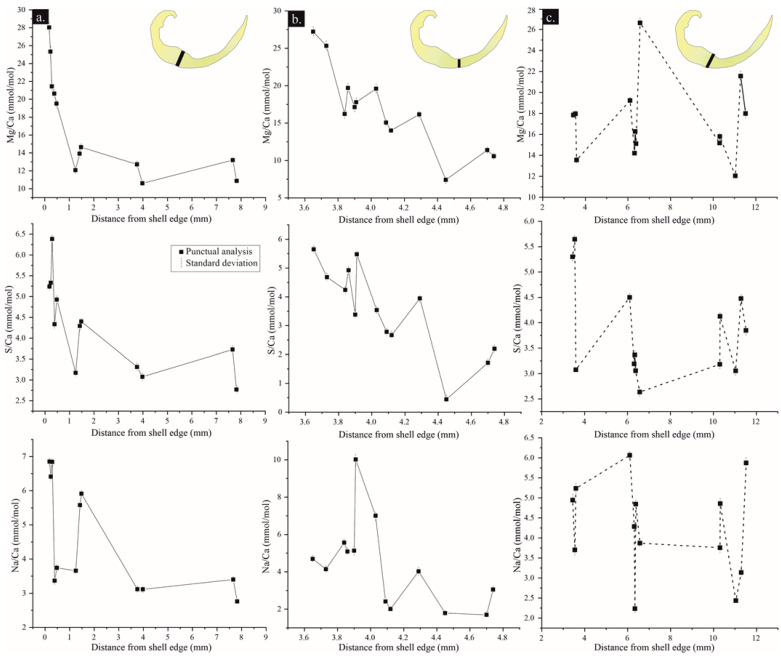
Mg/Ca, S/Ca, Na/Ca transects from the shell edge to the shell interior in well-preserved areas (**a**,**b**) and poorly preserved area (**c**).

The Mg/Ca, S/Ca and Na/Ca transects measured in well-preserved areas of three individuals varies, from a higher concentration near the shell edge to a decreasing one towards the shell interior (Figure 7). In addition, the concentration of Mg, S and Na covaries in the measured transects. In poorly preserved areas (e.g., recrystallized areas) a decreasing-metal/Ca trend is not observed, and each element is not positively correlated (Figure 7c).

#### 4.3.2. Isotope Geochemistry

The values of ^87^Sr/^86^Sr obtained from the TIMS range from 0.707830 to 0.707860 in the NL well-preserved areas (Table 3), while SL in the poorly preserved areas deviated from these values by ±0.00003. One sample is enriched in ^87^Sr, while another is depleted, in the cases of the well-preserved samples.

**Figure 8:**
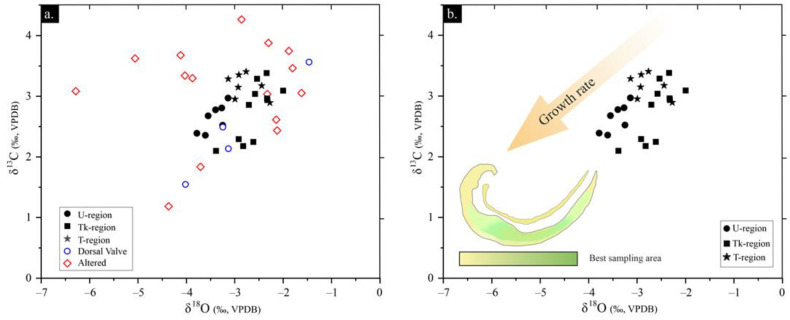
(**a**,**b**) δ^13^C vs. δ^18^O diagram of 11 *Gigantoproductus* shells. Well-preserved areas (filled-in black), dorsal valve (blue circles) and poorly preserved areas (red diamonds). (**b**) Values of well-preserved areas of the tertiary layer in each region. Plan of the shell with underscored shell regions. U: umbonal region; Tk: thick region; T: thin region; DV: Dorsal Valve.

The δ^13^C–δ^18^O relationship can be subdivided according to the dispersion degree into poorly preserved and well-preserved areas and the consistency within the identified shell regions (Figure 7; Table 3). Dispersed values, with a variation of ~4.6‰ for δ^18^O and of ~3‰ for δ^13^C, occur in poorly preserved areas, whereas ~1.7‰ for the δ^18^O and ~1.3‰ δ^13^C occur in well-preserved areas. Poorly preserved areas show different alteration signatures: some samples have similar δ^13^C but are depleted in δ^18^O, others are depleted in δ^13^C and δ^18^O, and others are δ^13^C- and δ^18^O-enriched (Figure 8; Table 3).

A correlation with each identified shell region is recognized in well-preserved areas (i.e., the same drilled regions contain rather similar values; Figure S5). Thus, the δ^18^O values of well-preserved areas range from −3.8 to −2‰ and from 2.1 to 3.4‰ for δ^13^C. The U-region is where the data shows the lowest δ^18^O, with averages of −3.4‰ for δ^18^O and 2.6‰ for δ^13^C (Table 3). The Tk-region exhibits similar δ^13^C data to that of the U-region, but lower δ^18^O, with averages of −2.6‰ for δ^18^O and 2.6‰ for δ^13^C. Higher δ^13^C values are recognized in the T-region compared to the U-region, with averages of −3.2‰ for δ^13^C and 2.7‰ for δ^18^O.

## 5. Discussion

In this section, the preservation of the tertiary layer of *Gigantoproductus* ventral valves and the influence of diagenesis on the geochemical signatures are discussed. Moreover, the kinetic and biological effects that overprint the geochemical signatures suitable for paleoclimatic purposes, are assessed.

### 5.1. Fossil Preservation

The *Gigantoproductus* ventral valve can retain its original structural features at macro- and at microscale. The secondary and tertiary layers are well-defined by their microstructure, with a controlled preferred-crystallographic arrangement (the *c*-axis perpendicular to the shell edge). The microstructure is interpreted as unaltered because it retains its original morphological features, similar to those described in some extant brachiopods [82,83]. Likewise, submicrometric laminae have been recognized (Figure 3d) in columnar crystals of the tertiary layer, such as those described by Schmahl et al. [83] in the columnar crystals of *Terebratulina septentrionalis*. Furthermore, preserved nanocrystals, with a granular texture, were identified in some columnar microcrystals from natural breakage (Figure 3f,h). This finding suggests good preservation (i.e., the preservation of biogenic features) in fossil carbonate biominerals [29,84], because the nanocrystals are also characteristic of extant brachiopods [85].

Nonetheless, some areas show diagenetic alteration such as fractures, delamination, borings, micritization, and degrading neomorphism, with subsequent changes in the original crystallographic arrangement (Figure 4, Figure 5 and Figure S2).

The secondary and tertiary layers in *Gigantoproductus* shells are characterized by different microstructure and geochemical compositions, as was recognized in other extant and fossil brachiopods [36,43,86]. The secondary layer, in the *Gigantoproductus* of this study, is characterized by a laminar microstructure, with the elongate axis of crystals oriented parallel to the shell surface and with a small crystal size in comparison with the columnar microstructure of the tertiary layer, where the elongation and *c*-axis are oriented perpendicular to the shell surface and with larger crystals. Therefore, the morphological features of the secondary layer make it more prone to fracture, delamination, and recrystallisation. Within the tertiary layer, the thick region (Tk) of the valve is less affected by recrystallisation and fractures than the thin and umbonal regions (T, U). This may be due to their large crystal size and/or central shell position. On the other hand, the thin dorsal valve contains more fractures than the ventral valve, which makes it more susceptible to alteration and to more micritized patches (Table 4).

The secondary layer is highlighted by more intense luminescence under CL than the tertiary layer of the ventral valve (Figure 3). Similar differences in CL-luminescence intensity between the secondary and the tertiary layers had been recognized in *Composita subtilita* and *Neospirifer pattersoni* [87]. Although classically Mn and Fe are indicative of diagenetic alteration in calcite [88,89,90,91], not all luminescence, or its absence, can be correlated with diagenesis [65,78,79,92]. Biogenic carbonates can incorporate small amounts of Mn and Fe during their metabolic activity because of physicochemical variations of the environment in which they develop [92,93,94].

The luminescence differences in *Gigantoproductus* might be related to variations in the chemical composition; thus, the secondary layer is slightly enriched in Mn and Mg (Table 2), possibly related to more organic-matrix remains (Figure 3h). In addition, some fractures with a higher amount of Fe are non-luminescent under CL, and the growth lines with an apparently higher Mn concentration are luminescent under CL. This is partly due to the fact that Mn is a luminescence activator and Fe is an inhibitor of CL [91]. High Sr and Fe concentrations in some areas of the secondary layer suggest alteration by fluids favored by delaminated areas.

Slightly luminescent areas with a well-constrained crystallographic arrangement (considered here as well-preserved) have been identified in *Gigantoproductus*. These areas exhibit a luminescence pattern under CL that corresponds to the growth lines, and, in turn, show a slightly higher Mn content. This luminescence pattern under CL seems to be the result of organic matter and/or Mn [95] incorporated during shell growth. These areas also contain a higher Mg and S content, but no extensive Mn changes. The determination of small amounts of organic inclusions in growth lines (Figure 3h), and higher concentrations of Mg and Mn across the growth lines, suggest a combination of both sources, probably induced during the biocrystallization process. Similar luminescence patterns under CL were reported in the growth lines of the extant brachiopod *Megerlia truncata* [78], in fossil brachiopods [92,95] and in belemnite rostra [96]. Higher organic concentrations are recognized when the secretory regime of calcite is reduced [92]. This fact has been interpreted as growth cessation during environmental-stress events, produced by mantle anaerobiosis or by the acidification of calcification fluid by the closure of the valve [95,97,98]. Evaluation of these hypotheses deserves further study.

As described previously, not all non-luminescence areas of *Gigantoproductus* shells correspond to well-preserved zones. Crystals with disordered *c*-axis orientations have been identified in non-luminescent areas under petrographic microscopy. This may be due to slow recrystallisation processes, which keep the original microstructure but change the orientation of the *c*-axis. Examples of this process can be observed in coral skeletons [67,70]. Therefore, luminescent areas are not always indicative of diagenetic alteration [92] and non-luminescent areas are not always indicative of good preservation [29,37]. Banner and Kaufman [18] and Barbin and Gaspard [92] documented non-luminescent areas with altered ^87^Sr/^86^Sr and δ^18^O values in fossil brachiopod shells and slightly luminescent areas without evidence of alteration in an extant brachiopod.

The combination of CL and geochemical analyses helps to evaluate diagenetic alteration [65], but it is necessary to fully understand the original brachiopod-shell chemistry before using geochemical features such as a diagenetic indicator, in order to consider those biotic and abiotic factors that could control the original shell chemistry [36]. The chemical composition of the *Gigantoproductus* shells in this study is equivalent to that in the gigantoproductid reported by Popp et al. [17], Bruckschen et al. [20], Armendáriz et al. [9], Angiolini et al. [34,35] and Nolan [32]. The luminescence pattern under CL in well-preserved areas is similar to those described by Angiolini et al. [34,35]; additionally, a similar luminescence of diagenetically altered areas was described by Armendáriz et al. [9] and Nolan [32].

Minor and trace elements have been traditionally used to evaluate brachiopod-shell preservation by comparison between individuals and genera and with extant, unaltered brachiopod shells [17,28,36]. The NL and SL poorly preserved areas of the tertiary layer of the *Gigantoproductus* (Figure S3, Table 1) have ~800 ppm more Mg than the well-preserved areas. Moreover, the NL poorly preserved areas have ~800 ppm more Fe and ~120 ppm more Mn than the equivalent well-preserved areas. These values for the poorly preserved areas probably reflect the influence of Mg-, Fe-, and Mn-rich fluids during diagenesis in different stages (burial and meteoric waters). These data agree with the observations made by Grossman et al. [36] regarding altered brachiopod fossil shells, which are Fe- and Mn-enriched.

Additionally, the diagenetic alteration of valves modifies the isotopic record of δ^18^O, δ^13^C and ^87^Sr/^86^Sr. Poorly preserved areas exhibit δ^18^O and δ^13^C with larger standard deviations than those of the well-preserved areas (Table 3). Five samples of poorly preserved areas (Figure 8) are depleted in δ^18^O, with a similar depletion for δ^13^C; two samples are depleted in δ^18^O and δ^13^C; three samples are enriched in δ^18^O and δ^13^C; and two have similar amounts of δ^18^O, but show a δ^13^C enrichment with respect to the well-preserved areas.

In relation to Sr isotopes, well-preserved areas contain homogenous ^87^Sr/^86^Sr ratios, in contrast with poorly preserved areas (with one ^87^Sr/^86^Sr-enriched sample and one ^87^Sr/^86^Sr-depleted sample). Sr-isotope values from the *Gigantoproductus* (from 0.707830 to 0.707860) are equivalent to those shown by Bruckschen et al. (1999) in brachiopods from the Pendleian substage (mostly gigantoproductids shells, from 0.707828 to 0.707879), which validate the good preservation of the samples, except for two samples.

Different signatures of stable isotopes (δ^18^O and δ^13^C), trace elements (Mg, Mn, and Fe), and Sr isotopes suggest the co-existence of different diagenetic processes and stages acting on the *Gigantoproductus* shells, a fact which is also supported by the showcasing of microstructural and crystallographic changes.

On the other hand, the δ^18^O and δ^13^C trends in well-preserved areas can be explained by other mechanisms, such as kinetic and biological variations in ionic and isotopic values from the equilibrium of seawater (‘vital effects’).

### 5.2. Biological Overprint of Geochemical Signatures

‘Vital effects’ are geochemical deviations from the thermodynamic equilibrium of seawater produced by the vital processes of organisms, such as kinetic effects during ontogeny. Curry [99] observed three growth phases with different growth rates in *Terebratulina retusa*: a fast growth rate during the first stages of growth (in the umbonal region), followed by a constant growth period of three years and a progressively decreasing growth rate with brachiopod ageing. Variations in Mg across the shell have been widely studied in the literature as indicative of growth rates. Buening and Carlson [56] observed a higher Mg amount near the umbo of extant brachiopods, which decreases during brachiopod ageing.

The tertiary layer of the *Gigantoproductus* ventral valve can be divided into structural regions (umbonal, thick and thin regions) and growth stages (younger and older), because during ageing the shell grows in length (from umbo to commissure) and thickens (towards the interior of the valve), as a coupled process. Therefore, the U-, Tk- and T-regions must include younger (the outermost parts of the shell) and older (the innermost parts of the shell) areas, which may disguise some kinetic fractionation, considering the mean values of each area. For instance, structural regions exhibit differences in averaged Mg: the umbonal region has more Mg than the thick region (Tk), and the thin region (T) has the highest amount of Mg. On the other hand, transects analyzed for the function of distance exhibit a Mg depletion towards the interior from the outermost part of the ventral valve. Tk-region shows an exponential variation of Mg in contrast to T-region, which varies linearly.

Different Mg trends have been identified in extant brachiopod shells: an Mg-enrichment with ageing in the extant brachiopod shell *Magellania venosa* and a parabolic Mg trend with ageing in the extant brachiopod shells *Liothyrella neozelanica* and *Gryphus vitreus* [51]. Variations in Mg concentration of brachiopod shells seems to be species-specific [54,100], with different distribution trends among species [51] or within the same species [29,54]. Moreover, Rollion-Bard et al. [43] showed differences in the Mg/Ca incorporation across the shell in different extant brachiopod species, emphasizing the fact that Mg does not incorporate by a simple pathway during ontogeny. These differences may be related to different growth rates [56], crystallographic features [45], different proportions of organic components in the shell, seawater temperature and pH variations [42], and/or Mg exclusion from the calcification fluid [43]. Besides the Mg variation, S and Na vary from the outer edge of the ventral valve of *Gigantoproductus* towards the interior. The microstructure of the tertiary layer shows abundant smaller crystals located in the interphase between the secondary and tertiary layers (the initial biocrystallization event of the tertiary layer, Figure 2d), where nanograins and organic inclusions are frequent. Remarkably, these areas with small crystals are Mg- S- and Na-enriched, and during ageing the shell crystals enlarge and these elements decrease towards the interior.

Na in *Gigantoproductus* seems positively correlated with Mg and S (Figure 7) in the well-preserved areas. Na concentration in carbonates can be influenced by different processes that might explain this trend. For example, Na incorporation in abiogenic calcite and biogenic calcite may be favored by crystalline defects and by higher precipitation rates [101,102]. Moreover, Na can occupy an interstitial position in the carbonate-crystal lattice or can form part of the carbonate lattice by an altervalent substitution, creating a vacancy in the lattice [103,104]. On the other hand, Na can form part of the organic matter in biogenic carbonates; for instance, Rollion-Bard and Blamart [102] described a positive correlation between Na/Ca and Mg/Ca with the organic matter in bioaragonite in corals. Finally, a composition of carbonates can be strongly influenced by diagenesis, more than by environmental processes [105].

Mii et al. [106] showed a positive correlation between Mg, S and Na in fossil samples of *Neospirifer dunbari*, similar to the *Gigantoproductus* of this study, supporting the hypothesis that these trends are original signatures, indicative of well-preserved areas in fossils.

Mg, Na and S incorporation into a crystal lattice have been associated with crystal growth rates in crystal-growth experiments [101,107]. Moreover, growth rates associated with trace-element partitioning have been observed in both extant and fossil brachiopod shells [36,56,106]. Mii et al. [106] and Grossman et al. [36] related the high concentrations of these elements to seasonal growth rates, increasing during summer and decreasing during winter. Intra-shell small variations in Mg, Na and S in *Gigantoproductus*-shell transects (Figure 6) may be explained by this interpretation, despite the overprinted depletion trend from the shell edge towards the interior caused by brachiopod ageing.

All specimens of *Gigantoproductus* exhibit a depletion of δ^13^C and δ^18^O from older regions (the Tk- and T-regions) to younger regions (the U-region). It is remarkable that data from the thick region (Tk) are split into two clusters (Figure 8): one near the shell edge (younger), more depleted in δ^13^C, and another close to the shell interior (older), enriched in δ^13^C. Fractionation of δ^13^C and δ^18^O in *Gigantoproductus* shells exhibits an inverse trend to that observed in Mg, S and Na partitioning. Younger regions with higher growth rates are Mg-, S-, and Na-enriched and δ^13^C- and δ^18^O-depleted, whereas older zones are Mg-, S-, and Na-impoverished and δ^13^C- and δ^18^O-enriched. Preferential isotope fractionation has been previously described in extant and fossil brachiopods: for instance, Auclair et al. [49] described δ^13^C and δ^18^O depletion from the outermost (younger) to the innermost (older) part in the secondary layer of *Terebratalia transversa.* Similarly, Batt et al. [24] recognized δ^13^C depletion in the secondary layer of a fossil productid and δ^13^C and δ^18^O depletion in the tertiary layer from the outermost (younger) to the innermost (older) part of the genus *Composita* shell. Isotope fractionation was related to growth rate in brachiopod shells [24,49,50].

δ^13^C and δ^18^O direct- and inverse-covariations trends have been reported in extant and fossil brachiopod shells [24,42]. Moreover, the depletion trend of high growth-rate areas is not only linear, due to the parabolic trend, and inverse parabolic trends have been described in other brachiopod shells [51]. These variations in the partitioning and fractionation of ions and isotopes observed in different species and genera are called ‘vital effects’ and have been related to specific growth rates or metabolic changes in the brachiopods. Some authors were concerned about their influence on the shell geochemistry (e.g., Mg/Ca, δ^13^C and δ^18^O) and the possible paleoclimatic misinterpretations [50]. According to McConnaughey [108], ‘vital effects’ represent a noteworthy problem for isotopic geochemistry and paleoclimatology studies. The current results on *Gigantoproductus* geochemical variation underline the fact that they can be extended to the fossil record and, more specifically, to the interpretation of the Late Paleozoic Ice Age (LPIA). This requires the identification of ‘ideal’ sampling regions, in order to avoid differences in ion partitioning and isotope fractionation related to the differences in the growth rates (kinetic effects).

The *Gigantoproductus* regions less affected by “vital effects” (the *sensu* “equilibrium zone” or “plateau zone” identified by Perez-Huerta et al. [55] and Rollion-Bard et al. [43]) are the T-zone and the inner zone of the Tk-regions (Figure 7 and Figure 8), because of the low dispersion of the data. The umbonal region and external parts of the Tk-region of the *Gigantoproductus* ventral valves should be discarded, to avoid paleoclimatic misinterpretations (up to ~1.05‰ of δ^18^O and 14 mmol/mol of Mg/Ca). Herein, the *Gigantoproductus* tertiary layer offers great potential for paleoclimate studies, due to (i) a higher volume of shell substance compared with the secondary layer; (ii) growth-rate variability, which allows the characterization of the ‘vital effects’ of the shell; (iii) being less prone to diagenetic alteration than the secondary layer; and (iv) higher growth-line spacing, which allows for the avoidance of growth lines.

## 6. Conclusions

A comprehensive study of the microstructure, crystallography, and geochemistry (trace elements, δ^18^O, δ^13^C and ^87^Sr/^86^Sr) of the tertiary layer of the *Gigantoproductus* sp. ventral valves, has allowed the differentiation of areas with signs of diagenesis, from the primary biogenic features. In addition to fossil preservation, a special emphasis has been given to the characterization of the geochemical ‘vital effects’.

The *Gigantoproductus* shells consists of three layers, but only two are still preserved: the secondary layer with a laminar microstructure, and the tertiary layer with a characteristic columnar microstructure.

Evidence of diagenesis has been recognized in some poorly preserved areas, because the original crystallo-chemical features have been obliterated. These areas, regardless of luminescence under CL (i.e., NL or SL), show neomorphism, a random crystallographic distribution, and a disperse geochemical composition. It should be highlighted that the luminescence under fluorescence microscopy, identified in some well-preserved areas as growth lines, corresponds mainly to preserved organic-matrix remains.

Well-preserved areas of secondary and tertiary layers exhibit a well-constrained crystallographic arrangement, with the *c*-axis oriented parallel to the elongation axis of the prisms and perpendicular to the laminae. Three structural regions were identified in the ventral valve, based on the curvature and thickness changes in the ventral valve: the umbonal (U), thick (Tk) and thin (T) regions.

Furthermore, the geochemical composition of these areas is well-constrained in MTE (Ca, Mg, Sr, Na, S, Mn, and Fe) as well as in isotope composition (δ^13^C, δ^18^O and ^87^Sr/^86^Sr), with a characteristic variability possibly related to shell growth-rate differences, these being identified as geochemical ‘vital effects’. Younger regions with higher growth rates (e.g., umbonal and the secondary—tertiary-layer interphase) are Mg-, S- and Na-enriched and δ^13^C- and δ^18^O-depleted, whereas older zones (the inner zones of the Tk- and T-regions) are Mg-, S- and Na-impoverished and δ^13^C- and δ^18^O-enriched. These latter regions are the most suitable areas for geochemical analysis of the *Gigantoproductus*.

The ‘vital effects’ caused by kinetic effects have a direct influence on the paleotemperature estimation; up until ~1.05‰ of δ^18^O and 14 mmol/mol of Mg/Ca, intra-shell variation was detected, which can modify the ulterior palaeoclimatological interpretation, underestimating or overestimating the environmental conditions of the LPIA.

## Figures and Tables

**Table 1:** Descriptive statistics of major, minor and trace-element (MTE) composition of five preservation states identified. Values in ppm.

Preservation State	Element	N total	Mean	S.D.	Minimum	Median	Maximum
WP-NL	Ca	205	390,449.9	6880.8	372,632.4	391,045.6	406,978.5
	Mg		3380.5	911.3	1218.3	3238.6	5886.3
	Sr		1737.9	430.5	642.7	1767.3	2968.1
	Mn		155.3	214.3	0.0	0.0	836.6
	Fe		184.3	263.0	0.0	0.0	1026.2
	S		1060.2	328.8	388.5	993.2	1894.4
	Na		1038.2	358.4	0.0	1031.0	2202.8
PP-NL	Ca	20	372,422.6	29,616.5	269,479.6	380,938.4	400,052.1
	Mg		4651.7	1876.9	645.3	5020.8	7273.4
	Sr		1763.1	473.2	465.1	1839.2	2443.8
	Mn		278.9	467.2	0.0	27.1	1828.1
	Fe		955.4	1710.0	0.0	427.6	6499.1
	S		1490.5	929.9	0.0	1620.0	3576.5
	Na		926.0	574.8	0.0	997.6	2017.4
WP-SL	Ca	44	382,121.0	8596.2	365,870.4	382,139.2	398,808.4
	Mg		4164.8	1044.6	2291.8	3959.4	6748.7
	Sr		2208.9	444.7	1166.9	2262.0	3027.2
	Mn		314.9	367.3	0.0	170.4	1332.3
	Fe		371.2	408.2	0.0	268.2	1391.5
	S		994.7	373.6	460.6	979.2	1974.5
	Na		932.9	358.0	311.5	867.8	1713.3
PP-SL	Ca	80	378,873.3	141,25.2	313,975.9	383,615.3	398,608.2
	Mg		4378.5	1468.8	1724.9	4010.6	9661.7
	Sr		1975.1	501.0	879.4	1894.1	3729.1
	Mn		125.0	220.3	0.0	0.0	1038.0
	Fe		451.7	880.7	0.0	139.9	6499.1
	S		1202.5	565.0	4.0	1125.4	3576.5
	Na		920.4	378.3	29.7	934.5	2010.0
PP-L	Ca	15	382,641.0	8793.2	358,236.3	383,947.7	394,598.2
	Mg		4829.2	1635.3	2249.6	4589.6	7786.0
	Sr		1981.5	473.8	1217.7	2232.4	2765.1
	Mn		582.0	292.4	302.1	457.0	1332.3
	Fe		56.0	89.6	0.0	0.0	287.6
	S		1264.5	462.3	596.7	1093.4	1918.4
	Na		966.7	276.5	504.4	1023.5	1550.2

WP-NL: well-preserved, non-luminescent; PP-NL: poorly preserved non-luminescent; WP-SL: well-preserved, slightly luminescent; PP-SL: poorly preserved, slightly luminescent; PP-L: poorly preserved, luminescent; S.D. Standard deviation.

**Table 2:** Descriptive statistics of major, minor and trace-element (MTE) composition of tertiary-layer shell regions and secondary layer. Values in ppm.

Shell Part	Element	N Total	Mean	S.D.	Minimum	Median	Maximum
U	Ca	33	388,430.1	6175.9	373,604.5	389,194.3	398,379.5
	Mg		3259.7	840.4	1899.8	3178.3	5735.5
	Sr		1604.6	551.8	642.7	1648.9	2570.6
	Mn		227.5	255.8	0	170.4	836.6
	Fe		205	266.8	0	38.9	1026.2
	S		930.1	303.3	440.6	893.1	1786.2
	Na		1131.9	337.6	526.6	1105.1	1995.2
Tk	Ca	97	393,264.2	6774.8	372,632.4	394,433.8	406,978.5
	Mg		3012.1	722.7	1218.3	2943.1	4999.7
	Sr		1656.4	408.8	845.6	1623.6	2460.7
	Mn		140.9	202.1	0	0	774.6
	Fe		219.4	282.1	0	46.6	925.1
	S		1040.1	371.3	408.5	957.2	2367
	Na		1063.5	401.9	0	1068	2202.8
T	Ca	90	386,561.5	7115.3	366,270.7	387,503.8	397,164.3
	Mg		4126.5	1125.4	2056.6	4067.9	7273.4
	Sr		1890.7	330.4	1107.7	1902.6	2968.1
	Mn		160.1	244.8	0	0	1332.3
	Fe		161.4	264.3	0	0	1041.7
	S		1249.2	470.2	224.3	1165.5	3576.5
	Na		968.8	346.5	0	945.7	1579.8
Secondary layer	Ca	30	379,749.4	8141.1	365,870.4	378,940.5	398,808.4
	Mg		4444.0	1076.2	2780.3	4565.5	6748.7
	Sr		2283.4	360.2	1462.9	2367.7	3027.2
	Mn		182.3	210.1	0.0	116.2	704.9
	Fe		370.0	453.7	0.0	225.4	1609.2
	S		980.8	288.5	460.6	1039.3	1549.9
	Na		1013.9	385.7	311.5	997.5	1713.3

U: umbonal region; Tk: thick region; T: thin region; S.D. Standard deviation.

**Table 3:** Isotopic composition of the structural regions identified in the tertiary layer of *Gigantoproductus* shells and poorly preserved areas. Bold highlights the mean of each structural part.

Shell Region	δ^18^O	δ^13^C	^87^Sr/^86^Sr (Std Err)	M.S.D.	Shell Region	δ^18^O	δ^13^C	^87^Sr/^86^Sr (Std Err)	M.S.D.	Shell Region	δ^18^O	δ^13^C	^87^Sr/^86^Sr (Std Err)	M.S.D.
Umbornal	−3.4	2.78			Thin	−2.91	3.35	0.70786						
	−3.61	2.35				−2.77	3.41	(4^−6^)		Poorly preserved	−3.71	1.84		
	−3.78	2.39				−3.13	3.29	0.707845			−2.86	4.27		
	−3.55	2.68				−2.99	2.95	(4^−6^)			−5.06	3.62	0.707893	
	−3.25	2.52				−2.93	3.15				−4.12	3.68	(4^−6^)	
	−3.14	2.97				−2.33	3.04				−2.32	3.87		
	−3.27	2.81				−2.44	3.17				−6.28	3.08		
**Mean**	**−3.43**	**2.64**		**0.22/0.23**		−2.34	3.38				−4.37	1.18		
Thick	−2.82	2.18	0.70783			−2.54	3.29				−1.64	3.05		
	−3.39	2.1	(4^−6^)			−2.27	2.89				−1.89	3.76		
	−2.62	2.25			**Mean**	**−2.67**	**3.19**		**0.32/0.18**		−3.87	3.3	0.707807	
	−2.92	2.29			Dorsal Valve	−3.25	2.49				−2.16	2.61	(5^−6^)	
	−2	3.09				−4.02	1.54				−2.14	2.43		
	−2.58	3.04				−1.46	3.56				−1.83	3.46		
	−2.33	2.96	0.707831			−3.13	2.13				−4.03	3.34		
	−2.32	2.94	(4^−6^)		**Mean**	**−2.97**	**2.43**		**1.08/0.85**	**Mean**	**−3.31**	**3.11**		**1.4/0.84**
	−2.71	2.86												
**Mean**	**−2.63**	**2.63**		**0.40/0.42**										

M.S.D. Mean standard deviation; Std Err: Standard deviation error.

**Table 4:** Summary of structural and geochemical features of the five states of preservation identified.

Features		Preservation States						
		Well-Preserved			Poorly Preserved			
		Tertiary Layer		Secondary Layer	Tertiary Layer	Growth Lines	Dorsal Valve	Secondary Layer
Structural	Microstructure	Prismatic crystals with noticeable microlaminae, preferential orientation	Growth lines: original organic-matrix remains and preferential orientation of the crystals	Lath crystals, original organic-matrix remains and preferential orientation	Recrystallisation (e.g., degrading neomorphism), sparite cements, filled fractures, small patches of crystals without a preferential orientation	Recrystallisation, delamination	Recrystallisation, matrix infilling, micritzation, sparite cements, filled fractures	Recrystallisation, delamination, micritized patches, matrix infilling, filled fractures
	Cathodoluminescence	Non-luminescent	Slightly luminescent, luminescence pattern in growth lines	Slightly luminescent, luminescence banding	Non-luminescent	Increasing luminescence near fractured areas	Luminescent	Slightly luminescent
Geochemical	Crystallographic	Highly arranged crystals: *c*-axis oriented perpendicular to shell growth	Highly arranged crystals: *c*-axis oriented subparallel to shell growth	Highly arranged crystals: *c*-axis oriented parallel to shell growth	Poorly arranged crystals: *c*-axis randomly oriented			
	MTE (ppm)	Mg: 3380, Sr: 1737, Mn: 155, Fe: 184	Mg: 4164, Sr: 2208, Mn: 314, Fe: 371	Mg: 4444 Sr: 2283 Mn: 182 Fe: 370	Mg: 4651, Sr: 1763, Mn: 278, Fe: 955	Mg: 3765, Sr: 1922, Mn: 971, Fe: 858	_	Mg: 4849, Sr: 2323, Mn: 117, Fe: 838
	δ^13^C–δ^18^O	Low dispersion of the ratio δ^18^O: −3.78‰ to −2‰; δ^13^C: 2.09‰ to 3.4‰; grouped in clusters depending on ontogeny development			High dispersion of the ratio δ^18^O: −6.28‰ to −1.83‰; δ^13^C: 1.18‰ to 4.27‰			
	^87^Sr/^86^Sr	Low dispersion of the ratio: 0.707830–0.707860			High dispersion of the ratio: 0.707807–0.707893			

## Data Availability

All data are included in figures and tables of this article.

## Supplementary Materials

The following supporting information can be downloaded at https://www.mdpi.com/article/10.3390/life13030714/s1, Figure S1: Morphological characters and figure of *Gigantoproductus* sp.; Figure S2: CIP analysis of thin region, umbonal region, and thick region of *Gigantoproductus* shells. Figure S3: Boxplot of the seven analyzed elements in well-preserved and poorly-preserved areas; Figure S4: All drill locations sampled in the specimens over a brachiopod scheme to compare its relative position into the ventral valve; Table S1: Major, minor and trace elements data in ppm from *Gigantoproductus* sp.
