# Supplementary material for: Diagenetic and Biological Overprints in Geochemical Signatures of the Gigantoproductus Tertiary Layer (Brachiopoda): Assessing the Paleoclimatic Interpretation
Source: Life (Basel). 2023 Mar 6;13(3):714. doi: 10.3390/life13030714 (PMC10057505; doi:10.3390/life13030714)
Supplement: Supplementary file 1 [file life-13-00714-s001.zip › Mateos-Carralafuente et al._text.pdf]

life

MDPI

Article

Diagenetic and biological overprints in geochemical signatures of *Gigantoproductus* tertiary layer (Brachiopoda): assessing the paleoclimatological interpretation

J. Ricardo Mateos-Carralafuente[1], Ismael Coronado*[,2], Juncal A. Cruz[2], Pedro Cózar[3], Esperanza Fernández-Martínez[2], Sergio Rodríguez[1,3]

[1] Department of Geodynamics, Stratigraphy and Paleontology, Faculty of Geological Sciences, Complutense University of Madrid, c/ José Antonio Novais, 12, 28040, Madrid (Spain): josericm@ucm.es; sergrodr@ucm.es
[2] Faculty of Biological and Environmental Sciences, University of Leon, Campus Vegazana s/n, 24071 León, Spain: icorv@unileon.es; jcrum@unileon.es.
[3] Instituto de Geociencias (CSIC, UCM), c/ Severo Ochoa 7, 28040-Madrid (Spain): pcozar@ucm.es.

* Correspondence: IC: icorv@unileon.es; Tel.: (34) 987293644; and J.R. M-C.

Simple Summary: The climate of the past is inaccessible using recent meteorological instruments, therefore scientists use proxies to get that information. One of these proxies is the chemical composition of shells and skeletons of marine animals. To understand the sea-temperature of Carboniferous (around 359 Ma) scientists retrieve isotopic information of brachiopod shells but the biological dynamics of the organism, such as growth rate, nutrients availability… unbalances the isotopic ratios, complicating the paleoclimatological reconstruction. This process is named 'vital effect'. The study analyses the largest brachiopods ever (*Gigantoproductus*) for two reasons: 1) their thick and large shell is easily sampled; 2) they lived around 347 to 323 Ma, when one of the most severe ice ages took place. These animals recorded in their shells valuable isotopic and elemental information but to create feasible paleoclimatological reconstruction the vital effects should be identified. This study deepens into the shell, from a multidisciplinary approach, searching mineralogical, chemical and crystallographic information that helps to identify them. The study revealed that isotopic ratios of *Gigantoproductus* shells change, because of the growth rate, recording differences up to 5°C in the sea-temperature at different part of the shell.

Abstract: Variations in geochemical signatures of fossil brachiopod shells may be due to diagenesis and/or biological processes (*i.e.* ‘vital effects’). Characterize them is crucial to identify reliable areas into the shell suitable to paleoclimatological studies. This investigation contributes to an in-depth understanding of geochemical variations of *Gigantoproductus* sp. shells (SW Spain, Serpukhovian age), which could affect to the Late Paleozoic Ice Age interpretation. Microstructural, crystallographic, cathodoluminescence and geochemical (minor and trace elements, $\delta^{18}O$, $\delta^{13}C$, and strontium isotopes) characterisation have been performed on the tertiary layer of the ventral valve, to assess the preservation state. Poorly-preserved areas exhibit microstructural and geochemical changes such as recrystallisation, fracturing and higher Mn and Fe enrichment. Moreover, these areas have higher dispersion of ^{86}Sr, ^{87}Sr, $\delta^{18}O$ and $\delta^{13}C$ than well-preserved areas. Three structural regions have been identified in well-preserved areas of ventral valve by differences in valve curvature and thickness, such as the umbonal, thick and thin regions. These regions have different proportions of Mg, S, Na, $\delta^{18}O$, and $\delta^{13}C$, whom are interpreted as ‘vital effects’ and probably are related with growth rate differences during shell growth. *Gigantoproductus* tertiary layer seems the best suitable to paleoclimatological studies because it retains the original microstructure and geochemistry.

Keywords: Carboniferous, microstructure, trace elements partitioning, Serpukhovian, stable isotope fractionation.

Citation: To be added by editorial staff during production.

Academic Editor: Firstname Lastname

Received: date
Revised: date
Accepted: date
Published: date

Copyright: © 2023 by the authors. Submitted for possible open access publication under the terms and conditions of the Creative Commons Attribution (CC BY) license (https://creativecommons.org/licenses/by/4.0/).

1. Introduction

The Late Paleozoic Ice Age (LPIA), elapsed from Viséan to Capitanian (~335 to 260 Ma) [1], was a dynamic climatic phenomenon that involved multiple glacial and interglacial events driven by continental Pangea conformation, and low global atmospheric carbon dioxide concentrations (pCO_2). These constraints, in turn, produced glacioeustatic variations, changes in ocean circulation and finally, turnover of marine flora and faunas [1–8]. Mid-Carboniferous glacial peak represented an important change in the seawater chemistry and ocean circulation [9] and one of the most important and selective crises in the Phanerozoic Era [10]. Low speciation and high extinction rates of marine faunas is an outstanding feature during this period, particularly the late Serpukhovian [11].

Most of the paleoclimatological Carboniferous data are based on carbonate brachiopod $\delta^{18}O$ and $\delta^{13}C$ signatures, as paleotemperature and carbon cycle proxies respectively [9,12–24]. Rhynchonellid brachiopod shells are commonly selected for Carboniferous paleoclimate reconstructions due to their worldwide distribution, abundance [13,19–21,25–28]. Carbonate brachiopod shells are composed of low magnesium calcite (LMC), a relatively resistant polymorph to diagenesis [29].

Gigantoproductids (tribe Gigantoproductini [30]) are key due to its great potential for paleoclimatological studies because: i) frequent during Carboniferous [28,31,32]; ii) global distribution [30]; iii) large shell thickness and size [33], which allow the extraction of material for geochemical analyses easier than other fossil brachiopods [28,34,35]. Large shell size has been attributed to a possible a chemosymbiotic lifestyle [35]. In spite of these advantages, few studies have been focused on this fossil group for paleoclimatological purposes [9,17,22,28,34]. These studies reported geochemical variations in gigantoproductid shells regarding other brachiopod genera, for example higher contents of Mg, S and Na than *Martinia* sp. and *Chorisitites* sp. [17,28]. Moreover, large fluctuations in stable isotopes ($\delta^{18}O$ and $\delta^{13}C$) have been described in gigantoproductid shells concerning other brachiopod genera, like *Chorisitites* sp., of the same age and localities [28]. These variable compositions detected in gigantoproductid shells raise several hypotheses on their own origin: i) they could be result of diagenesis; ii) they could be caused by kinetic and biological effects during shell growth; iii) or a combination of both processes.

Despite the relatively resistance of the brachiopod shells to diagenesis, this may obliterate the original microstructure and modify the shells geochemistry, making difficult the paleoclimatological interpretation. In order to recognize diagenetic alteration, the identification of non-altered and altered areas into brachiopod shell is needed. Altered regions are usually enriched or depleted in some trace elements such as Mn, Fe, Sr, and Na [36]; exhibit isotope outliers; show luminescence under cathodoluminescence [17], although non-luminescence is not always indicative of good preservation [37]; lack of microstructures [36]; and loss of the crystallographic orientation of biomineral structures [38].

Despite the outstanding role of brachiopods shells in paleoclimatological studies some researchers have identified 'vital effects', considered herein as significant geochemical deviations from thermodynamic equilibrium of seawater [39]. These variations have been recognized in extant brachiopods [40–43] and in well-preserved areas of fossil brachiopods (*e.g.*, [22,24,34]). These geochemical variations in biominerals can be ascribed to: i) biological processes [44], those related to the metabolism and physiology of the organism (named 'true vital effects' by Pérez-Huerta and Andrus [45]); and ii) bio-crystallisation processes, those related to mechanisms of non-classical precipitation of biominerals [46,47]; and in addition both categories might be also affected by environmental factors such as temperature, salinity, ocean acidification [46,48]. The selection between these hypotheses is complicated, and even, an unaffordable process due to the 'vital effects', which currently, is a poorly known mechanism [45]. Geochemical variations derived from

brachiopods shell growth are included within the category of biological processes, such as kinetic effects or metabolic effects, including the metabolic prioritisation [24,40,42,49].

Growth rate and stable isotopic fractionation covariation have been identified in $\delta^{18}O$ and $\delta^{13}C$ in extant and fossil brachiopods [24,35,40,49–52]. $\delta^{18}O$ and $\delta^{13}C$ variations between shell layers have been documented in extant and fossil brachiopods, which were considered as kinetic effects of the shell growth [24,43,49–51]. The tertiary layer is often selected for paleoclimatological studies due to the slower growth rate than the primary and secondary layers [9,28,34,36,43].

On the other hand, tailored $\delta^{26}Mg$ fractionation has been detected between the primary and secondary layer in extant brachiopods [42]. Shell regions with higher growth rates are usually characterized by ^{26}Mg-enriched and ^{13}C- and ^{18}O-depleted [42]. Therefore, a covariation between growth rates and ion partitioning (*e.g.*, minor and trace elements, MTE, such as Mg, Na, and S) has been identified in extant brachiopods [42], who described a heterogeneous Mg intra-shell distribution. Grossman et al. [36] described a covariation between Na and S from inner to outer fossil brachiopod shells related to growth rates, with higher Na and S contents during higher growth rates. Moreover, a wide-ranging Mg variation in extant brachiopod shells has been documented [53–55]. The amount of $MgCO_3$ in brachiopod shells varies ontogenetically during growth from Mg-enriched younger zones to progressive depleted older zones [55–58]. The Mg variation has a direct influence on the Mg/Ca ratio for the paleotemperature calculation and needs further studies to establish a robust knowledge of brachiopod growth rate and element covariation. This background highlights the need for a detailed characterisation of the original microstructure and geochemistry of fossil brachiopod shells prior to assess the geochemical variation produced by diagenetic alteration and 'vital effects' [29,36].

The main aims of this study are: i) to characterize the biogenic and diagenetic features of the tertiary layer of *Gigantoproductus* sp. ventral valves (SW Spain, Serpukhovian age), using a combination of structural, geochemical, and crystallographic techniques; ii) to establish the most favourable zones for geochemical sampling, avoiding paleoclimatological misinterpretations.

2. Geological setting

The studied specimens have been collected in the Guadiato Valley, coordinates 5º8′20′′W - 38º14′N, [59], a NW-SE elongated Carboniferous outcrop included into Sierra Morena, in the southern Iberian Massif (Figure 1). Gigantoproductid samples were collected from the San Antonio section (San Antonio-La Juliana Unit), assigned to the Pendleian, Serpukhovian [60,61].

The Guadiato Valley (Figure 1) is an elongated zone where Carboniferous outcrops (late Viséan to early Westphalian) are separated by WNW-ESE faults [61]. It is divided into three tectono-sedimentary marine units: the Fresnedoso Unit, formed by siliciclastic rocks of Viséan age; the Sierra del Castillo Unit formed mainly by carbonates of Viséan age and the San Antonio-La Juliana Unit, formed by siliciclastics and carbonates of Serpukhovian age [59].

The San Antonio-La Juliana Unit has been interpreted in origin as a strike-slip basin, syntectonic, formed, by terrigenous and carbonate sediments of marine origin, which belong to several geographically close sedimentary environments. It comprises slope facies (hemipelagic sediments, olistoliths, debris flows and turbidites), platform facies (internal platform, tempestites with shallowing episodes), tidal plain facies (intertidal plain and small lakes) and deltaic facies [60,62].

The San Antonio section, 146 m thick, is formed mainly by shales and siltstones with intercalated limestones and calcareous marlstones (Figure 1). The succession is open marine with slope facies at the base that evolve to platform facies and deltaic facies in the upper part. The paleontological content is mostly represented by large-sized crinoids, gigantoproductids, bryozoans, rugose and tabulate corals, cyanoliths [62,63] and conodonts

[61]. Based on foraminifera and conodonts the age is Pendleian (Early Serpukhovian; [61,64]).

2. Materials and Methods

2.1. Studied material

Eleven specimens of *Gigantoproductus* sp. (Figure S1) have been selected for this study, from the 2-4 horizon of San Antonio section (Figure 1). The material (DMP-A301-1014-1 to DMP-A301-1014-11) is housed in the Paleontological Collections of the Department of Paleontology (Complutense University of Madrid, UCM). The specimens were sectioned in two halves longitudinally, by the sagittal plane from the umbo to the commissure, when possible, and each slab was polished using sandpaper and 1 μm and 0.3 μm alumina. Polished samples were scanned in order to cross-reference digitally the macroscopic features of shells. One slab of each of the 11 specimens has been used for this study, the other 11 slabs have been reserved for future studies. A total of twenty thin-sections and five ultra-thin sections were prepared (*sensu* Coronado et al. [65]).

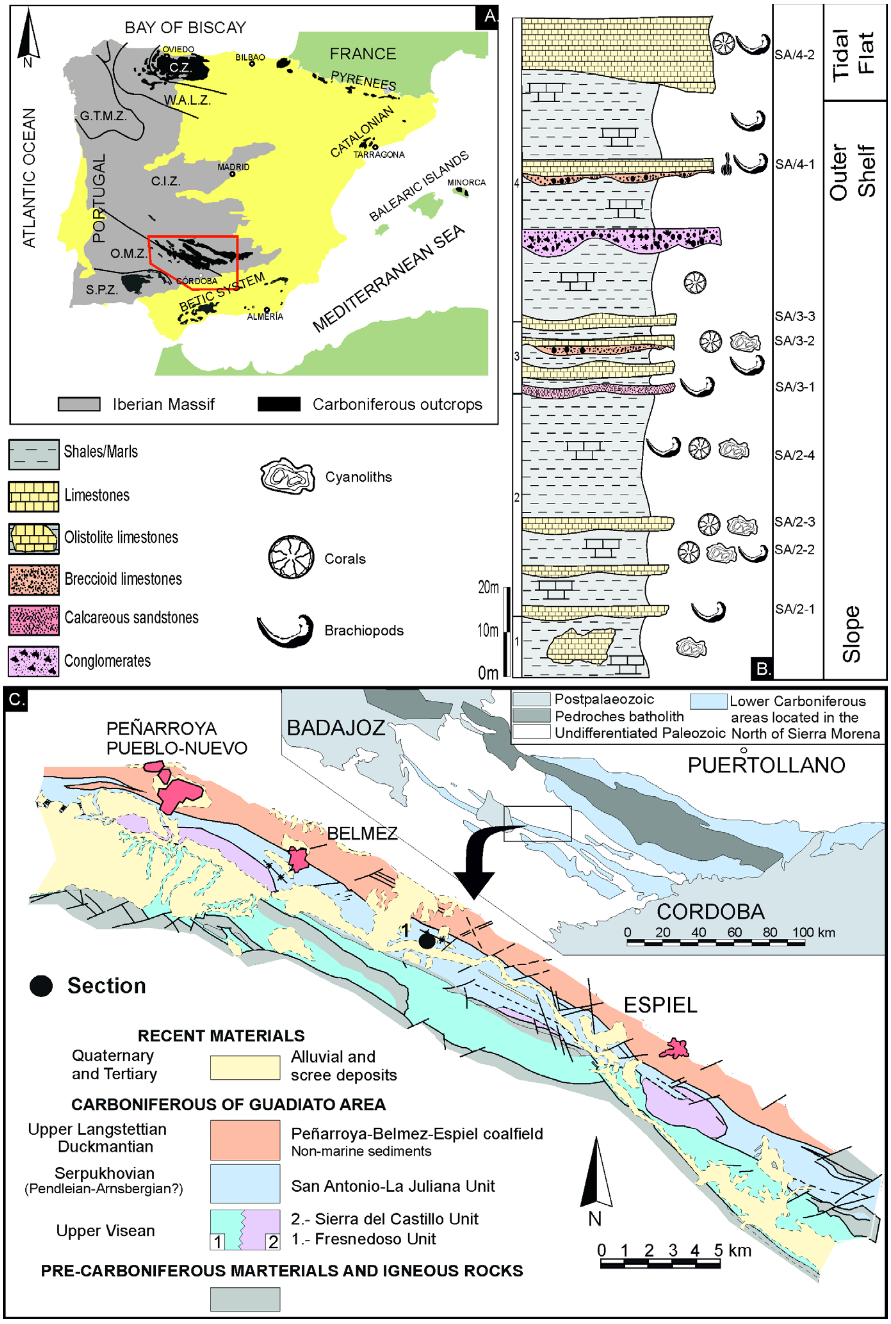

Figure 1. a) Synthetic map showing the distribution of the main Carboniferous outcrops of the Iberian Peninsula, modified from Colmenero et al. (2002). b) Stratigraphic log of the San Antonio section with geographic coordinates 5º8'20''W - 38º14'N, modified from Cózar (1998) and Cózar et al. (2003). c) Early Carboniferous outcrops (upper Tournaisian to Arnsbergian) in Sierra Morena (upper part) and synthetic map with the main geological units of the Guadiato Area (lower part). Modified from Cózar 1998). Cantabrian Zone, C.Z.; West-Asturian-Leonese Zone, W.A.L.Z.; Galicia-Tras os Montes, G.T.M.Z.; Central-Iberian Zone, C.I.Z.; Ossa-Morena Zone, O. M. Z. and South Portuguese Zone, S.P.Z.).

2.2. *Microscopy methods*

The specimens have been studied under petrographic microscope, scanning electron microscopy (SEM), computer-integrated polarisation (CIP) and cathodoluminescence microscopy (CL) in order to select well-preserved areas for subsequent drilling.

A petrographic microscope LM Leica DMLP with coupled camera Leica DC 300 has been used with the purpose of characterizing the microstructure of brachiopod shell.

Samples have been extracted by natural breakage from ventral valves of *Gigantoproductus* sp. between the muscle scars and the commissure, perpendicular and parallel to the valve growth. Moreover, a complete shell section has been polished and etched in a 5% HCL solution for 20-25 seconds. These samples have been coated with gold and analysed by scanning electron microscopy (SEM), using a model JEOL JSM-820 working at 20 kV, located in the research facility of Geological Techniques of Complutense University of Madrid (Spain), in order to complete the microstructural characterisation.

Thin-sections have been photographed under CL working at 16 kV and a current of 0.5 mA, in order to assess the intensity and distribution of luminescence. A cold cathodoluminescence probe model 8200 MK4 of Cambridge Image Technology Ltd attached to a petrographic microscope model Eclipse E400 POL with a camera, has been used for such purpose. This microscope is located in the Geological Storage Division, Hydro-geochemical Group Ciemat (Madrid, Spain).

The crystallographic organisation of brachiopod shells has been studied using computer-integrated-polarisation microscopy (CIP, Heilbronner and Barrett 2014), in order to evaluate the shell preservation. This method for texture analysis and optical orientation imaging has been applied in biomineralisation studies of fossil bio-calcite with relevant crystallographic and structural results, highlighting as a robust tool in diagenetic characterisation [66–68]. This method determines the *c*-axis orientations of uniaxial minerals (as calcite and quartz) from optical micrographs, displaying the results in pole figures and orientation images, using an RGB colour-code, which represents each orientation.

Seven CIP analyses in different samples and regions of the shell were performed using an experimental petrographic Zeiss microscope with an automated rotation and tilting system, confectioned with the Arduino UNO microcontroller (open-source hardware) and controlled with a software implemented in LabVIEW environment (details in Coronado and Rodríguez [69]). The lower ratio pixel–µm reached with this setting in the micrographs is 1:0.05. Finally, the micrographs were processed using Image SXM software[70].

Crystal sizes were measured via micrographs using the plug-in ObjectJ 1.03w [71] of the free and open-source ImageJ 1.47v image processing software [72].

2.2. Trace elements and stable isotope analyses

Seven elements (Ca, Mg, Sr, S, Na, Mn and Fe) have been analysed with electron microprobe analysis (EMPA) conducted on eleven thin-sections (364 punctual analyses, 20 or 25 points per thin-section), using a JEOL Superprobe JXA-8900M with five wavelength-dispersive spectrometers located in the Spanish National Centre for Electron Microscopy of the UCM. Each point has been analysed with an accelerating voltage of 15 kV, a beam current of 10 nA and a spot size of 5 µm. Each analysis takes an acquisition time of 45 seconds; because of five elements can be analysed simultaneously, the total time for analysis of the seven elements has been 90 seconds. The following standards and detection limits have been used in EMPA: Ca: 201.5 ppm, Mg: 181.25 ppm (dolomite); Fe: 418.5 ppm, Mn: 439.5 ppm (siderite); Sr: 262.25 ppm (strontianite); Na: 167.75 ppm (albite) and S: 235.25 ppm (galena). In addition, the same seven elements were mapped. The EMPA mapping enables simultaneous analysis of different elements and the generation of distribution images for each element with 1 µm resolution. An accelerating voltage of 20 kV with a beam current of 100 nA and a spot size and step interval of 1 µm diameter (dwell time = 25 ms) were used.

Samples were powdered using a microdrill with an x-y micrometric plate attached to a binocular and a 500 µm tungsten carbide drill in selected areas, sampling for stable isotope analysis ($\delta^{18}O$, $\delta^{13}C$) and $^{87}Sr/^{86}Sr$ measurements. Stable isotope sampling requires only one drill hole to obtain enough material whereas $^{87}Sr/^{86}Sr$ requires 30 to 40 drill holes.

Thermal Ionisation Mass Spectrometry (TIMS) has been used in order to date the samples of study by the $^{87}Sr/^{86}Sr$ ratio. 7 mg were obtained by drilling of 4 samples of well-preserved zones and 2 samples of poorly-preserved zones. The material was processed to obtain a Sr concentrate residue, which was loaded onto a Re filament by adding 1 µl of

H_3PO_4 1M 4 and 2 μl of Ta_2O_5. The Sr isotopic ratios have been analysed on a TIMS-Sector 54® Mass Spectrometer, located in the Laboratory of Geochronology and Isotope Geochemistry (UCM), following a dynamic multicollection data acquisition method for 10 blocks of 16 cycles each with beam intensity mass of ^{88}Sr at 3V. Sr analyses have been corrected to avoid possible interferences of ^{87}Rb. The $^{87}Sr/^{86}Sr$ ratios have been normalized with respect to the measured value of the ratio $^{86}Sr/^{88}Sr = 0.1194$, in order to correct the possible fractionation of masses that the sample might have undergone during the filament loading and/or instrumental analysis. During the analysis of the samples, the isotopic standard of Sr (NBS 987) has been analysed repeatedly and the following values have been obtained: $0.710239 \pm 1.7 \cdot 10^{-5}$ (n = 8). These values have been used to correct, taking into consideration the plausible drift referred to the standard, and the certified value of the standard.

Stable isotope analyses ($\delta^{18}O$ and $\delta^{13}C$) were undertaken using a triple collector isotope ratio mass spectrometer, Finnigan MAT 253 of the Stable Isotope Laboratory of the Department of Geological Sciences of the University of Michigan (USA). 44 samples have been drilled, obtaining 0.1 mg of each, 30 from well-preserved zones and 14 from zones with evidence of poorly preservation, previously characterized in optical microscopy, cathodoluminescence and CIP, weighted and digested using H_3PO_4 at 77° ± 1°C for 8 minutes in a Finnigan MAT Kiel IV. Have been calibrated for a better-fit regression line defined by two international standards NBS 18 (National Bureau of Standards; $\delta^{13}C$= -5.014‰ and $\delta^{18}O$= -23.2‰) and 19 (National Bureau of Standards; $\delta^{13}C$=1.95‰ and $\delta^{18}O$=-2.20‰). The data are given in ‰ notation relative to the VPDB (Vienna Pee Dee Belemnite). The accuracy of the data has been monitored through daily analyses with a variety of carbonate powder standards. At least 4 carbonate standards have been reacted, analysed and measured with accuracy below ±0.1‰ for $\delta^{13}C$ and $\delta^{18}O$.

3. Results

Collected specimens from San Antonio section belong to horizons 2-4, which make unsuitable for correlating geochemical variations with environmental factors (*e.g.*, paleoseasonality). Results have been divided into three main sections: shell microstructure, fossil preservation and geochemical characterisation, to differentiate diagenetic and biological overprints in *Gigantoproductus* shells.

3.1. Shell microstructure

Gigantoproductids have a concave-convex shell conformed by a dorsal and ventral valve. The dorsal valve is thinner than the ventral valve. It has mostly laminar microstructure, with lath crystals (*sensu* [73]), ~1-2 μm width, and they are grouped in laminae (*sensu* [34,73,74]) ~10 to 20 μm thick (Figure 2a), a few columnar layers with smaller crystals than the ventral valve appear between the laminar microstructure. The crystal size decreases towards the inner part of shell as it was recognized in the ventral valve.

The dorsal valve (Figure 2a) shows similar thickness (~2-3 mm), which decreases towards the commissure, whereas ventral valve has a noticeable thickness difference. These differences conform the shell shape, which can be divided in three regions of ventral valve, the umbonal region (U-region), more incurved than the rest of the shell and with ~8-10 mm in thickness, the thick region (Tk-region), is a thickened area, ~13-22 mm, following the umbonal region and the thin region (T-region) more planar than the umbonal and the thick region, with ~5-7 mm thickness which decreases from the thick region to the commissure (Figure 2).

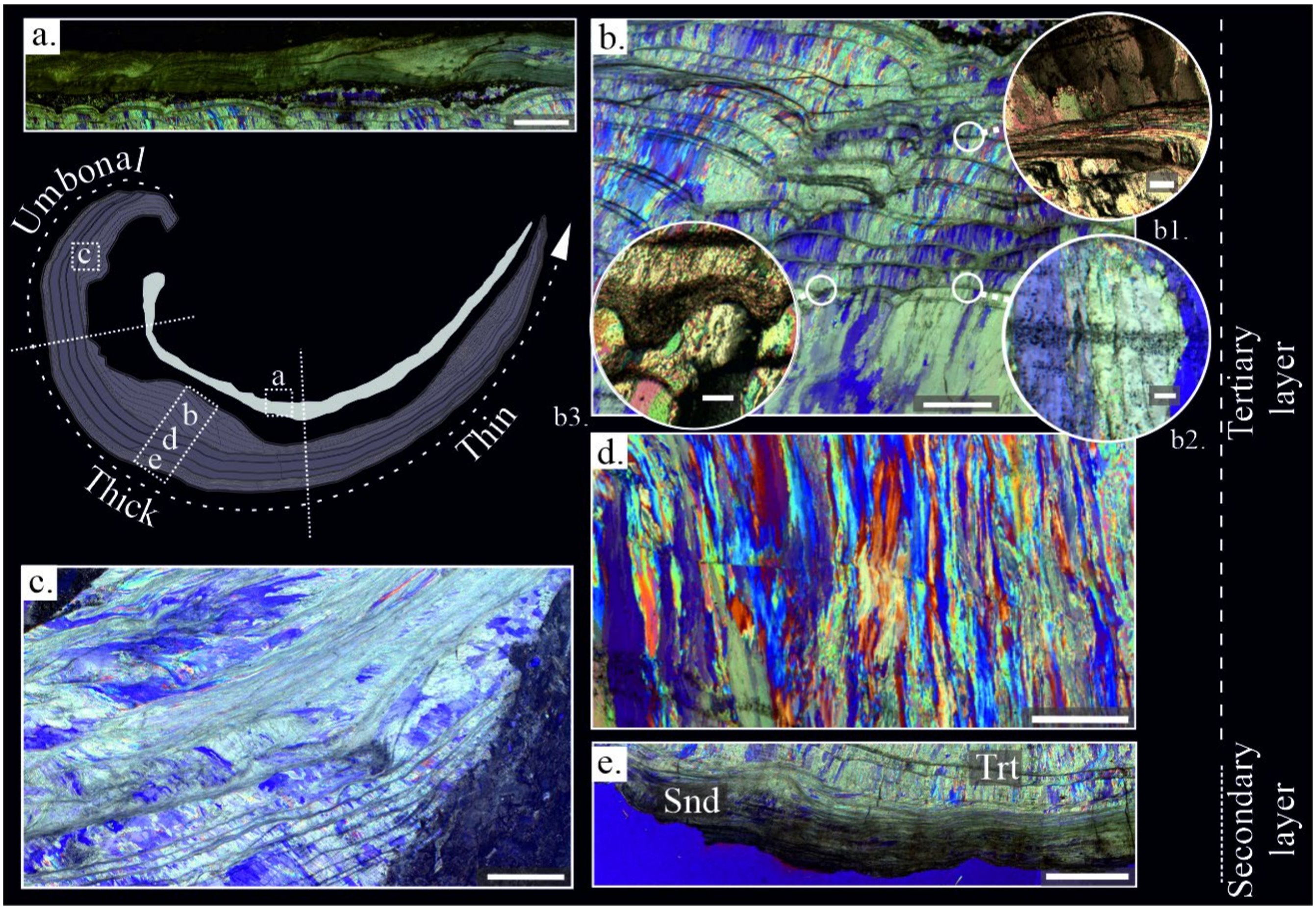

Figure 1 a-e) Microstructural features of *Gigantoproductus* sp. shells under petrographic microscopy. In the left, a scheme of the shell with underscored shell regions (b1 and b3) cross-polarized micrographs; (a, b, b2, c, d, and e) cross-polarized with lambda plate micrographs. In the right, a line which divides the secondary and tertiary layer into the thick region (micrographs b, d, e). a) The laminar microstructure of the dorsal valve with micritized patches (upper part), micrite infilling between the dorsal and the ventral valves, inner region of the ventral valve (lower part). Scale bar 2 mm. b) innermost part of the thick region showing ventral valve with columnar microstructure and several growth lines. Scale bar 1 mm. b1) Inset showing a laminar growth line. Scale bar 100 µm. b2) Inset showing a diffuse growth line. Scale bar 100 µm. b3) Inset showing pseudopunctae in a growth line. Scale bar 100 µm. c) Umbonal region of the ventral valve with several growth lines and a chaotic crystal arrangement. Scale bar 1 mm. d) The thick region of ventral valve with the largest columnar crystals and several growth lines. Scale bar 1 mm. e) Thin region of the ventral valve with columnar microstructure and several growth lines. Scale bar 1 mm.

In the ventral valve, two layers have been distinguished: i) the secondary or laminar layer, which is formed by lath crystals, ~1 µm in width, arranged in packages of thin sheets ~10 to 40 µm in width (Figure 2e). Crystals are mostly of tabular appearance with sharp contacts between the crystals (Figure 3e); ii) the tertiary or columnar layer is formed by large columns (*sensu* [75]) with sizes ranging from 100 to 1700 µm in length and 40 to 150 µm in width (Figure 2d). Occasionally, the tertiary layer show intercalation of laminar growth lines with similar microstructure to the secondary layer embedded between columns. Columnar crystals are composed of submicrometric steps stacked parallel to growth direction (Figure 3d), forming terraces in natural breakage (Figure 3g). Massive crystals growths in columns occur commonly related to thick region (Figures 2d, 3b).

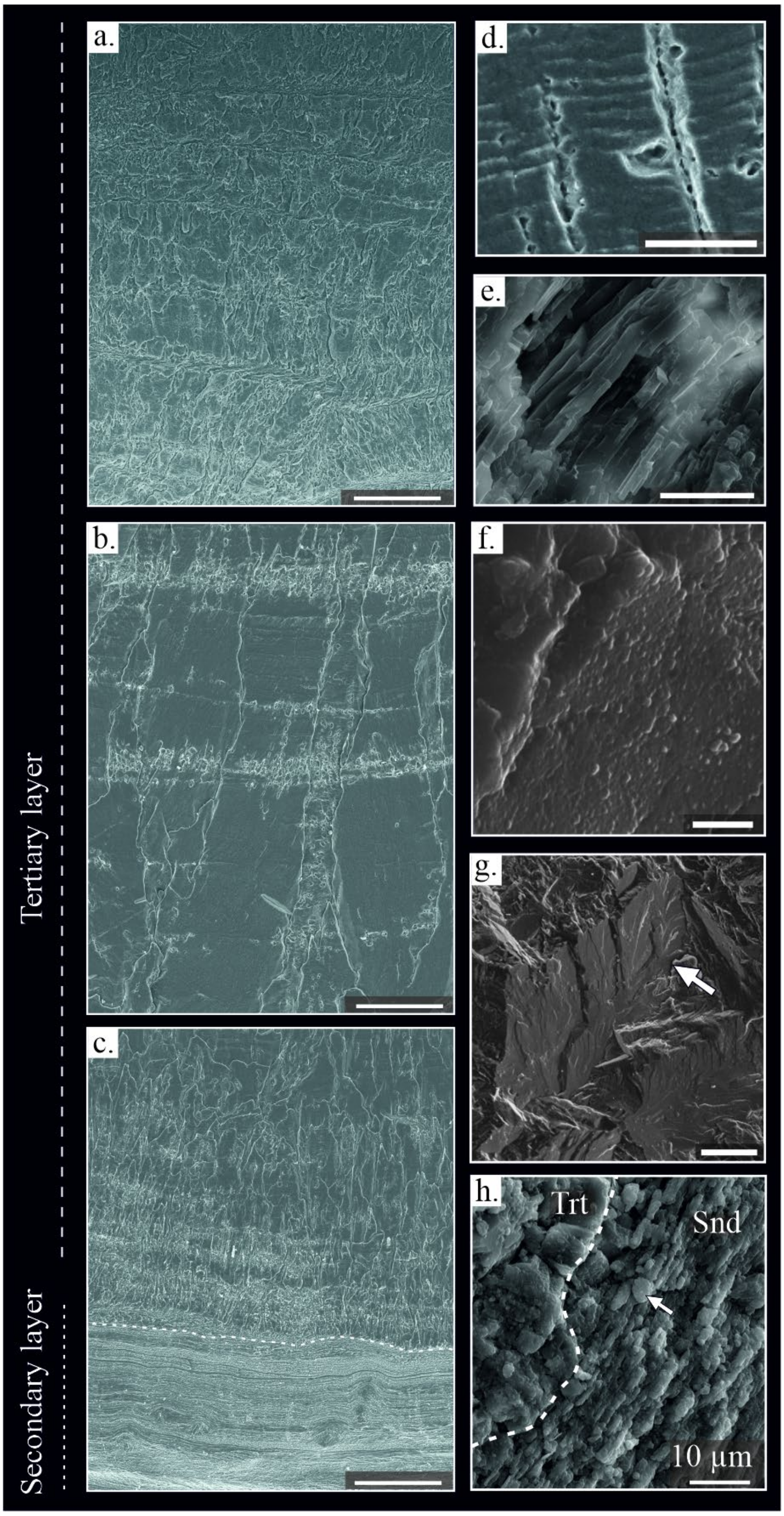

Figure 2. SEM images of shell polished surfaces (a-d). SEM images of natural breakage of the shell; (e, -h). a) Innermost part of the tertiary layer. Scale bar 200 μm. b) Middle part. Scale bar 200 μm. c) Outermost part of the tertiary layer and secondary layer. Scale bar 200 μm. d) Submicrometric laminae preserved in a columnar crystal. Scale bar 10 μm. e) Laminar microstructure composed by lath crystals. Scale bar 10 μm. f) Nanogranular texture in some columnar crystals. Scale bar 2 μm. g) Columnar crystals of the tertiary layer, notice the massive appearance. Scale bar 100 μm. h) Contact between columnar and laminar microstructure (dashed line) in a growth line with a granular appearance of laminae. Scale bar 10 μm. Trt: tertiary layer, Snd: secondary layer.

Likewise, two types of microstructural changes parallel to the shell growth have been identified in the tertiary layer, called growth lines: i) dashed and diffuse aspect (Figure 2b2) with several dark dots (*i.e.*, opaque under transmitted light and in BSE), which passes through the columnar crystals, ~50 to 100 μm of thickness; ii) laminar microstructure (Figure 2b1) densely packed and ~30 to 80 μm of thickness. These growth lines cut off columnar crystals. A variation of the laminar microstructure with a granular appearance is observed in these growth lines.

3.2. Fossil shell preservation

Fossil preservation has been addressed herein by the combination of cathodoluminescence, textural changes under both optical microscopy and SEM, crystallographic arrangement of microstructures and geochemical signatures (*i.e.*, minor and trace elements and Sr isotopes). This set of techniques has been applied to distinguish between well-preserved and poorly-preserved shell regions, with the aim to compare the luminescence and geochemical differences between the different shell region and the relationship with the microstructure.

3.2.1. Textural changes

Microtextural changes have been recognized in some areas of the intra-shell surface due to diagenetic processes. Fractures of the shell are usually oriented perpendicular, or forming a high angle to shell surface, starting from inner shell to the outer shell and usually filled by micrite, sparite or iron oxides. On the other hand, thinner fractures, with micrite filling, parallel to shell surface has been observed in the centre of some ventral valves. Some samples expose delamination processes near the shell surface between columnar crystals separated by laminar growth lines.

Changes in the crystal size and the orientation of the tertiary layer are produced in adjacent zones of fractures or near to edges of the shell. These areas contain a decreasing trend in crystal size towards the outer shell with a mosaic appearance. Some areas exhibit micritisation of secondary layer and degrading neomorphism in the tertiary layer. These recrystallized zones are recognized by small and equigranular crystals, which totally obliterate the primary microstructure. Moreover, other zones contain partially dissolved crystals with a cloudy appearance, where the primary microstructure is roughly recognized. Rounded dark dots are opaque inclusions and are mostly concentrated in growth lines (Figure 2b2), whereas other scattered inclusions have a sharp edges and different interference colours.

3.2.2. Crystallographic arrangement

The observation of crystallographic patterns is a precise approach for the evaluation of diagenesis of polycrystalline skeletons formed by microcrystals with preferred crystallographic orientations and a notable arrangement in supra-specialized structures [29]. This criterion has been previously applied in brachiopod shells achieving excellent results in experimental diagenesis and fossil shells [76–78]. In addition, crystallographic assessments of other calcium carbonate skeletons have been successful [65–67]. This approach is based on the principle that controlled mineralized shells have constrained crystallographic arrangements (*i.e.*, preferred orientations), and which, after diagenesis, can be compromised and substituted by random orientations and/or loss of textural features.

Seven areas of five ultra-thin sections of *Gigantoproductus* sp. ventral valves have been studied after sampling for geochemical analysis (Figure 4). CIP method is limited to assess the crystallographic organisation based on azimuth and inclination of the *c*-axis. Criteria to identify diagenetic alterations are based on abrupt changes in *c*-axis orientation (highly disoriented), and textural changes without or with subtle *c*-axis variations.

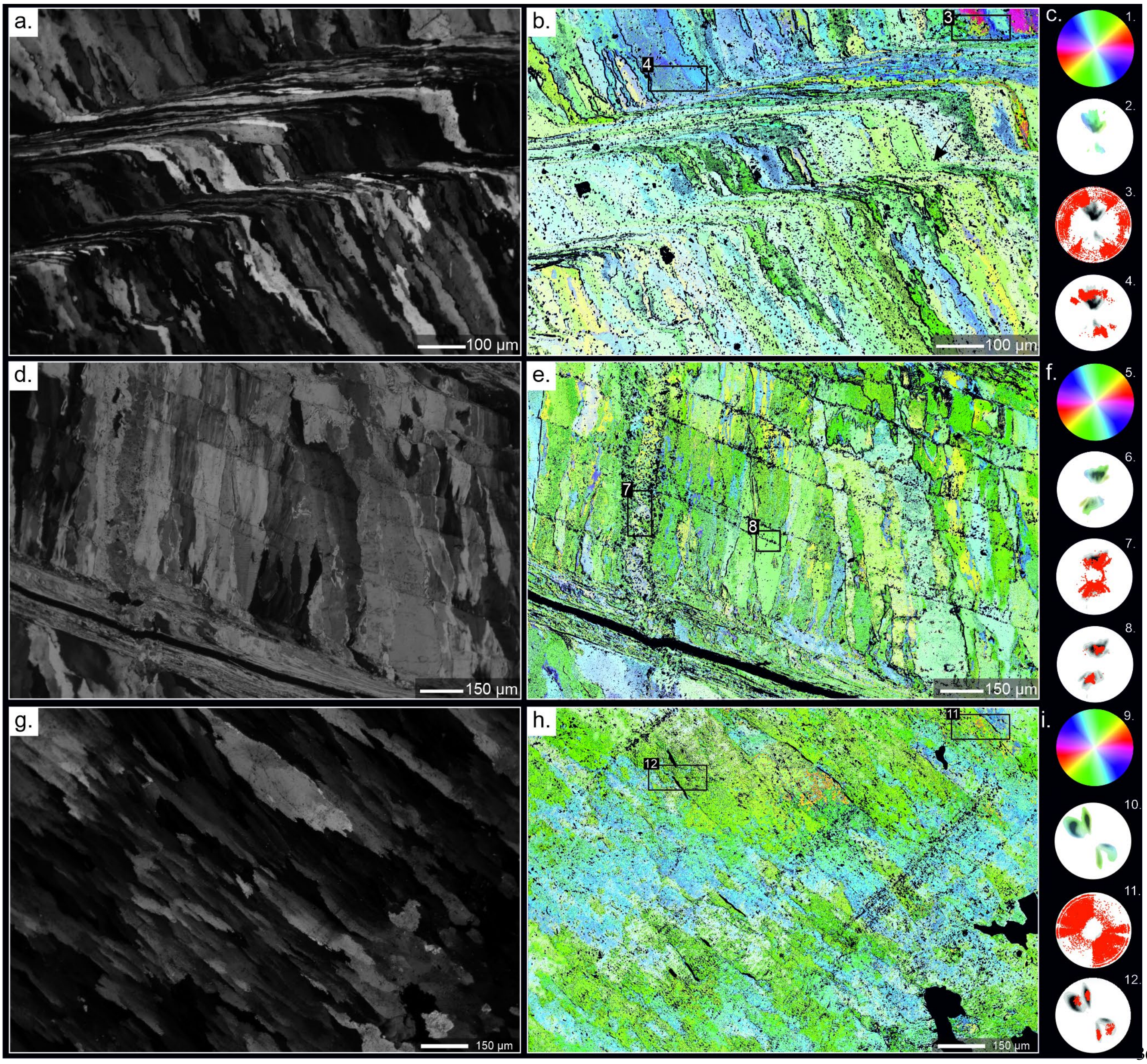

Figure 4. CIP analysis of thin regions of *Gigantoproductus* ventral valves (a,d) and thick region (g). a,d,g) Orientation images. b,e,h) Petrographic micrographs of the studied areas. c,f) Standard colour look-up table (CLUT) and pole figures. 1, 5, 9) CLUT of the orientation images. 2-4, 6-8, 10-12) Pole figures. (2) Corresponds to the complete area (b), showing a pole maximum with *c*-axis is strictly oriented perpendicular to the growth direction of *Gigantoproductus* shell. (3-4) Corresponds to selected areas. (3) Columnar area with random *c*-axis orientations due to recrystallisation. (4) Equivalent area to (3) showing a well-constrained *c*-axis orientation. (6) Corresponds to the complete area (d). (7-8) Corresponds to selected areas. (7) Fracture with degrading neomorphism, which preserves the original crystallographic orientation. (8) Equivalent area to (7) showing a better constrained *c*-axis orientation. Pole figures were calculated as an orientation distribution function and provided in multiples of uniform distribution intervals of 0.5 for *c*-axis orientations of the complete area. Red points correspond to the punctual *c*-axis orientation of each pixel of selected areas. (10) Corresponds to the complete area (g), showing a pole maximum with *c*-axis is strictly oriented perpendicular to the growth direction of *Gigantoproductus* shell. (11-12) Corresponds to selected areas. (11) Prismatic area with random *c*-axis orientations due to recrystallisation. (12) Equivalent area to (11) showing a well-constrained *c*-axis orientation.

The *c*-axis is strictly oriented perpendicular to the growth direction of *Gigantoproductus* shells (*i.e.*, parallel to the radial axis of the valve), regardless of microstructure, shell region and texture, although some variations have been recognized. Columnar and

laminar microstructures exhibit different *c*-axis orientations: parallel to the elongation axis of crystals in the case of columnar microcrystals and perpendicular in the case of laminar crystals (Figure 4, Figure S2). In those areas where the laminar microstructure changes gradually to columnar, and vice versa (*e.g.*, in a growth line), *c*-axis is kept constant between the interconnected crystals (Figure 4b), similar to kinked crystals (*sensu* [79]).

All studied areas, which include all shell regions identified, demonstrate preferred orientations (based on pole figures) with quite narrow azimuthal dispersion. However, some distinctive organisations have been observed in some shell regions. Usually, *c*-axis orientations in the tertiary layer show two narrow pole maxima, with an azimuthal dispersion between them of ca. 55° in the umbonal region (Figure S2e) and ca. 35° in the thick region (Figure S2h,k). On the other hand, thin region with columnar and laminar microstructures exhibit a single pole maximum with a variable azimuthal dispersion: between 35°- 42° in thin region (Figure 4b,e; Figure S2b).

Evidence of diagenetic alteration based on crystallographic orientation and textural changes occurs in some areas. In those areas, where the original microstructure is apparently well-preserved and lamellar twinning is notable, they are characterized by random *c*-axis orientations in twinned crystals and surrounding areas (hundreds of microns around, Figure 4b,h). These areas correspond mostly with thin fractures without displacement, favoured by microstructural changes (*e.g.*, columnar to laminar or contact between columnar crystals, Figure 4h). In addition, some of these areas have the original microstructure completely obliterated by recrystallisation processes (Figure S2b) and exhibit random azimuthal orientations forming large patches with similar characteristics. On the other hand, some areas surrounding fractures are characterized by a degrading neomorphism with co-oriented microcrystals compared to the main crystallographic orientation of the shell (Figure 4e), although the azimuthal dispersion of orientations in this region is larger than individual crystals (*i.e.*, columnar, and laminar crystals). In the figure 4g-h the diagenetic alteration is confined by a growth line, involving only a part of the underlying crystals. This area is recrystallized because the original texture is lost, and random crystallographic orientation occurs.

3.2.3. Cathodoluminescence (CL)

Samples under CL exhibit nearly completely non-luminescent areas (NL) (Figure 5), which correspond mostly to areas with well-ordered crystals of the tertiary layer (Figure 5a,c), some recrystallized zones (*e.g.*, degrading neomorphism, Figure 5c), and fractures with iron oxides cement (opaque in petrographic microscopy, Figure 5b). Slightly luminescent areas (SL) are concentrated close to shell edges, in the laminar microstructure of the ventral and dorsal valves (Figures 5f,i), in fracture zones with associated recrystallized areas (Figures 5d-e), in areas with degrading neomorphism (Figure. 5i), in micritisation zones close to the shell margin (Figure 5h), in growth lines (Figure 5g). High luminescent areas (L) correspond to the surrounding and infilling matrix (Figure 5f,h), sparite cements of inner space of shell (Figure 5f,g), cemented borings (Figure 3j), large cemented fractures (Figs. 5e,h), highly interconnected fractures or fracture zones (Figs. 35,g,h), micritisation areas close to the shell edge and some areas with neomorphism (Figure 5i).

The luminescence in columnar crystal decreases from edges to inside (Figure 5), as well as in degrading neomorphism areas (Figure 5i). The luminescence of growth lines increases in those areas affected by fractures in comparison with the surrounding area (Figs. 5d,g).

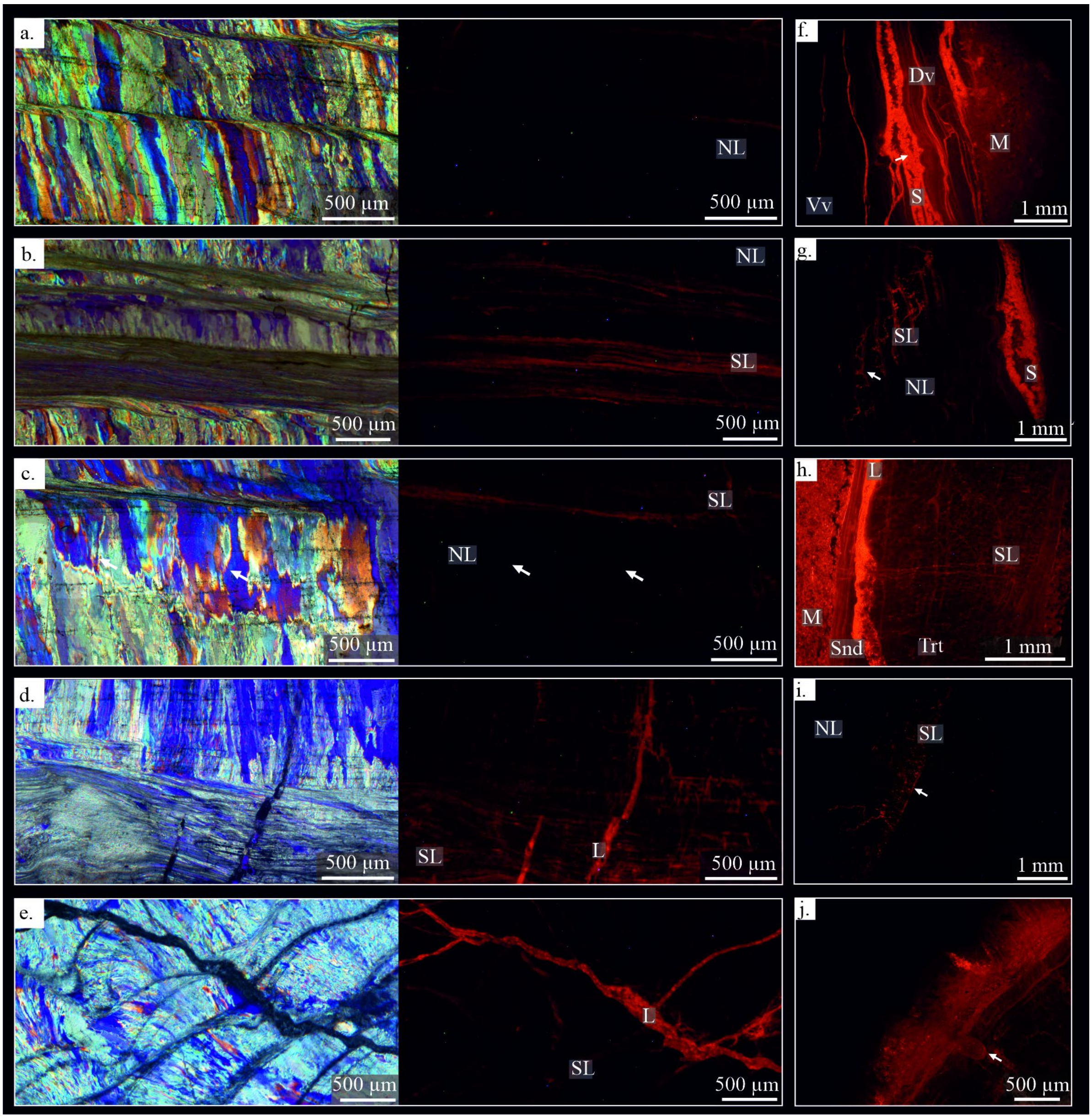

Figure 5. Fossil preservation. a-e) cross-polarized with lambda plate micrographs (left) and the same area under cathodoluminescence (right); f-j) cathodoluminescence micrographs in other shell regions. a) Non-luminescent well-preserved area under CL. b) Well-preserved area with luminescence concentrated in growth lines. c) Non-luminescent poorly-preserved area with a recrystallisation patch in the centre, evidence of recrystallisation. NL fracture (left arrow) d) SL-luminescence poorly-preserved area with recrystallisation by fractures. e) Highly luminescent poorly-preserved area with fractures. f) CL image showing a laminar growth line with the equivalent luminescence areas than fluorescence image. g) Alteration patch in the ventral valve with altered growth lines more luminescent than well-preserved areas. Luminescent sparite in the right of the image. h) The altered external edge of the shell, more luminescent than the central and inner parts of ventral valve. i) Degrading neomorphism in the shell edge, with decreasing luminescence towards the interior. j) Encrusting sponge over *Gigantoproductus* shell, notice the luminescent burrow (arrow). NL: non-luminescent, SL: slightly-luminescent, L: luminescent S: sparite.

3.3. Geochemical characterisation

Geochemical analyses have been conducted in well- and poorly-preserved areas. Major, as well as minor and trace element (MTE) contents and isotope compositions are documented in Tables 1, 2 and 3.

Table 1: Descriptive statistics of major, minor and trace element (MTE) composition of five preservation states identified

Preservation state	Element	N total	Mean	S.D.	Minimum	Median	Maximum
	Ca		390449.9	6880.8	372632.4	391045.6	406978.5
	Mg		3380.5	911.3	1218.3	3238.6	5886.3
	Sr		1737.9	430.5	642.7	1767.3	2968.1
WP-NL	Mn	205	155.3	214.3	0.0	0.0	836.6
	Fe		184.3	263.0	0.0	0.0	1026.2
	S		1060.2	328.8	388.5	993.2	1894.4
	Na		1038.2	358.4	0.0	1031.0	2202.8
	Ca		372422.6	29616.5	269479.6	380938.4	400052.1
	Mg		4651.7	1876.9	645.3	5020.8	7273.4
	Sr		1763.1	473.2	465.1	1839.2	2443.8
PP-NL	Mn	20	278.9	467.2	0.0	27.1	1828.1
	Fe		955.4	1710.0	0.0	427.6	6499.1
	S		1490.5	929.9	0.0	1620.0	3576.5
	Na		926.0	574.8	0.0	997.6	2017.4
	Ca		382121.0	8596.2	365870.4	382139.2	398808.4
	Mg		4164.8	1044.6	2291.8	3959.4	6748.7
	Sr		2208.9	444.7	1166.9	2262.0	3027.2
WP-SL	Mn	44	314.9	367.3	0.0	170.4	1332.3
	Fe		371.2	408.2	0.0	268.2	1391.5
	S		994.7	373.6	460.6	979.2	1974.5
	Na		932.9	358.0	311.5	867.8	1713.3
	Ca		378873.3	14125.2	313975.9	383615.3	398608.2
	Mg		4378.5	1468.8	1724.9	4010.6	9661.7
	Sr		1975.1	501.0	879.4	1894.1	3729.1
PP-SL	Mn	80	125.0	220.3	0.0	0.0	1038.0
	Fe		451.7	880.7	0.0	139.9	6499.1
	S		1202.5	565.0	4.0	1125.4	3576.5
	Na		920.4	378.3	29.7	934.5	2010.0
	Ca		382641.0	8793.2	358236.3	383947.7	394598.2
	Mg		4829.2	1635.3	2249.6	4589.6	7786.0
	Sr		1981.5	473.8	1217.7	2232.4	2765.1
PP-L	Mn	15	582.0	292.4	302.1	457.0	1332.3
	Fe		56.0	89.6	0.0	0.0	287.6
	S		1264.5	462.3	596.7	1093.4	1918.4
	Na		966.7	276.5	504.4	1023.5	1550.2

WP-NL: well-preserved non-luminescent; PP-NL: poorly-preserved non-luminescent; WP-SL: well-preserved slightly-luminescent; PP-SL: poorly-preserved slightly –luminescent; PP-L: poorly-preserved luminescent; S.D. Standard deviation.

452

Table 2: Descriptive statistics of major, minor and trace element (MTE) composition of tertiary layer shell regions and secondary layer.

Shell part	Element	N total	Mean	S.D.	Minimum	Median	Maximum
	Ca		388430.1	6175.9	373604.5	389194.3	398379.5
	Mg		3259.7	840.4	1899.8	3178.3	5735.5
	Sr		1604.6	551.8	642.7	1648.9	2570.6
U	Mn	33	227.5	255.8	0	170.4	836.6
	Fe		205	266.8	0	38.9	1026.2
	S		930.1	303.3	440.6	893.1	1786.2
	Na		1131.9	337.6	526.6	1105.1	1995.2
	Ca		393264.2	6774.8	372632.4	394433.8	406978.5
	Mg		3012.1	722.7	1218.3	2943.1	4999.7
	Sr		1656.4	408.8	845.6	1623.6	2460.7
Tk	Mn	97	140.9	202.1	0	0	774.6
	Fe		219.4	282.1	0	46.6	925.1
	S		1040.1	371.3	408.5	957.2	2367
	Na		1063.5	401.9	0	1068	2202.8
	Ca		386561.5	7115.3	366270.7	387503.8	397164.3
	Mg		4126.5	1125.4	2056.6	4067.9	7273.4
	Sr		1890.7	330.4	1107.7	1902.6	2968.1
T	Mn	90	160.1	244.8	0	0	1332.3
	Fe		161.4	264.3	0	0	1041.7
	S		1249.2	470.2	224.3	1165.5	3576.5
	Na		968.8	346.5	0	945.7	1579.8
	Ca		379749.4	8141.1	365870.4	378940.5	398808.4
	Mg		4444.0	1076.2	2780.3	4565.5	6748.7
	Sr		2283.4	360.2	1462.9	2367.7	3027.2
Secondary layer	Mn	30	182.3	210.1	0.0	116.2	704.9
	Fe		370.0	453.7	0.0	225.4	1609.2
	S		980.8	288.5	460.6	1039.3	1549.9
	Na		1013.9	385.7	311.5	997.5	1713.3

U: umbonal region; Tk: thick region; T: thin region; S.D. Standard deviation.

Table 3: Isotopic composition of the structural regions identified in the tertiary layer of *Gigantoproductus* shells.

Shell region	δ18O	δ13C	87Sr/86Sr (StdErr)	M.S.D.	Shell region	δ18O	δ13C	87Sr/86Sr (StdErr)	M.S.D.	Shell region	δ18O	δ13C	87Sr/86Sr (StdErr)	M.S.D.
	-3.4	2.78				-2.91	3.35	0.70786						
	-3.61	2.35				-2.77	3.41	(4^-6)			-3.71	1.84		
	-3.78	2.39				-3.13	3.29	0.707845			-2.86	4.27		
Umbornal	-3.55	2.68				-2.99	2.95	(4^-6)			-5.06	3.62	0.707893	
	-3.25	2.52			Thin	-2.93	3.15				-4.12	3.68	(4^-6)	
	-3.14	2.97				-2.33	3.04				-2.32	3.87		
	-3.27	2.81				-2.44	3.17				-6.28	3.08		
**Mean**	**-3.43**	**2.64**		**0.22/0.23**		-2.34	3.38			poorly-	-4.37	1.18		
	-2.82	2.18	0.70783			-2.54	3.29			preserved	-1.64	3.05		
	-3.39	2.1	(4^-6)			-2.27	2.89				-1.89	3.76		
	-2.62	2.25			Mean	**-2.67**	**3.19**		**0.32/0.18**		-3.87	3.3	0.707807	
	-2.92	2.29				-3.25	2.49				-2.16	2.61	(5^-6)	
Thick	-2	3.09			Dorsal	-4.02	1.54				-2.14	2.43		
	-2.58	3.04			Valve	-1.46	3.56				-1.83	3.46		
	-2.33	2.96	0.707831			-3.13	2.13				-4.03	3.34		
	-2.32	2.94	(4^-6)		Mean	**-2.97**	**2.43**		**1.08/0.85**	**Mean**	**-3.31**	**3.11**		**1.4/0.84**
	-2.71	2.86												
**Mean**	**-2.63**	**2.63**		**0.40/0.42**										

M.S.D. Mean standard deviation; StdErr: Standard deviation error.

3.3.1. Major/minor and trace elements (MTE)

MTE helps to evaluate the degree of geochemical alteration of brachiopods comparing cathodoluminescence, crystallographic arrangement and petrographic microimages. *Gigantoproductus* ventral valve show different trace elements concentrations related with diagenetic alteration, layers, and shell regions (Figure S3, Tables 1 and 2).

The proportion of trace elements varies in function of the preservation state (Figure S3, Table 1). In general, well-preserved areas of ventral valve show lower mean standard deviation of the Ca, Mg and S values than the poorly-preserved areas. NL well-preserved areas contain more Ca, less Mg and less dispersion of all elements, except the Sr, than the NL poorly-preserved areas. SL well-preserved areas contain more Mn and Sr, less S, and exhibit lower mean standard deviation of Ca and Mg than SL poorly-preserved. The highest mean of Mn occurs in the luminescent areas (Figure S3, Table 1).

The mol% of $MgCO_3/CaCO_3$ of NL and SL well-preserved areas, for each shell region, represents a linear correlation ($r=-0.98$ $p(a) > 0.05$, $n = 249$), with average values of 98.02 mol% $CaCO_3$ and 1.677 mol%$MgCO_3$, distinctive of low-Mg calcite. In addition, differences between regions are recognized in U-region (98.33 mol% $CaCO_3$; 1.38 mol%$MgCO_3$), Tk-region (98.48 mol% $CaCO_3$; 1.26 mol%$MgCO_3$) and T-region (98.03 mol% $CaCO_3$; 1.68 mol%$MgCO_3$).

Statistics per element (Table 2) of the different brachiopod shell regions demonstrate higher average concentration of Ca and Mn in the umbonal region than in the rest of them. The thick region (Tk) exhibits less proportion of Mg and Mn than U- and T-regions but more Ca. T-region contains slightly more Mg, Sr and S than U- and Tk-regions but lower

Na. Mg and Sr average values are higher in the secondary layer than in the tertiary layer, whereas Ca is lower in the secondary layer.

Ca, Mg, S and Na of all well-preserved areas are show in boxplots of the Figure 6. Mg is higher in the U- and T-regions, whereas Ca is higher in Tk region. Na is lower in the T-region however the S is higher.

Mg/Ca, S/Ca and Na/Ca transects measured in well-preserved areas of three individuals varies from higher concentration near the shell edge, which decreased towards the shell interior (Figure 7). In addition, concentration of Mg, S and Na covaries in the measured transects. In poorly preserved areas (*e.g.*, recrystallized areas) decreasing metal/Ca trend is not observed, and each element is not positively correlated (Figure 7c).

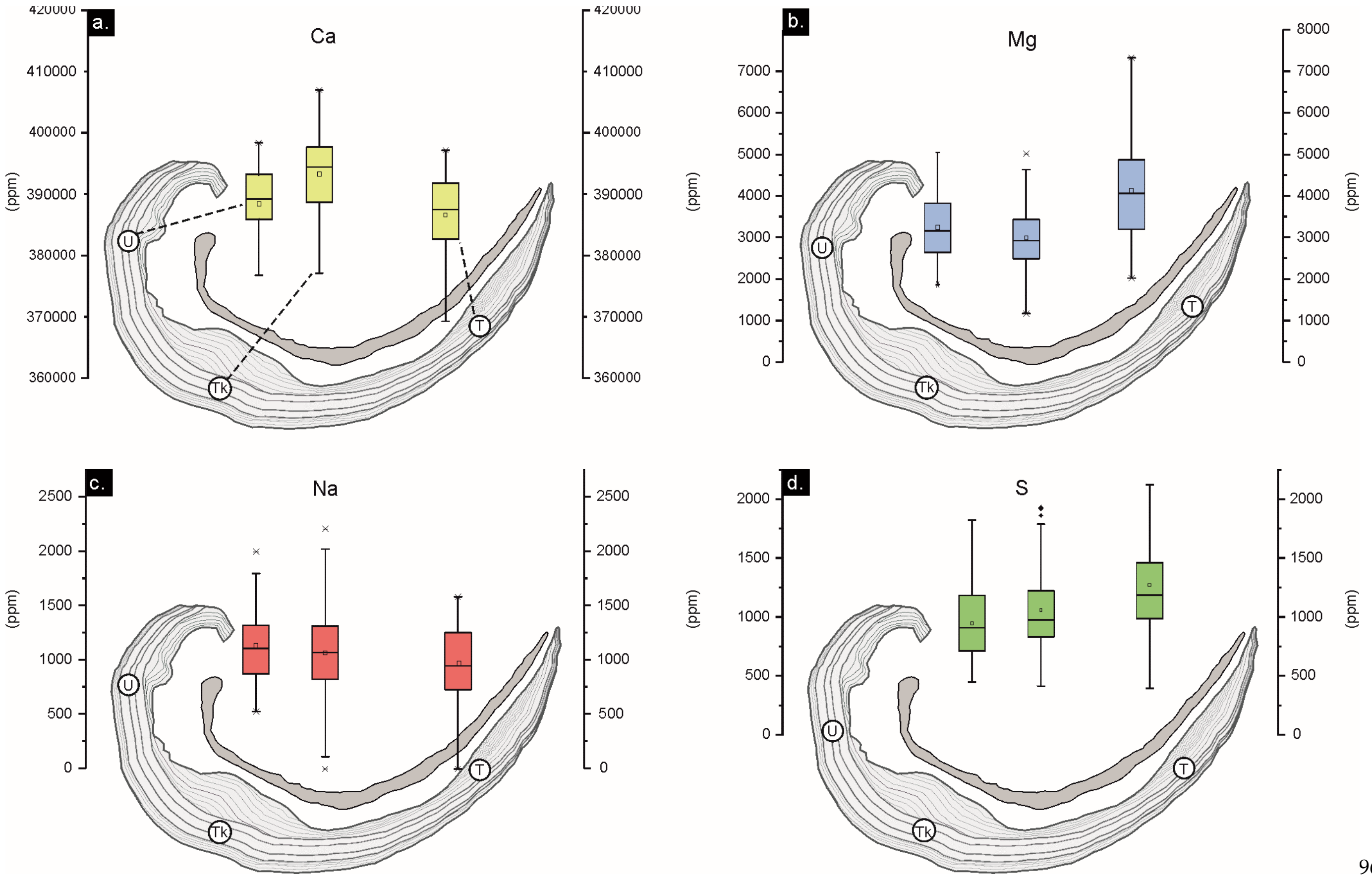

Figure 6. Box plot (a, b, c, d) showing the variation of Ca, Mg, Na and S (in ppm) for the proposed shell regions in *Gigantoproductus* ventral valve. U: umbonal region, Tk: thick region. T: thin region. Ca: calcium, Mg: magnesium, Na: sodium, S: sulphur.

3.3.2. Isotope geochemistry

The values of $^{87}Sr/^{86}Sr$ obtained by TIMS range from 0.707830 to 0.707860 in the NL well-preserved areas (Table 3), while SL poorly-preserved areas are deviated from these values by ±0.00003. A sample is enriched in ^{87}Sr and other is depleted with regards to the well-preserved samples.

The $\delta^{13}C$, $\delta^{18}O$ relationship can be subdivided according to their dispersion degree into poorly-preserved and well-preserved areas and its consistency into the identified shell regions (Figure 7; Table 3). Dispersed values, with a variation of ~4.6 ‰ for $\delta^{18}O$ and of ~3 ‰ for $\delta^{13}C$, occur in poorly-preserved areas, whereas ~1.7 ‰ for the $\delta^{18}O$ and ~1.3 ‰ $\delta^{13}C$ occur in well-preserved areas. Poorly-preserved areas show different alteration signatures, some samples have similar $\delta^{13}C$ but are depleted in $\delta^{18}O$, other are depleted in $\delta^{13}C$ and $\delta^{18}O$ and others are $\delta^{13}C$ and $\delta^{18}O$ enriched (Figure 8; Table 3).

A correlation with each identified shell region is recognized in well-preserved areas (*i.e.*, same drilled regions contain rather similar values. Figure S5). Thus, $\delta^{18}O$ values of well-preserved areas range from -3.8 to -2‰ and from 2.1 to 3.4‰ for $\delta^{13}C$. The U-region concentrates the lowest data of $\delta^{18}O$, with averages of -3.4‰ for $\delta^{18}O$ and 2.6‰ for $\delta^{13}C$ (Table 3). Tk-region exhibits the similar $\delta^{13}C$ data of than U-region but lower $\delta^{18}O$, with averages of -2.6‰ for $\delta^{18}O$ and 2.6‰ for $\delta^{13}C$. Higher $\delta^{13}C$ values are recognized in the T-region compared to the U-region, with averages of -3.2 ‰ for $\delta^{13}C$ and 2.7‰ for $\delta^{18}O$.

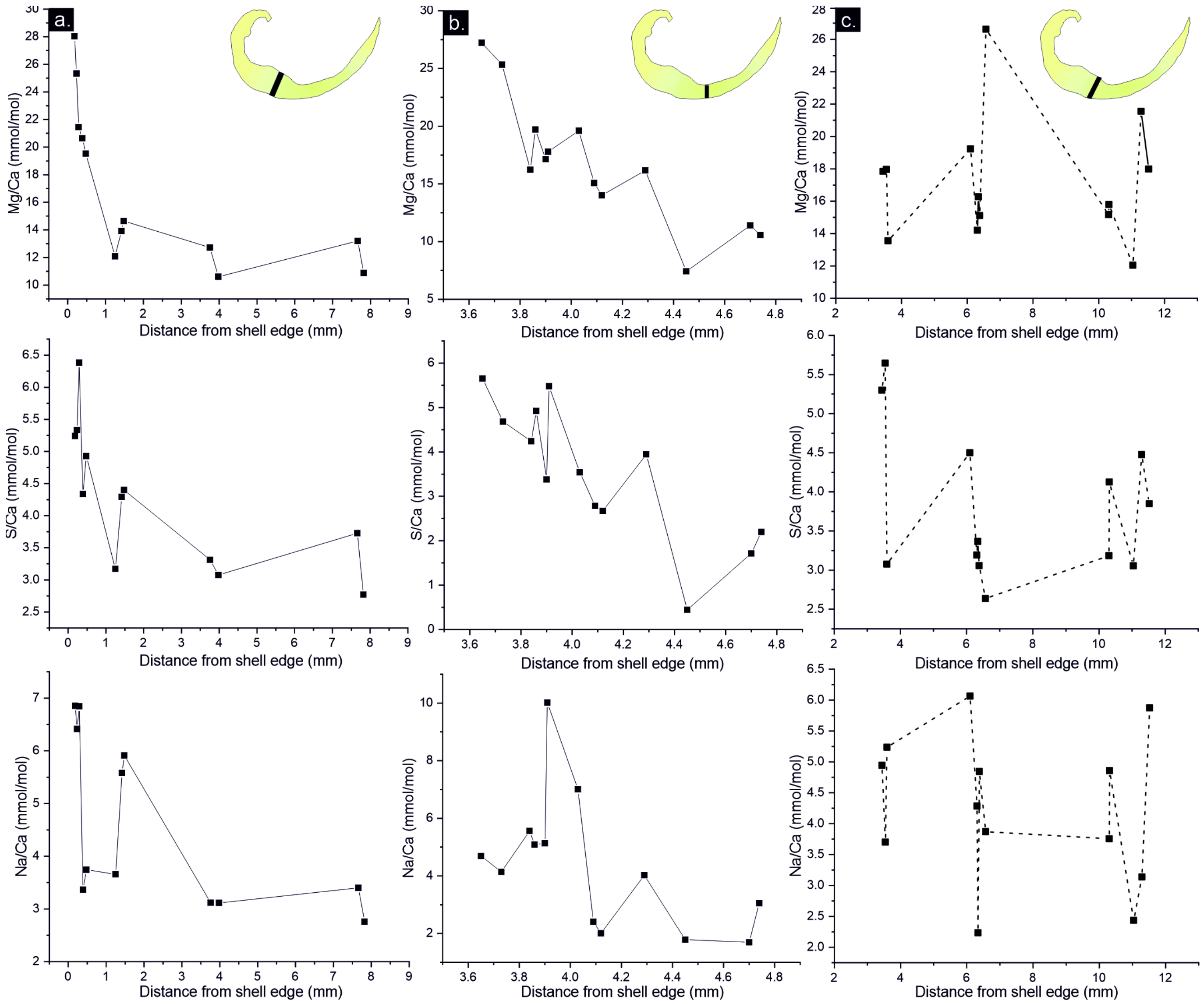

Figure 7 Mg/Ca, S/Ca, Na/Ca transects from the shell edge to the shell interior in well-preserved areas (a,b) and poorly-preserved area (c).

4. Discussion

In this section, the preservation of tertiary layer of *Gigantoproductus* ventral valves and the influence of diagenesis on the geochemical signatures are discussed. Moreover, kinetic, and biological effects that overprint the geochemical signatures suitable for paleoclimatological purposes are assessed.

4.1. Fossil preservation

Gigantoproductus ventral valve can retain their original structural features at macro- and at microscale. The secondary and tertiary layers are well-defined by their microstructure with a controlled preferred crystallographic arrangement (*c*-axis perpendicular to the shell edge). The microstructure is interpreted as unaltered because it retains its original morphological features, similar to those described in some extant brachiopods [79,80]. Likewise, submicrometric laminae have been recognized (Figure 3d) in *columnar* crystals of the tertiary layer, like those described by Schmahl et al. [80] in columnar crystals of *Terebratulina septentrionalis*. Furthermore, preserved nanocrystals, with a granular texture, were identified in some columnar microcrystals by natural breakage (Figure 3. f, h). This finding suggest well preservation (*i.e.*, preservation of biogenic features) in fossil carbonate biominerals [29,81], because they are also characteristic in extant brachiopods [82].

Nonetheless, some areas show diagenetic alteration such as fractures, delamination, borings, micritisation, degrading neomorphism, with subsequent changes in the original crystallographic arrangement (Figure 4, 5, S2).

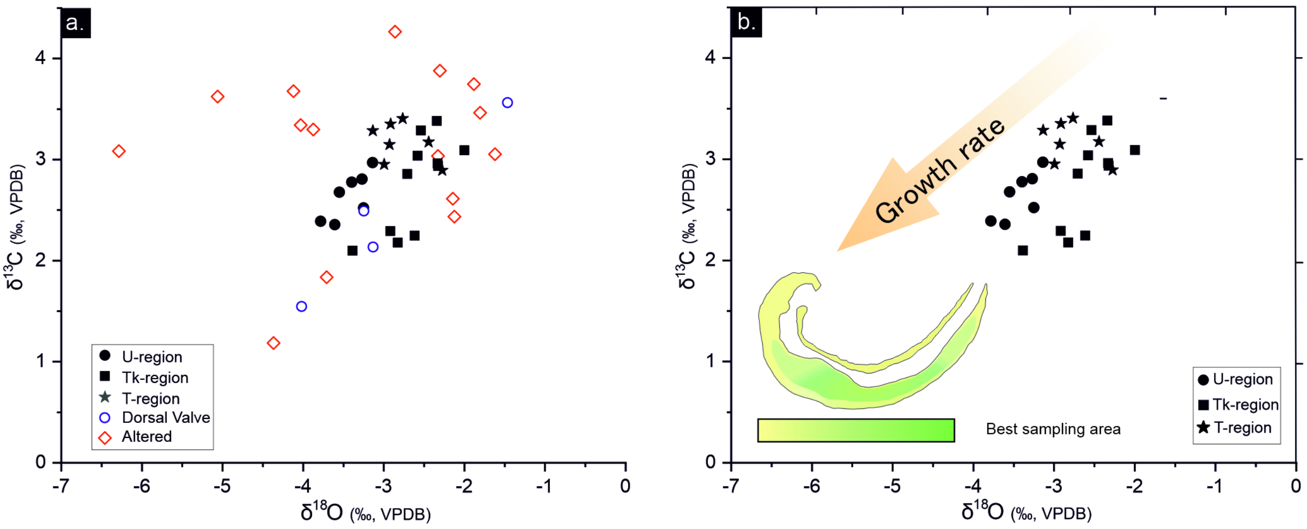

Figure 8. a-b) $\delta^{13}C$ vs. $\delta^{18}O$ diagram of 11 *Gigantoproductus* shells. Well-preserved areas (filled black), dorsal valve (blue circles) and poorly-preserved areas (red diamonds). b) Values of well-preserved areas of the tertiary layer in each region. Scheme of the shell with underscored shell regions. U: umbonal region; Tk: thick region; T: thin region; DV: Dorsal Valve.

The secondary and tertiary layers in *Gigantoproductus* shells are characterized by different microstructure and geochemical composition, as was recognized in other extant and fossil brachiopods [36,43,83]. The secondary layer, in *Gigantoproductus* of this study, is characterized by a laminar microstructure with the elongation axis of crystals oriented parallel to the shell surface and small crystal size in comparison with the columnar microstructure of tertiary layer, where the elongation and *c*-axis are oriented perpendicular to the shell surface and with larger crystals. Therefore, the morphological features of the secondary layer make it more prone to fracture, delamination, and recrystallisation. Within the tertiary layer, the thick region (Tk) of the valve is less affected by recrystallisation and fractures than the thin and umbonal regions (T, U). This may be due to their large crystal size and/or central shell position. On the other hand, the thin dorsal valve contains more fractures than the ventral valve, which makes more susceptible to the alteration and has more micritized patches (Table 4).

The secondary layer is highlighted by a intenser luminescence under CL than the tertiary layer of the ventral valve (Figure 3). Similar differences in CL luminescence intensity between secondary and tertiary layers had been recognized in *Composita subtilita* and

Neospirifer pattersoni [84]. Although classically Mn and Fe are indicative of diagenetic alteration in calcite [85–88], not all luminescence, or its absence, can be correlated to diagenesis [65,75,76,89]. Biogenic carbonates can incorporate small amounts of Mn and Fe during their metabolic activity because of physicochemical variations of the environment in which they develop [89–91].

Table 4: Summary of structural and geochemical features of the five states of preservation identified.

**Features**		**Preservation states**						
		**Well-preserved**			**Poorly-preserved**			
		**Tertiary layer**		**Secondary layer**	**Tertiary layer**	**Growth lines**	**Dorsal valve**	**Secondary layer**
Structural	Microstructure	Prismatic crystals with noticeable microlaminae, preferential orientation	Growth lines: original organic matrix remains and preferential orientation of the crystals	Lath crystals, original organic matrix remains and preferential orientation	Recrystallisation (*e.g.*, degrading neomorphism), sparite cements, filled fractures small patches of crystals without a preferential orientation	Recrystallisation, delamination	Recrystallisation, matrix infilling, micritisation, sparite cements, filled fractures	Recrystallisation, delamination, micritised patches, matrix infilling, filled fractures
	Cathodoluminescence	Non-luminescent	Slightly-luminescent, luminescence pattern in growth lines	Slightly-luminescent, luminescence banding	Non-luminescent	Increasing luminescence near fractured areas	Luminescent	Slightly-luminescent
Geochemical	Crystallographic	Highly arranged crystals: c-axis oriented perpendicular to shell growth	Highly arranged crystals: c-axis oriented subparallel to shell growth	Highly arranged crystals: c-axis oriented parallel to shell growth	Poorly arranged crystals: c-axis randomly oriented			
		**(ppm)**						
	MTE	Mg: 3380, Sr: 1737, Mn: 155, Fe: 184	Mg: 4164, Sr: 2208, Mn: 314, Fe: 371	Mg: 4444 Sr: 2283 Mn: 182 Fe: 370	Mg: 4651, Sr: 1763, Mn: 278, Fe: 955	Mg: 3765, Sr: 1922, Mn: 971, Fe: 858	–	Mg: 4849, Sr: 2323, Mn: 117, Fe: 838
	Stable isotopes	Low dispersion of the ratio, δ18O: -3.78 ‰ to -2 ‰; δ13C: 2.09 ‰ to 3.4 ‰; grouped in clusters depending on ontogeny development			High dispersion of the ratio, δ18O: -6.28 ‰ to -1.83 ‰; δ13C: 1.18 ‰ to 4.27 ‰			
	Sr-isotopes	Low dispersion of the ratio: 0.707830 - 0.707860			High dispersion of the ratio: 0.707807 - 0.707893			

Luminescence differences in *Gigantoproductus* might be related with variations in the chemical composition, thus, the secondary layer is slightly enriched in Mn and Mg (Table 2), possibly related to more organic matrix remains (Figure 3h). In addition, some fractures with higher amount of Fe are non-luminescent under CL and growth lines, with apparently more Mn concentration, are luminescent under CL. This is partly due to Mn is a luminescence activator and Fe is an inhibitor of CL [88]. High Sr and Fe concentrations in some areas of secondary layer suggest alteration by fluids favoured by delaminated areas.

Slightly luminescent areas with the well-constrained crystallographic arrangement (considered here as well-preserved) have been identified in *Gigantoproductus*. These areas exhibit a luminescence pattern under CL that corresponds to the growth lines, and in turn, with a slightly higher Mn content showed by punctual analyses. This luminescence pattern under CL seems to be the result of organic matter and/or Mn [92] incorporated during shell growth. These areas also contain more Mg and S content, but no extensive Mn changes. The determination of small amounts of organic inclusions in growth lines (Figure 3h), higher concentrations of Mg, and Mn across the growth lines, suggest a combination of both sources, probably induced during the biocrystallisation process. Similar luminescence patterns under CL were reported in growth lines of extant brachiopod *Megerlia truncata* [75], fossil brachiopods [89,92] and belemnite rostra [93]. Higher organic concentrations are recognized when the secretory regime of calcite is reduced [89]. This fact has been interpreted as: growth cessation during environmental stress events, produced by mantle anaerobiosis, or by acidification of calcification fluid by the closure of the valve [92,94,95]. Evaluation of these hypotheses deserves further study.

As described previously, not all non-luminescence areas of *Gigantoproductus* shells correspond to well-preserved zones. Crystals with disordered *c*-axis orientations have been identified in non-luminescent areas under petrographic microscopy. This may be due to slow recrystallisation processes, which keep the original microstructure but change the orientation of *c*-axis. Examples of this process can be observed in coral skeletons [66,69]. Therefore, luminescent areas are not always indicative of diagenetic alteration [89] and non-luminescent areas are not always indicative of good preservation [29,37]. Banner and Kaufman [18] and Barbin and Gaspard [89] documented non-luminescent areas with altered $^{87}Sr/^{86}Sr$ and $\delta^{18}O$ values in fossil brachiopod shells and slightly luminescence areas without evidence of alteration in extant brachiopod, respectively.

The combination of CL with geochemical analyses helps to evaluate the diagenetic alteration [65], but it is necessary a well understanding of the original brachiopod shell chemistry before using geochemical features as a diagenetic indicator, considering those biotic and abiotic factors that could control the original shell chemistry [36]. Chemical composition of *Gigantoproductus* shells in this study is equivalent to that in gigantoproductid reported by Popp et al. [17], Bruckschen et al. [20], Armendáriz et al. [9], Angiolini et al. [34,35] and Nolan [32]. The luminescence pattern under CL in well-preserved areas is similar to those described by Angiolini et al. [34,35] and additionally, a similar luminescence of diagenetically altered areas was described by Armendáriz et al. [9] and Nolan [32].

Minor and trace elements have been classically used to evaluate brachiopods shell preservation by comparison between individuals, genera and with extant unaltered brachiopod shells [17,28,36]. NL and SL poorly-preserved areas of tertiary of *Gigantoproductus* (Figure S3, Table 1) have ~800 ppm more Mg than well-preserved areas. Moreover, NL poorly-preserved areas have ~800 ppm more Fe and ~120 ppm more Mn than equivalent well-preserved areas. These values of poorly-preserved areas probably reflect the influence of Mg-, Fe-, Mn-rich fluids during diagenesis in different stages (burial and meteoric waters). These data agree with the observations made by Grossman et al. [36] in altered fossil brachiopods shells, which are Fe- and Mn-enriched.

Additionally, the diagenetic alteration of valves modifies the isotopic record of $\delta^{18}O$, $\delta^{13}C$ and $^{87}Sr/^{86}Sr$. Poorly-preserved areas exhibit $\delta^{18}O$ and $\delta^{13}C$ larger standard deviations than the well-preserved areas (Table 3). Five samples of poorly-preserved areas (Figure 8)

are depleted in $\delta^{18}O$ with similar $\delta^{13}C$; two samples are depleted in $\delta^{18}O$ and $\delta^{13}C$; three samples are enriched in $\delta^{18}O$ and $\delta^{13}C$; and two has similar $\delta^{18}O$ and enriched in $\delta^{13}C$ respect to the well-preserved areas.

In relation to Sr isotopes, well-preserved areas contain homogenous $^{87}Sr/^{86}Sr$ ratios, in contrast with poorly-preserved areas (one $^{87}Sr/^{86}Sr$-enriched sample and one $^{87}Sr/^{86}Sr$-depleted sample). Sr isotope values from *Gigantoproductus* (from 0.707830 to 0.707860) are equivalent to those showed by Bruckschen et al. (1999) in brachiopods from Pendleian substage (mostly gigantoproductids shells, from 0.707828 to 0.707879), which validate the well preservation of the samples, except two samples.

Different signatures of stable isotopes ($\delta^{18}O$ and $\delta^{13}C$), trace elements (Mg, Mn, and Fe), and Sr isotopes suggest the co-existence of different diagenetic processes and stages acted over the *Gigantoproductus* shells, which is also supported by the showcase of microstructural and crystallographic changes.

On the other hand, the $\delta^{18}O$ and $\delta^{13}C$ trends in well-preserved areas can be explained by other mechanisms, such as kinetic and biological variations of ionic and isotopic values from equilibrium seawater ('vital effects').

4.2. Biological overprint of geochemical signatures

'Vital effects' are geochemical deviations from the thermodynamic equilibrium of seawater produced by organism vital processes, such as kinetic effects during ontogeny. Curry [96] observed three growth phases with different growth rates in *Terebratulina retusa*: fast growth rate during the first stages of growth (umbonal region), followed by a constant growth period of three years and progressively decreasing growth rate with brachiopod ageing. Variations in Mg across the shell have been widely studied in the literature as indicative of growth rates. Buening and Carlson [56] observed higher Mg amount near the umbo of extant brachiopods, which decreases during brachiopod ageing.

Tertiary layer of *Gigantoproductus* ventral valve can be divided in structural regions (umbonal, thick and thin regions) and growth stages (younger and older) because during ageing shell grows in length (from umbo to commissure) and thickens (towards valve interior) as a coupled process. Therefore, U-, Tk- and T-regions must include younger (outermost parts of the shell) and older areas (innermost parts of the shell), which may disguise some kinetic fractionation, just considering the mean values of each area. For instance, structural regions exhibit differences in averaged Mg: the umbonal region has more Mg than the thick region (Tk) and thin region (T) has the highest amount of Mg. On the contrary, analysed transects in function of distance exhibit a Mg depletion towards the interior from outermost part of ventral valve. Tk-region shows an exponential variation of Mg in contrast to T-region, which varies linearly.

Different Mg trends have been identified in extant brachiopod shells: an Mg-enrichment with ageing in the extant brachiopod shell *Magellania venosa* and parabolic Mg trend with ageing in extant brachiopod shells *Liothyrella neozelanica* and *Gryphus vitreus* [51]. Variations in Mg concentration of brachiopod shells seems to be species-specific [54,97], with different distribution trends between species ([51] or into the same species [29,54]. Moreover, Rollion-Bard et al. [43] showed differences in the Mg/Ca incorporation across the shell in different extant brachiopod species, emphasizing that Mg does not incorporate by a simple pathway during ontogeny. These differences may be related to different growth rates [56], crystallographic features [45], different proportion of organic components into the shell, seawater temperature and pH variations [42], and/or Mg exclusion from the calcification fluid [43]. Besides Mg variation, S and Na vary from outer edge of ventral valve of *Gigantoproductus* towards the interior. Microstructure of tertiary layer shows abundant smaller crystals located in the interphase between the secondary and tertiary layers (initial biocrystallisation event of tertiary layer, Figure 2d), where nanograins and organic inclusions are frequent. Remarkably, these areas with small crystals are Mg-S- and Na-enriched, and during ageing through the shell crystals enlarge and these elements decrease towards the interior.

Na in *Gigantoproductus* seems positively correlated with Mg and S (Figure 7) in the well-preserved areas. Na concentration in carbonates can be influenced by different processes that might explain this trend:

i) In relation with growth rate, like Mg. Na incorporation may be favoured by crystalline defects, in addition to the higher precipitation rates in abiogenic calcite and biogenic calcite [98,99].

ii) Na can occupy an interstitial position of carbonate lattice or can form part of carbonate lattice by an altervalent substitution, creating a vacancy in the lattice [100,101].

iii) Na can form part of organic matter in biogenic carbonates. Rollion-Bard and Blamart [99] described a positive correlation between Na/Ca and Mg/Ca with organic matter in bioaragonite of corals.

iv) Diagenetic processes. Na composition in carbonates can be strongly influenced by diagenesis more than by environmental processes [102].

Mii et al. [103] showed a positive correlation between Mg, S and Na in fossil samples of *Neospirifer dunbari*, similar to *Gigantoproductus* of this study, supporting the hypothesis of these trends are original signatures indicative of well-preserved areas in fossils.

Mg, Na and S incorporation into crystal lattice have been associated with crystal growth rates in crystal growth experiments [98,104]. Moreover, growth rates associated to trace element partitioning has been observed in both, extant and fossil brachiopod shells [36,56,103]. Mii et al. [103] and Grossman et al. [36] related the high concentrations of these elements with seasonal growth rates, increasing during summers and decreasing during winters. Intra-shell small variations in Mg, Na and S in *Gigantoproductus* shells transects (Figure 6) may be explained by this interpretation, despite the overprinted depletion trend from the shell edge towards the interior by brachiopod ageing.

All specimens of *Gigantoproductus* exhibit a depletion of $\delta^{13}C$ and $\delta^{18}O$ from older regions (Tk- and T-regions) to younger regions (U-region). It is remarkable that data from thick region (Tk) are split in two clusters (Figure 8): one near shell edge (younger), more depleted in $\delta^{13}C$, and another close to the shell interior (older), enriched in $\delta^{13}C$. Fractionation of $\delta^{13}C$ and $\delta^{18}O$ in *Gigantoproductus* shells exhibit an inverse trend to those observed in Mg, S and Na partitioning. Younger regions with higher growth rates are Mg-, S-, Na-enriched and $\delta^{13}C$ and $\delta^{18}O$-depleted, whereas older zones are Mg-, S-, Na-impoverished and $\delta^{13}C$ and $\delta^{18}O$ -enriched. Preferential isotope fractionation has been previously described in extant and fossil brachiopods: for instance, Auclair et al. [49] described a $\delta^{13}C$ and $\delta^{18}O$ depletion from the outermost (younger) to the innermost (older) part in the secondary layer in secondary layer of *Terebratalia transversa.* Similarly, Batt et al. [24]recognized $\delta^{13}C$ depletion in the secondary layer of a fossil productid and $\delta^{13}C$ and $\delta^{18}O$ depletion in tertiary layer from outermost (younger) to innermost (older) of the genus *Composita* shell. Isotope fractionation was related to growth rate in brachiopod shells [24,49,50].

$\delta^{13}C$ and $\delta^{18}O$ direct and inverse covariations trends have been reported in extant and fossil brachiopod shells [24,42]. Moreover, the depletion trend of high growth rate areas is not only linear due to parabolic and inverse parabolic trends have been described in other brachiopod shells [51]. These variations at species or genera level related with the specific growth rate or metabolism of the brachiopods which can modify the incorporation of ions and isotopes are called `vital effects`. Some authors were concerned about its influence in the shell geochemistry (*e.g.*, Mg/Ca, $\delta^{13}C$ and $\delta^{18}O$) and the possible paleoclimatological misinterpretations [50]. According to McConnaughey [105], 'vital effects' supposes a noteworthy problem for isotopic geochemistry and paleoclimatology studies. The current results on *Gigantoproductus* geochemical variation underline that can be extended to the fossil record and more specifically to the interpretation of the Late Paleozoic Ice Age (LPIA). This requires the identification of 'ideal' sampling regions in order to avoid differences in ion partitioning and isotope fractionation related to the differences in the growth rates (kinetic effects).

Gigantoproductus regions less affected by "vital effects" (*sensu* "equilibrium zone" or "plateau zone" by Perez-Huerta et al. [55] and Rollion-Bard et al. [43]) are the T- and the inner zone of Tk-regions (Figures 7-8), because the low dispersion of the data. It should be discarded the umbonal region and external parts of Tk-region of *Gigantoproductus* ventral valves to avoid paleoclimatological misinterpretations (up to ~1.05 ‰ of $\delta^{18}O$ and 14 mmol/mol of Mg/Ca). Herein, *Gigantoproductus* tertiary layer concentrates a great potential for paleoclimatological studies due to i) higher volume of shell substance compared with the secondary layer; ii) growth rate variability, which allows characterizing the 'vital effects' of the shell; iii) less prone to diagenetic alteration than the secondary layer; 4) higher growth lines spacing, which allows to avoid growth lines.

5. Conclusions

A comprehensive study of the microstructure, crystallography, and geochemistry (trace elements, $\delta^{18}O$, $\delta^{13}C$ and $^{87}Sr/^{86}Sr$) of tertiary layer of *Gigantoproductus* sp. ventral valves has allowed the differentiation of areas with signs of diagenesis, from primary biogenic features. In addition of fossil preservation, a special emphasis has been taken in the characterisation of the geochemical 'vital effects'.

Gigantoproductus shells consist in three layers, but only two are still preserved: the secondary layer with laminar microstructure, and the tertiary layer with a characteristic columnar microstructure.

Evidence of diagenesis have been recognised in some poorly-preserved areas because of the original crystallo-chemical features have been obliterated. These areas regardless of luminescence under CL (*i.e.*, NL or SL) show neomorphism or recrystallisation s.l., a random crystallographic distribution, and disperse geochemical composition. It should be highlighted that the luminescence under fluorescence microscopy, identified in some well-preserved areas as growth-lines, corresponds mainly to preserved organic matrix remains.

Well-preserved areas of secondary and tertiary layers exhibit a well-constrained crystallographic arrangement, with the *c*-axis parallel oriented to the elongation axis of the prisms and perpendicular to the laminae. Three structural regions have been identified in the ventral valve based on the curvature and thickness changes of ventral valve: the umbonal (U), thick (Tk) and thin (T) regions.

Furthermore, the geochemical composition of these areas is well-constrained in MTE (Ca, Mg, Sr, Na, S, Mn, and Fe) as well as isotope composition ($\delta^{13}C$, $\delta^{18}O$ and $^{87}Sr/^{86}Sr$), with a characteristic variability possibly related with shell growth rate differences, identifying them as geochemical 'vital effects'. Younger regions with higher growth rates (*e.g.*, umbonal and secondary - tertiary layer interphase) are Mg-, S-, Na-enriched and $\delta^{13}C$ and $\delta^{18}O$-depleted, whereas older zones (inner zones of Tk- and T-regions) are Mg-, S-, Na-impoverished and $\delta^{13}C$ and $\delta^{18}O$ -enriched. These last regions are the most suitable areas for geochemical analysis in *Gigantoproductus*.

The 'vital effects' caused by kinetic effects has a direct influence in the paleotemperature estimation, until ~1.05‰ of $\delta^{18}O$ and 14 mmol/mol of Mg/Ca, intra-shell variation was detected, which can modify the ulterior paleoclimatological interpretation, underestimating or overestimating the environmental conditions of the LPIA.

Supplementary Materials: The following supporting information can be downloaded at: www.mdpi.com/xxx/s1, Figure S1-S4.

Author Contributions: Conceptualization, I.C., S.R., and J.R.MC.; methodology, J.R.MC., J.A.C., and IC.; investigation, J.R.M.C., I.C. J.A.C., P.C, E.F.M. and S.R.; writing—original draft preparation, I.C., J.R.M.C.; writing—review and editing, J.R.M.C., I.C. J.A.C., P.C, E.F.M. and S.R.; visualization, .R.MC. and IC.; supervision, I.C., P.C. S.R.. All authors have read and agreed to the published version of the manuscript.

Funding: This research was funded by Spanish Ministerio de Economía y Competitividad (research project CGL2016-78738-P).

Institutional Review Board Statement: Not applicable.

Informed Consent Statement: Not applicable.
Data Availability Statement: All data are included in figures and tables of this article.

Acknowledgments: Financial support through the Spanish Ministerio de Economía y Competitividad (research project CGL2016-78738-P) and the Complutense University Research Group (910231) is gratefully acknowledged. J.R.M.C. acknowledges financial support through an FPI-MINECO grant. This article is a contribution to the Spanish Working Group IGCP 596 (UNESCO).

Conflicts of Interest: The authors declare no conflict of interest.

References

1. Isbell, J.L.; Henry, L.C.; Gulbranson, E.L.; Limarino, C.O.; Fraiser, M.L.; Koch, Z.J.; Ciccioli, P.L.; Dineen, A.A. Glacial Paradoxes during the Late Paleozoic Ice Age: Evaluating the Equilibrium Line Altitude as a Control on Glaciation. *Gondwana Res.* **2012**, *22*, 1–19, doi:https://doi.org/10.1016/j.gr.2011.11.005.
2. Saltzman, M.R. Late Paleozoic Ice Age: Oceanic Gateway or *p*CO_2? *Geology* **2003**, *31*, 151–154, doi:10.1130/0091-7613(2003) 031<0151:LPIAOG>2.0.CO;2.
3. Powell, M.G. Climatic Basis for Sluggish Macroevolution during the Late Paleozoic Ice Age. *Geology* **2005**, *33*, 381–384, doi:10.1130/G21155.1.
4. Fielding, C.R.; Frank, T.D.; Birgenheier, L.P.; Rygel, M.C.; Jones, A.T.; Roberts, J. Stratigraphic Imprint of the Late Palaeozoic Ice Age in Eastern Australia: A Record of Alternating Glacial and Nonglacial Climate Regime. *J. Geol. Soc. London.* **2008**, *165*, 129–140, doi:10.1144/0016-76492007-036.
5. Shi, G.R.; Waterhouse, J.B. Late Palaeozoic Global Changes Affecting High-Latitude Environments and Biotas: An Introduction. *Palaeogeogr. Palaeoclimatol. Palaeoecol.* **2010**, *298*, 1–16, doi:https://doi.org/10.1016/j.palaeo.2010.07.021.
6. Montañez, I.P.; Poulsen, C.J. The Late Paleozoic Ice Age: An Evolving Paradigm. *Annu. Rev. Earth Planet. Sci.* **2013**, *41*, 629–656, doi:10.1146/annurev.earth.031208.100118.
7. Qiao, L.; Shen, S. Global Paleobiogeography of Brachiopods during the Mississippian—Response to the Global Tectonic Reconfiguration, Ocean Circulation, and Climate Changes. *Gondwana Res.* **2014**, *26*, 1173–1185, doi:https://doi.org/10.1016/j.gr.2013.09.013.
8. Goddéris, Y.; Donnadieu, Y.; Carretier, S.; Aretz, M.; Dera, G.; Macouin, M.; Regard, V. Onset and Ending of the Late Palaeozoic Ice Age Triggered by Tectonically Paced Rock Weathering. *Nat. Geosci.* **2017**, *10*, 382–386, doi:10.1038/ngeo2931.
9. Armendáriz, M.; Rosales, I.; Quesada, C. Oxygen Isotope and Mg/Ca Composition of Late Viséan (Mississippian) Brachiopod Shells from SW Iberia: Palaeoclimatic and Palaeogeographic Implications in Northern Gondwana. *Palaeogeogr. Palaeoclimatol. Palaeoecol.* **2008**, *268*, 65–79, doi:10.1016/j.palaeo.2008.07.008.
10. McGhee, G.R.; Sheehan, P.M.; Bottjer, D.J.; Droser, M.L. Ecological Ranking of Phanerozoic Biodiversity Crises: The Serpukhovian (Early Carboniferous) Crisis Had a Greater Ecological Impact than the End-Ordovician. *Geology* **2012**, *40*, 147–150, doi:10.1130/g32679.1.
11. Stanley, S.M.; Powell, M.G. Depressed Rates of Origination and Extinction during the Late Paleozoic Ice Age: A New State for the Global Marine Ecosystem. *Geology* **2003**, *31*, 877–880, doi:10.1130/g19654r.1.
12. Brand, U.W.E. The Oxygen and Carbon Isotope Composition of Carboniferous Fossil Components: Sea-Water Effects. *Sedimentology* **1982**, *29*, 139–147, doi:https://doi.org/10.1111/j.1365-3091.1982.tb01715.x.
13. Grossman, E.L.; Yancey, T.E.; Jones, T.E.; Bruckschen, P.; Chuvashov, B.; Mazzullo, S.J.; Mii, H. sheng Glaciation, Aridification, and Carbon Sequestration in the Permo-Carboniferous: The Isotopic Record from Low Latitudes. *Palaeogeogr. Palaeoclimatol. Palaeoecol.* **2008**, *268*, 222–233, doi:10.1016/j.palaeo.2008.03.053.
14. Stephenson, M.H.; Angiolini, L.; Cózar, P.; Jadoul, F.; Leng, M.J.; Millward, D.; Chenery, S. Northern England Serpukhovian (Early Namurian) Farfield Responses to Southern Hemisphere Glaciation. *J. Geol. Soc. London.* **2010**, *167*, 1171–1184, doi:10.1144/0016-76492010-048.
15. Brand, U.; Jiang, G.; Azmy, K.; Bishop, J.; Montañez, I.P. Diagenetic Evaluation of a Pennsylvanian Carbonate Succession (Bird Spring Formation, Arrow Canyon, Nevada, U.S.A.) — 1: Brachiopod and Whole Rock Comparison. *Chem. Geol.* **2012**, *308–309*, 26–39, doi:https://doi.org/10.1016/j.chemgeo.2012.03.017.
16. Roark, A.; Grossman, E.L.; Lebold, J. Low Seasonality in Central Equatorial Pangea during a Late Carboniferous Highstand Based on High-Resolution Isotopic Records of Brachiopod Shells. *Bull. Geol. Soc. Am.* **2016**, *128*, 597–608, doi:10.1130/B31330.1.
17. Popp Anderson, T. F., Sandberg, P. A., B.N. Brachiopods as Indicators of Original Isotopic Compositions in Some Paleozoic Limestones. *Geol. Soc. Am. Bull.* **1986**, *97*, 1262–1269, doi:10.1130/0016-7606(1986)97<1262:baiooi>2.0.co;2.

18. Banner, J.L.; Kaufman, J. The Isotopic Record of Ocean Chemistry and Diagenesis Preserved in Non-Luminescent Brachiopods from Mississippian Carbonate Rocks, Illinois and Missouri. *Geol. Soc. Am. Bull.* **1994**, *106*, 1074–1082, doi:10.1130/0016-7606(1994)106<1074:TIROOC>2.3.CO;2.
19. Bruckschen, P.; Bruhn, F.; Veizer, J.; Buhl, D. 87Sr86Sr Isotopic Evolution of Lower Carboniferous Seawater: Dinantian of Western Europe. *Sediment. Geol.* **1995**, *100*, 63–81, doi:10.1016/0037-0738(95)00103-4.
20. Bruckschen, P.; Oesmann, S.; Veizer, J. Isotope Stratigraphy of the European Carboniferous: Proxy Signals for Ocean Chemistry, Climate and Tectonics. *Chem. Geol.* **1999**, *161*, 127–163, doi:10.1016/S0009-2541(99)00084-4.
21. Bruckschen, P.; Veizer, J. Oxygen and Carbon Isotopic Composition of Dinantian Brachiopods: Paleoenvironmental Implications for the Lower Carboniferous of Western Europe. *Palaeogeogr. Palaeoclimatol. Palaeoecol.* **1997**, *132*, 243–264, doi:10.1016/S0031-0182(97)00066-7.
22. Mii, H.S.; Grossman, E.L.; Yancey, T.E. Carboniferous Isotope Stratigraphies of North America: Implications for Carboniferous Paleoceanography and Mississippian Glaciation. *Bull. Geol. Soc. Am.* **1999**, *111*, 960–973, doi:10.1130/0016-7606(1999)111<0960:CISONA>2.3.CO;2.
23. Grossman, E.L.; Bruckschen, P.; Mii, H.-S.; Chuvashov, B.I.; Yancey, T.E.; Veizer, J. Carboniferous Paleoclimate and Global Change: Isotopic Evidence from the Russian Platform. *Carbonif. Stratigr. Paleogeography Eurasia* **2002**, 61–71.
24. Batt, L.S.; Montañez, I.P.; Isaacson, P.; Pope, M.C.; Butts, S.H.; Abplanalp, J. Multi-Carbonate Component Reconstruction of Mid-Carboniferous (Chesterian) Seawater Δ13C. *Palaeogeogr. Palaeoclimatol. Palaeoecol.* **2007**, *256*, 298–318, doi:10.1016/j.palaeo.2007.02.049.
25. Brand, U. Biogeochemistry of Late Paleozoic North American Brachiopods and Secular Variation of Seawater Composition. *Biogeochemistry* **1989**, *7*, 159–193, doi:10.1007/BF00004216.
26. Veizer, J.; Fritz, P.; Jones, B. Geochemistry of Brachiopods: Oxygen and Carbon Isotopic Records of Paleozoic Oceans. *Geochim. Cosmochim. Acta* **1986**, *50*, 1679–1696, doi:10.1016/0016-7037(86)90130-4.
27. Veizer, J.; Bruckschen, P.; Pawellek, F.; Diener, A.; Podlaha, O.G.; Carden, G.A.F.; Jasper, T.; Korte, C.; Strauss, H.; Azmy, K.; et al. Oxygen Isotope Evolution of Phanerozoic Seawater. *Palaeogeogr. Palaeoclimatol. Palaeoecol.* **1997**, *132*, 159–172, doi:10.1016/S0031-0182(97)00052-7.
28. Mii, H.S.; Grossman, E.L.; Yancey, T.E.; Chuvashov, B.; Egorov, A. Isotopic Records of Brachiopod Shells from the Russian Platform - Evidence for the Onset of Mid-Carboniferous Glaciation. *Chem. Geol.* **2001**, *175*, 133–147, doi:Doi 10.1016/S0009-2541(00)00366-1.
29. Pérez-Huerta, A.; Coronado, I.; Hegna, T.A. Understanding Biomineralization in the Fossil Record. *Earth-Science Rev.* **2018**, *179*, 95–122, doi:10.1016/j.earscirev.2018.02.015.
30. Muir-Wood, H.; Cooper, G.A. *Morphology, Classification and Life Habits of the Productoidea (Brachiopoda)*; Geological Society of America, 1960; ISBN 9780813710815.
31. Ferguson, J. Some Aspects of the Ecology and Growth of the Carboniferous Gigantoproductids. *Proc. Yorksh. Geol. Soc.* **1978**, *42*, 41–54, doi:10.1144/pygs.42.1.41.
32. Nolan, L.S.P.; Angiolini, L.; Jadoul, F.; Della Porta, G.; Davies, S.J.; Banks, V.J.; Stephenson, M.H.; Leng, M.J. Sedimentary Context and Palaeoecology of Gigantoproductus Shell Beds in the Mississippian Eyam Limestone Formation, Derbyshire Carbonate Platform, Central England. *Proc. Yorksh. Geol. Soc.*, doi:10.1144/pygs2017-393.
33. Brunton, C.H.C.; Lazarev, S.S.; Grant, R.E. A Review and New Classification of the Brachiopod Order Productida. *Palaeontology* **1995**, *38*, 915–936.
34. Angiolini, L.; Stephenson, M.; Leng, M.J.; Jadoul, F.; Millward, D.; Aldridge, A.; Andrews, J.; Chenery, S.; Williams, G. Heterogeneity, Cyclicity and Diagenesis in a Mississippian Brachiopod Shell of Palaeoequatorial Britain. *Terra Nov.* **2012**, *24*, 16–26, doi:10.1111/j.1365-3121.2011.01032.x.
35. Angiolini, L.; Crippa, G.; Azmy, K.; Capitani, G.; Confalonieri, G.; Della Porta, G.; Griesshaber, E.; Harper, D.A.T.; Leng, M.J.; Nolan, L.; et al. The Giants of the Phylum Brachiopoda: A Matter of Diet? *Palaeontology* **2019**, *62*, 889–917, doi:https://doi.org/10.1111/pala.12433.
36. Grossman, E.L.; Mii, H.-S.; Zhang, C.; Yancey, T.E. Chemical Variation in Pennsylvanian Brachiopod Shells; Diagenetic, Taxonomic, Microstructural, and Seasonal Effects. *J. Sediment. Res.* **1996**, *66*, 1011–1022, doi:10.1306/d4268469-2b26-11d7-8648000102c1865d.
37. Rush, P.F.; Chafetz, H. Fabric-Retentive, Non-Luminescent Brachiopods as Indicators of Original ?? 13C and ?? 18O Composition: A Test. *J. Sediment. Petrol.* **1990**, *60*, 968–981, doi:10.1306/D4267659-2B26-11D7-8648000102C1865D.
38. Pérez-Huerta, A.; Cusack, M.; Janousch, M.; Finch, A.A. Influence of Crystallographic Orientation of Biogenic Calcite on in Situ Mg XANES Analyses. *J. Synchrotron Radiat.* **2008**, *15*, 572–575, doi:10.1107/S0909049508026484.
39. Urey, H.C.; Lowenstam, H. a.; Epstein, S.; McKinney, C.R. Measurement of Paleotemperatures and Temperatures and the Southeastern United States. *Bull. Geol. Soc. Am.* **1951**, *62*, 399–416, doi:10.1130/0016-7606(1951)62[399:MOPATO]2.0.CO;2.
40. Carpenter, S.J.; Lohmann, K.C. Δ18O and Δ13C Values of Modern Brachiopod Shells. *Geochim. Cosmochim. Acta* **1995**, *59*, doi:10.1016/0016-7037(95)00291-7.

41. Brand, U.; Azmy, K.; Griesshaber, E.; Bitner, M.A.; Logan, A.; Zuschin, M.; Ruggiero, E.; Colin, P.L. Carbon Isotope Composition in Modern Brachiopod Calcite: A Case of Equilibrium with Seawater? *Chem. Geol.* **2015**, *411*, 81–96, doi:https://doi.org/10.1016/j.chemgeo.2015.06.021.
42. Rollion-Bard, C.; Saulnier, S.; Vigier, N.; Schumacher, A.; Chaussidon, M.; Lécuyer, C. Variability in Magnesium, Carbon and Oxygen Isotope Compositions of Brachiopod Shells: Implications for Paleoceanographic Studies. *Chem. Geol.* **2016**, *423*, 49–60, doi:10.1016/j.chemgeo.2016.01.007.
43. Rollion-Bard, C.; Milner Garcia, S.; Burckel, P.; Angiolini, L.; Jurikova, H.; Tomašových, A.; Henkel, D. Assessing the Biomineralization Processes in the Shell Layers of Modern Brachiopods from Oxygen Isotopic Composition and Elemental Ratios: Implications for Their Use as Paleoenvironmental Proxies. *Chem. Geol.* **2019**, *524*, 49–66, doi:https://doi.org/10.1016/j.chemgeo.2019.05.031.
44. Riechelmann, S.; Mavromatis, V.; Buhl, D.; Dietzel, M.; Eisenhauer, A.; Immenhauser, A. Impact of Diagenetic Alteration on Brachiopod Shell Magnesium Isotope (Δ26Mg) Signatures: Experimental versus Field Data. *Chem. Geol.* **2016**, *440*, 191–206, doi:http://dx.doi.org/10.1016/j.chemgeo.2016.07.020.
45. Pérez-Huerta, A.; C. Fred, T.A.; Andrus, F.T. Vital Effects in the Context of Biomineralization. In *Workshop on Biominerals and Biomineralization Processes*; Fernández-Díaz, L., Astilleros, J.M., Eds.; Sociedad Española de Mienralogía: Madrid, 2010; Vol. 7, pp. 35–45.
46. Weiner, S.; Dove, P.M. An Overview of Biomineralization Processes and the Problem of the Vital Effect. *Rev. Mineral. Geochemistry* **2003**, *54*, 1–29, doi:10.2113/0540001.
47. De Yoreo, J.J.; Gilbert, P.U.P.A.P.A.; Sommerdijk, N.A.J.M.J.M.; Penn, R.L.; Whitelam, S.; Joester, D.; Zhang, H.; Rimer, J.D.; Navrotsky, A.; Banfield, J.F.; et al. Crystallization by Particle Attachment in Synthetic, Biogenic, and Geologic Environments. *Science (80-.).* **2015**, *349*, aaa6760, doi:10.1126/science.aaa6760.
48. Coronado, I.; Fine, M.; Bosellini, F.R.F.R.; Stolarski, J. Impact of Ocean Acidification on Crystallographic Vital Effect of the Coral Skeleton. *Nat. Commun.* **2019**, *10*, 1–9, doi:10.1038/s41467-019-10833-6.
49. Auclair, A.-C.; Joachimski, M.M.; Lécuyer, C. Deciphering Kinetic, Metabolic and Environmental Controls on Stable Isotope Fractionations between Seawater and the Shell of Terebratalia Transversa (Brachiopoda). *Chem. Geol.* **2003**, *202*, 59–78, doi:https://doi.org/10.1016/S0009-2541(03)00233-X.
50. Parkinson, D.; Curry, G.B.; Cusack, M.; Fallick, A.E. Shell Structure, Patterns and Trends of Oxygen and Carbon Stable Isotopes in Modern Brachiopod Shells. *Chem. Geol.* **2005**, *219*, 193–235, doi:https://doi.org/10.1016/j.chemgeo.2005.02.002.
51. Romanin, M.; Crippa, G.; Ye, F.; Brand, U.; Bitner, M.; Gaspard, D.; Häussermann, V.; Laudien, J. A Sampling Strategy for Recent and Fossil Brachiopods: Selecting the Optimal Shell Segment for Geochemical Analyses. *Riv. Ital. di Paleontol. e Stratigr.* **2018**, *124*, doi:10.13130/2039-4942/10193.
52. Bajnai, D.; Fiebig, J.; Tomašových, A.; Milner Garcia, S.; Rollion-Bard, C.; Raddatz, J.; Löffler, N.; Primo-Ramos, C.; Brand, U. Assessing Kinetic Fractionation in Brachiopod Calcite Using Clumped Isotopes. *Sci. Rep.* **2018**, *8*, 533, doi:10.1038/s41598-017-17353-7.
53. Brand, U.; Logan, A.; Hiller, N.; Richardson, J. Geochemistry of Modern Brachiopods: Applications and Implications for Oceanography and Paleoceanography. *Chem. Geol.* **2003**, *198*, 305–334, doi:https://doi.org/10.1016/S0009-2541(03)00032-9.
54. Cusack, M.; Pérez-Huerta, A.; Janousch, M.; Finch, A.A. Magnesium in the Lattice of Calcite-Shelled Brachiopods. *Chem. Geol.* **2008**, *257*, 59–64, doi:10.1016/j.chemgeo.2008.08.007.
55. Pérez-Huerta, A.; Cusack, M.; Jeffries, T.E.; Williams, C.T. High Resolution Distribution of Magnesium and Strontium and the Evaluation of Mg/Ca Thermometry in Recent Brachiopod Shells. *Chem. Geol.* **2008**, *247*, 229–241, doi:10.1016/j.chemgeo.2007.10.014.
56. Buening, N.; Carlson, S.J. Geochemical Investigation of Growth in Selected Recent Articulate Brachiopods. *Lethaia* **1992**, *25*, 331–345, doi:https://doi.org/10.1111/j.1502-3931.1992.tb01402.x.
57. Brand, U.; Azmy, K.; Bitner, M.A.; Logan, A.; Zuschin, M.; Came, R.; Ruggiero, E. Oxygen Isotopes and MgCO3 in Brachiopod Calcite and a New Paleotemperature Equation. *Chem. Geol.* **2013**, *359*, 23–31, doi:https://doi.org/10.1016/j.chemgeo.2013.09.014.
58. Brand, U.; Bitner, M.A.; Logan, A.; Azmy, K.; Crippa, G.; Angiolini, L.; Colin, P.; Griesshaber, E.; Harper, E.; Haussermann, V. Brachiopod-Based Oxygen-Isotope Thermometer: Update and Review. *Riv. Ital. di Paleontol. e Stratigr.* **2019**, *125*, 775–778.
59. Cózar, P.; Rodríguez, S. Propuesta de nueva nomenclatura para las unidades del Carbonífero Inferior del sector norte del área del Guadiato (Córdoba) . *Boletín Geológico y Min.* **1999**, *110*, 237–254.
60. Cózar, P.; Rodríguez, S. Pendleian (Early Serpukhovian) Marine Carbonates from SW Spain: Sedimentology, Biostratigraphy and Depositional Model. *Geol. J.* **2004**, *39*, 25–47, doi:10.1002/gj.942.
61. Medina-Varea, P.; Sarmiento, G.; Rodríguez, S.; Cózar, P. Early Serpukhovian Conodonts from the Guadiato Area (Córdoba, Spain). **2005**, *55*, 21–50.
62. Cózar, P.; Rodríguez, S.; Somerville, I.D. Large Multi-Biotic Cyanoliths from Relatively Deep-Water Facies in the Early Serpukhovian of SW Spain. *Facies* **2003**, *49*, 31–48, doi:10.1007/s10347-003-0023-0.
63. Cózar, P. Bioestratigrafía Con Foraminíferos Del Carbonífero Inferior Del Sector Norte Del Área Del Guadiato (Córdoba), Universidad Complutense, 1998.

64. Cózar, P.; Rodríguez, S. Pendleian (Early Serpukhovian) Marine Carbonates from SW Spain: Sedimentology, Biostratigraphy and Depositional Model. *Geol. J.* **2004**, *39*, 25–47.
65. Coronado, I.; Pérez-Huerta, A.; Rodríguez, S. Primary Biogenic Skeletal Structures in Multithecopora (Tabulata, Pennsylvanian). *Palaeogeogr. Palaeoclimatol. Palaeoecol.* **2013**, *386*, 286–299, doi:http://dx.doi.org/10.1016/j.palaeo.2013.05.030.
66. Coronado, I.; Pérez-Huerta, A.; Rodríguez, S. Computer-Integrated Polarisation (CIP) in the Analysis of Fossils: A Case of Study in a Palaeozoic Coral (Sinopora, Syringoporicae, Carboniferous). *Hist. Biol.* **2015**, *27*, 1098–1112, doi:10.1080/08912963.2014.938236.
67. Coronado, I.; Fernández-Martínez, E.; Rodríguez, S.; Tourneur, F. Reconstructing a Carboniferous Inferred Coral–Alcyonarian Association Using a Biomineralogical Approach. *Geobiology* **2015**, *13*, 340–356, doi:10.1111/gbi.12133.
68. Coronado, I.; Rodríguez, S. Biomineral Structure and Crystallographic Arrangement of Cerioid and Phaceloid Growth in Corals Belonging to the Syringoporicae (Tabulata, Devonian–Carboniferous): A Genetic Reflection. *Geol. Mag.* **2016**, *FirstView*, 1–25, doi:10.1017/S0016756815000862.
69. Coronado, I.; Rodríguez, S. Microstructure and Crystallography in Axophyllinae. Precisions on the Genus Morenaphyllum. *Spanish J. Palaeontol.* **2018**, *33*, 41, doi:10.7203/sjp.33.1.13241.
70. Barret, S. Image Analysis and the Internet . *Sci. Data Manag.* **1997**, *1*, 18–25.
71. Vischer, N.O.E.; Huls, P.G.; Woldringh, C.L. Object-Image - an Interactive Image-Analysis Program Using Structured Point Collection. *Bin. Microbiol.* **1994**, *6*, 160–166, doi:urn:nbn:nl:ui:29-4252.
72. Abràmofff, M.D.; Magalhães, P.J.; Ram, S.J. Image Processing with ImageJ Part II. *Biophotonics Int.* **2005**, *11*, 36–43.
73. Garbelli, C.; Angiolini, L..; Shen, S.-Z.. Biomineralization and Global Change: A New Perspective for Understanding the End-Permian Extinction. *Geology* **2017**, *45*, 19–22, doi:10.1130/g38430.1.
74. Garbelli, C.; Angiolini, L.; Jadoul, F.; Brand, U. Micromorphology and Differential Preservation of Upper Permian Brachiopod Low-Mg Calcite. *Chem. Geol.* **2012**, *298*, 1–10, doi:http://dx.doi.org/10.1016/j.chemgeo.2011.12.019.
75. Griesshaber, E.; Schmahl, W.W.; Neuser, R.; Pettke, T.; Blüm, M.; Mutterlose, J.; Brand, U. Crystallographic Texture and Microstructure of Terebratulide Brachiopod Shell Calcite: An Optimized Materials Design with Hierarchical Architecture. *Am. Mineral.* **2007**, *92*, 722–734, doi:10.2138/am.2007.2220.
76. Casella, L.A.; He, S.; Griesshaber, E.; Fernández-Díaz, L.; Greiner, M.; Harper, E.M.; Jackson, D.J.; Ziegler, A.; Mavromatis, V.; Dietzel, M.; et al. Hydrothermal Alteration of Aragonitic Biocarbonates: Assessment of Micro- and Nanostructural Dissolution-Reprecipitation and Constraints of Diagenetic Overprint from Quantitative Statistical Grain-Area Analysis. *Biogeosciences* **2018**, *15*, 7451–7484, doi:10.5194/bg-15-7451-2018.
77. Cusack, M.; Parkinson, D.; Pérez-Huerta, A.; England, J.; Curry, G.B.; Fallick, A.E. Relationship between Δ18O and Minor Element Composition of Terebratalia Transversa. *Earth Environ. Sci. Trans. R. Soc. Edinburgh* **2007**, *98*, 443–449, doi:10.1017/S1755691008075671.
78. PÉREZ-HUERTA, A.; REED, H. Preliminary Assessment of Coupling the Analysis of Shell Microstructures and Microtextures as Palaeoecological Indicator in Fossil Brachiopods. *Spanish J. Palaeontol.* **2018**, *33*, 129, doi:10.7203/sjp.33.1.13246.
79. Goetz, A.J.; Griesshaber, E.; Neuser, R.D.L.; Harper, E.; Schmahl, W.W. Calcite Morphology, Texture and Hardness in the Distinct Layers of Rhynchonelliform Brachiopod Shells. *Eur. J. Mineral.* **2009**, *21*, 303–315, doi:10.1127/0935-1221/2009/0021-1922.
80. Schmahl, W.W. The Hierarchical Organization in Biomaterials: From Nanoparticles via Mesocrystals to Functionality Citation. In *Workshop on Biominerals and Biomineralization Processes*; Fernández Díaz, L., Astilleros García-Monge, J.M., Eds.; Sociedad Española de Mineralogía: Madrid, 2010; Vol. 7, pp. 5–21.
81. Gilbert, P.U.P.A.; Porter, S.M.; Sun, C.Y.; Xiao, S.; Gibson, B.M.; Shenkar, N.; Knoll, A.H. Biomineralization by Particle Attachment in Early Animals. *Proc. Natl. Acad. Sci. U. S. A.* **2019**, *116*, doi:10.1073/pnas.1902273116.
82. Cusack, M.; Dauphin, Y.; Chung, P.; Pérez-Huerta, A.; Cuif, J.P. Multiscale Structure of Calcite Fibres of the Shell of the Brachiopod Terebratulina Retusa. *J. Struct. Biol.* **2008**, *164*, 96–100, doi:10.1016/j.jsb.2008.06.010.
83. Cusack, M.; Perez-Huerta, A.; Chung, P.; Parkinson, D.; Dauphin, Y.; Cuif, J.-P.P.; A. Pérez-Huerta; Chung, P.; Parkinson, D.; Dauphin, Y.; et al. Oxygen Isotope Equilibrium in Brachiopod Shell Fibres in the Context of Biological Control. *Mineral. Mag.* **2008**, *72*, 239–242, doi:10.1180/minmag.2008.072.1.239.
84. Grossman, E.L.; Mii, H.S.; Yancey, T.E. Stable Isotopes in Late Pennsylvanian Brachiopods from the United-States - Implications for Carboniferous Paleoceanography. *Geol. Soc. Am. Bull.* **1993**, *105*, 1284–1296, doi:Doi 10.1130/0016-7606(1993)105<1284:Siilpb>2.3.Co;2.
85. Long, J.V.P.; Agrell, S.O. The Cathodo-Luminescence of Minerals in Thin Section. *Mineral. Mag. J. Mineral. Soc.* **1965**, *34*, 318–326, doi:DOI: 10.1180/minmag.1965.034.268.27.
86. Frank, J.R.; Carpenter, A.B.; Oglesby, T.W. Cathodoluminescence and Composition of Calcite Cement in the Taum Sauk Limestone (Upper Cambrian), Southeast Missouri. *J. Sediment. Res.* **1982**, *52*, 631–638, doi:10.1306/212F7FB8-2B24-11D7-8648000102C1865D.
87. Machel, H.-G. Cathodoluminescence in Calcite and Dolomite and Its Chemical Interpretation. *Geosci. Canada* **1985**, *12*.
88. Boggs, S.; Krinsley, D. *Application of Cathodoluminescence Imaging to the Study of Sedimentary Rocks*; Cambridge University Press, 2006;

89. Barbin, V.; Gaspard, D. Cathodoluminescence of Recentarticulate Brachiopod Shells. Implications for Growth Stages and Diagenesis Evaluation. *Geobios* **1995**, *28*, 39–45, doi:10.1016/S0016-6995(95)80151-0.
90. Barbin, V. Cathodoluminescence of Carbonate Shells: Biochemical vs Diagenetic Process. In *Cathodoluminescence in Geosciences*; Pagel, M., Barbin, V., Blanc, P., Ohnenstetter, D., Eds.; Springer Berlin Heidelberg, 2000; pp. 303–329 ISBN 978-3-642-08526-0.
91. Barbin, V. Application of Cathodoluminescence Microscopy to Recent and Past Biological Materials: A Decade of Progress. *Mineral. Petrol.* **2013**, *107*, 353–362, doi:10.1007/s00710-013-0266-6.
92. Tomašových, A.; Farkaš, J. Cathodoluminescence of Late Triassic Terebratulid Brachiopods: Implications for Growth Patterns. *Palaeogeogr. Palaeoclimatol. Palaeoecol.* **2005**, *216*, 215–233, doi:http://dx.doi.org/10.1016/j.palaeo.2004.11.010.
93. Benito, M.I.; Reolid, M. Belemnite Taphonomy (Upper Jurassic, Western Tethys) Part II: Fossil-Diagenetic Analysis Including Combined Petrographic and Geochemical Techniques. *Palaeogeogr. Palaeoclimatol. Palaeoecol.* **2012**, *358–360*, 89–108, doi:10.1016/j.palaeo.2012.06.035.
94. Lutz, R.A.; Rhoads, D.C. Anaerobiosis and a Theory of Growth Line Formation. *Science (80-.).* **1977**, *198*, 1222–1227.
95. Hughes, W.W.; Rosenberg, G.D.; Tkachuck, R.D. Growth Increments in the Shell of the Living Brachiopod Terebratalia Transversa. *Mar. Biol.* **1988**, *98*, 511–518, doi:10.1007/BF00391542.
96. Curry, G. Shell Growth and Ecology of the Recent Brachiopod Terebratulina from Scotland. *Palaeontology* **1982**, *25*, 227–246.
97. Müller, T.; Tomašových, A.; Correa, M.L.; Mertz-Kraus, R.; Mikuš, T. Mapping Intrashell Variation in Mg/Ca of Brachiopods to External Growth Lines: Mg Enrichment Corresponds to Seasonal Growth Slowdown. *Chem. Geol.* **2022**, *593*, 120758, doi:https://doi.org/10.1016/j.chemgeo.2022.120758.
98. Busenberg, E.; Niel Plummer, L. Kinetic and Thermodynamic Factors Controlling the Distribution of SO32− and Na+ in Calcites and Selected Aragonites. *Geochim. Cosmochim. Acta* **1985**, *49*, 713–725, doi:https://doi.org/10.1016/0016-7037(85)90166-8.
99. Rollion-Bard, C.; Blamart, D. Possible Controls on Li, Na, and Mg Incorporation into Aragonite Coral Skeletons. *Chem. Geol.* **2015**, *396*, 98–111, doi:https://doi.org/10.1016/j.chemgeo.2014.12.011.
100. Ishikawa, M.; Ichikuni, M. Uptake of Sodium and Potassium by Calcite. *Chem. Geol.* **1984**, *42*, 137–146, doi:https://doi.org/10.1016/0009-2541(84)90010-X.
101. Yoshimura, T.; Suzuki, A.; Tamenori, Y.; Kawahata, H. Micro-X-Ray Fluorescence-Based Comparison of Skeletal Structure and P, Mg, Sr, O and Fe in a Fossil of the Cold-Water Coral Desmophyllum Sp., NW Pacific. *Geo-Marine Lett.* **2014**, *34*, 1–9, doi:10.1007/s00367-013-0347-x.
102. Yoshimura, T.; Tamenori, Y.; Suzuki, A.; Kawahata, H.; Iwasaki, N.; Hasegawa, H.; Nguyen, L.T.; Kuroyanagi, A.; Yamazaki, T.; Kuroda, J.; et al. Altervalent Substitution of Sodium for Calcium in Biogenic Calcite and Aragonite. *Geochim. Cosmochim. Acta* **2017**, *202*, 21–38, doi:10.1016/j.gca.2016.12.003.
103. Mii, H.-S.; Grossman, E.L. Late Pennsylvanian Seasonality Reflected in the 18O and Elemental Composition of a Brachiopod Shell. *Geology* **1994**, *22*, 661, doi:10.1130/0091-7613(1994)022<0661:lpsrit>2.3.co;2.
104. Mucci, A. The Solubility of Calcite and Aragonite in Seawater at Various Salinities, Temperatures, and One Atmosphere Tot Al Pressure. *Am. J. Sci.* **1983**, *283*, 780–799.
105. McConnaughey, T. 13C and 18O Isotopic Disequilibrium in Biological Carbonates: I. Patterns. *Geochim. Cosmochim. Acta* **1989**, *53*, 151–162, doi:https://doi.org/10.1016/0016-7037(89)90282-2.
