# Supplementary material for: Diagenetic and Biological Overprints in Geochemical Signatures of the Gigantoproductus Tertiary Layer (Brachiopoda): Assessing the Paleoclimatic Interpretation
Source: Life (Basel). 2023 Mar 6;13(3):714. doi: 10.3390/life13030714 (PMC10057505; doi:10.3390/life13030714)
Supplement: Supplementary file 1 [file life-13-00714-s001.zip › 5. Mateos-Carralafuente et al. Supplementary Material.pdf]

Diagenetic and biological overprints in geochemical signatures of *Gigantoproductus* tertiary layer (Brachiopoda): assessing the paleoclimatological interpretation

J. Ricardo Mateos-Carralafuente[1], Ismael Coronado[2], Juncal A. Cruz[2], Pedro Cózar[3], Esperanza Fernández-Martínez[2], Sergio Rodríguez[1,3]

J. Ricardo Mateos-Carralafuente[1], Ismael Coronado[2], Pedro Cózar[3], Sergio Rodríguez[1,3]

[1]Department of Geodynamics, Stratigraphy and Paleontology, Faculty of Geological Sciences, Complutense University of Madrid, c/ José Antonio Novais, 12, 28040, Madrid (Spain): josericm@ucm.es.

[2]Faculty of Biological and Environmental Sciences, University of Leon, Campus Vegazana s/n, 24071 León, Spain: icorv@unileon.es

[3]Instituto de Geociencias (CSIC, UCM), c/ Severo Ochoa 7, 28040-Madrid (Spain).

https://orcid.org/0000-0003-2700-6747

https://orcid.org/0000-0002-8469-6003

https://orcid.org/0000-0003-2768-9242

https://orcid.org/0000-0002-4669-8702

https://orcid.org/0000-0002-2288-4113

https://orcid.org/0000-0002-4669-8702

Supplementary figures

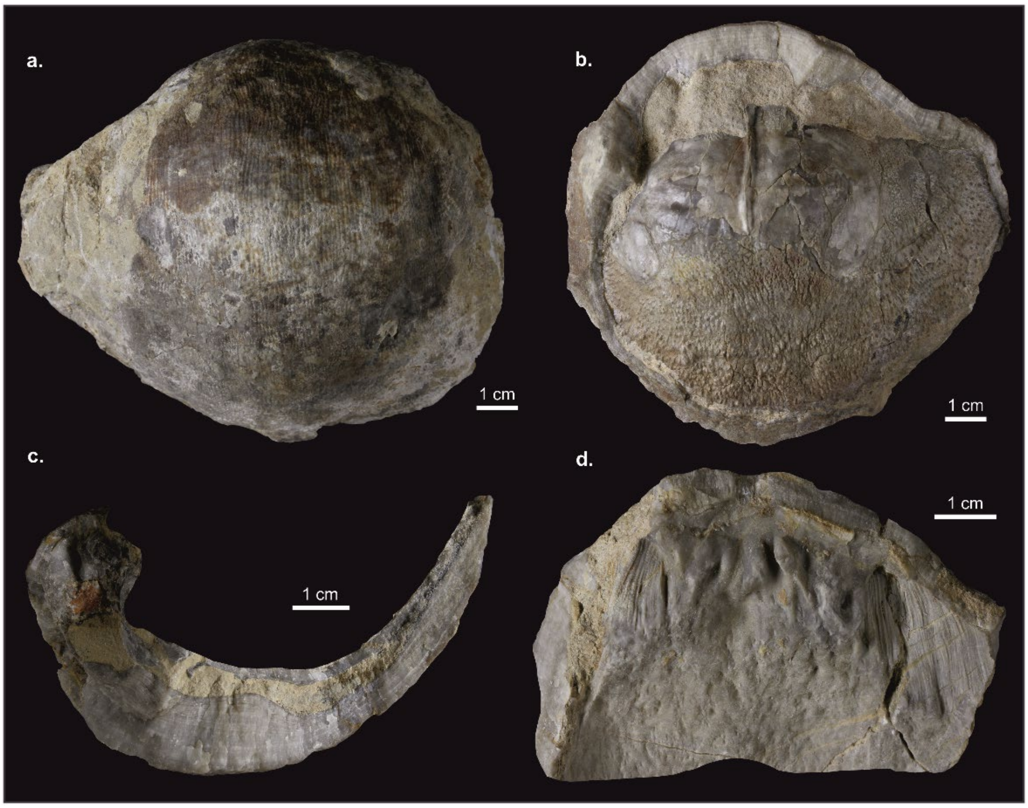

Figure S1 *Gigantoproductus* sp. shells. a) Concave ventral valve. b) Concave ventral valve, in which the pseudopuncta can be appreciated in the inner part of the prismatic layer. c) Longitudinal section showing the thickness of the ventral valve in comparison with dorsal valve. The cavity occupied by the organism is filled with micrite. d) Fragment of the ventral valve with preserved muscular impressions, teeth and sockets. Note the difference in size between muscles

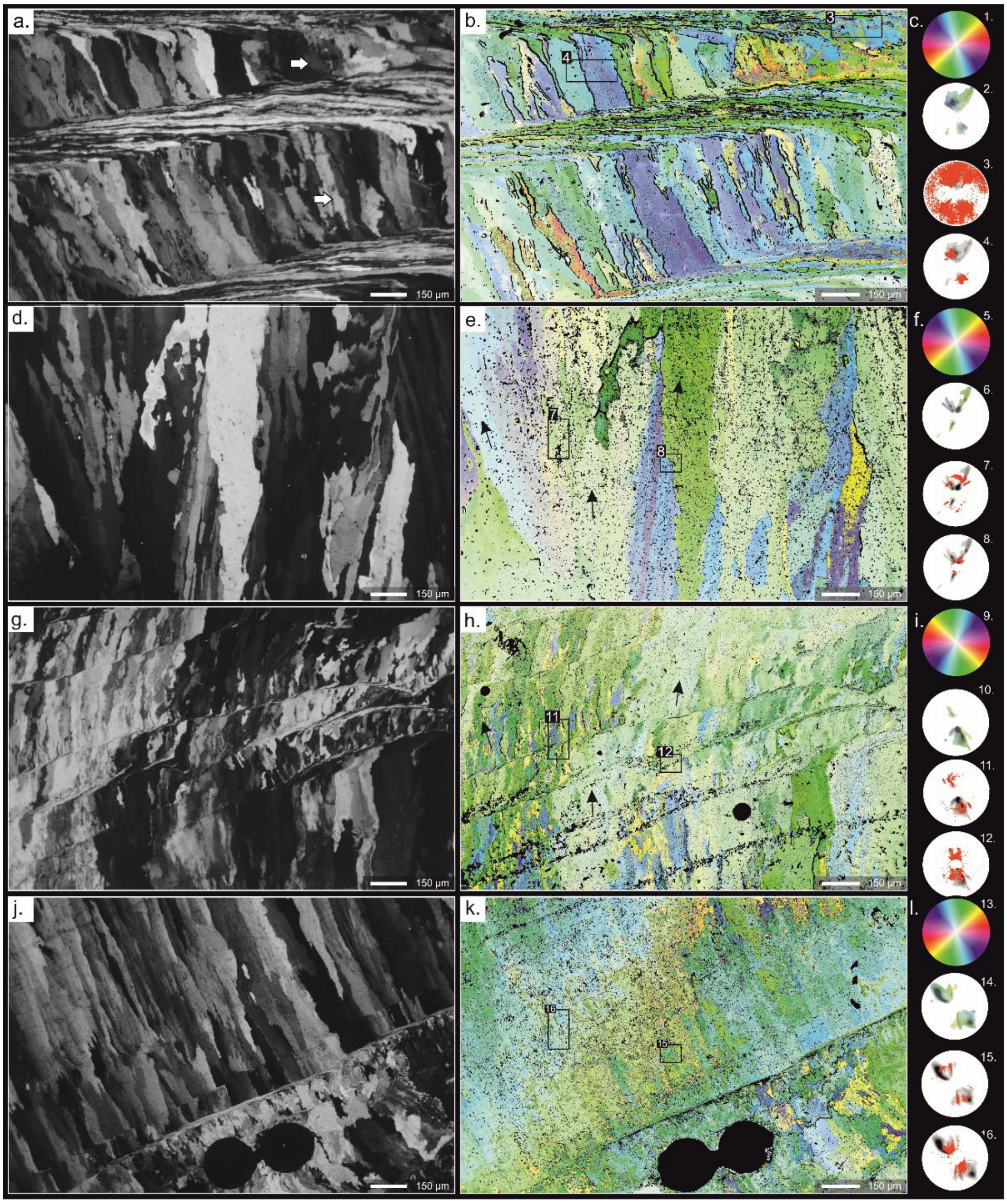

Figure S2 CIP analysis of thin region. (a-b) Umbonal region (d-e), thick region (g-k) of *Gigantoproductus* shells. a,d,g,j) Orientation images. b,e,h,k) Petrographic micrographs of the studied areas. c,f,i) Standard colour look-up table (CLUT) and pole figures. 1, 5, 9,13) CLUT of the orientation images. 2-4, 6-8, 10-12, 14-16) Pole figures. (2) Corresponds to the complete area (b), showing a pole maximum with *c*-axis is strictly oriented perpendicular to the growth direction of *Gigantoproductus* shell. (3-4) corresponds to selected areas. (3) Prismatic area with random *c*-axis orientations due to recrystallisation. (4) Equivalent area to (3) showing a well-constrained *c*-axis orientation. (6) Corresponds to the complete area (d), two narrow pole maxima, with an azimuthal dispersion between them of ca. 55°. (7-8) corresponds to selected areas. (7-8) two equivalent areas of the prismatic microstructure. (10) Corresponds to the complete area, two narrow pole maxima, with an azimuthal dispersion between them of ca. 55°. (7-8) corresponds to selected areas. (7-8) two equivalent areas of the prismatic microstructure. (10) Corresponds to the complete area, showing a unique pole maximum. (11-12) two equivalent areas of cut by a growth line (11) and a pseudopuncta.Pole figures were calculated as an orientation distribution function and provided in multiples of uniform distribution intervals of 0.5 for *c*-axis orientations of the complete area. Red points correspond to the punctual *c*-axis orientation of each pixel of selected areas. (14) corresponds to the complete area (j),

two narrow pole maxima, with an azimuthal dispersion between them of ca. 35°. (15-16) corresponds to selected areas. (15-16) two equivalent areas of the prismatic microstructure, showing differences in the immersion of *c*-axis. Note that the area below the laminar growth line exhibits a textural change produced by recrystallisation with random *c*-axis orientation

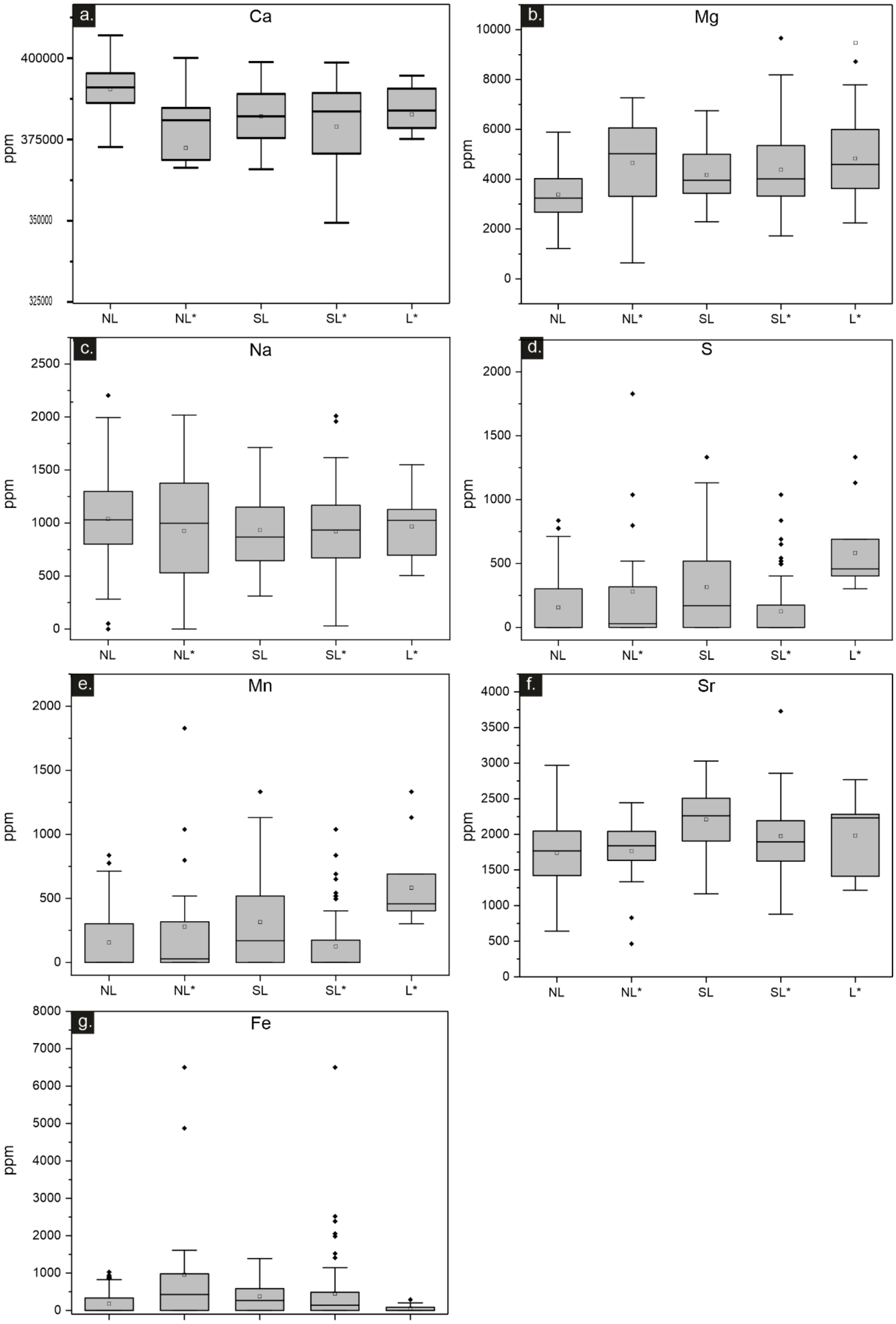

Figure S3 Boxplot of the seven analysed elements in well-preserved and poorly-preserved areas (*). Ca: Calcium; Mg: Magnesium; Na: Sodium; S: Sulphur; Mn: Manganese; Sr: Strontium; Fe: Iron. NL: Non-luminescent; SL: Slightly luminescent; L: Luminescent

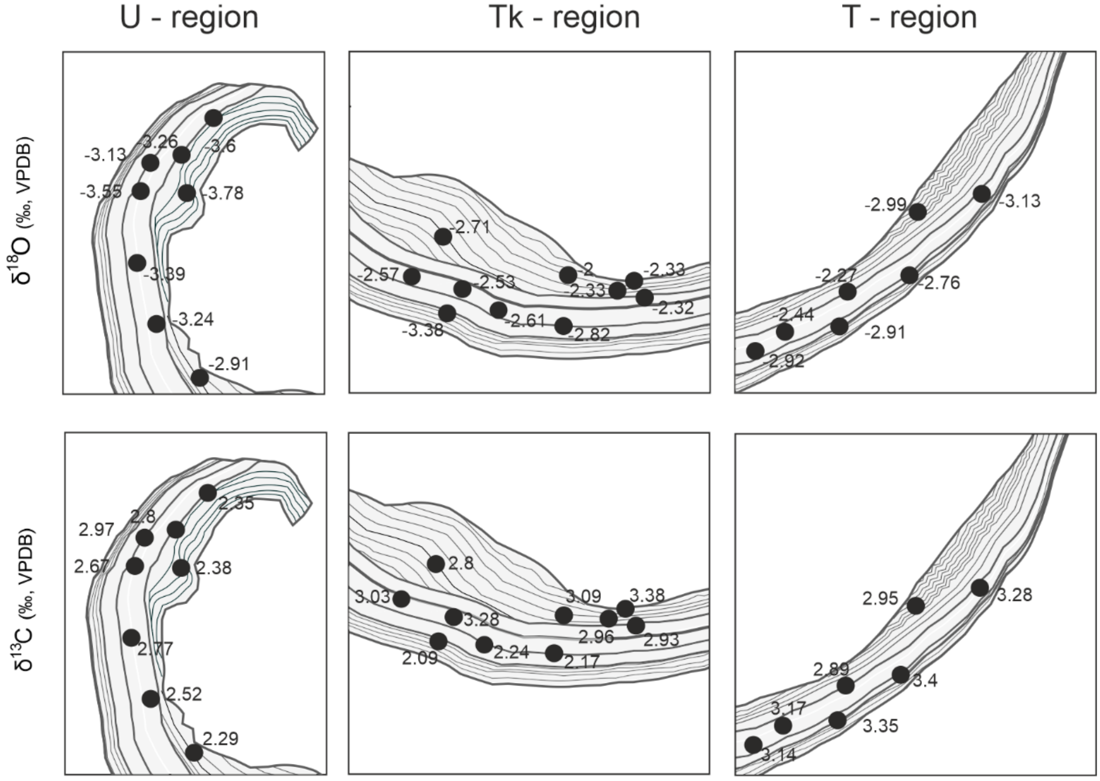

Figure S4 All drill locations sampled in the specimens over a brachiopod scheme to compare its relative position into the ventral valve. U: umbonal, Tk: thick; T: thin

References

Colmenero JR, Fernández LP, Moreno C, Bahamonde JR, Barba P, Heredia N, Gonzalez F. (2002) Carboniferous. In: Gibbons, W., Moreno, T. (Eds.), The Geology of Spain. Geol Soc, Bath, United Kingdom. 93–116. https://doi.org/10.1144/GOSPP.7

Cózar P. (1998) Bioestratigrafía con foraminíferos del Carbonífero Inferior del sector Norte del Área del Guadiato (Córdoba). Madrid: Universidad Complutense.

Cózar P, Rodríguez S, Somerville ID. (2003) Large multi-biotic cyanoliths from relatively deep-water facies in the early Serpukhovian of SW Spain. Facies. 49:31–48. https://doi.org/10.1007/s10347-003-0023-0
